# Woody plant encroachment enhanced negative interactions in Arctic plant communities over the last 24,000 years

**DOI:** 10.1038/s41467-026-77801-9

**Published:** 2026-09-22

**Authors:** Ying Liu, Weihan Jia, Sisi Liu, Simeon Lisovski, Kathleen R. Stoof-Leichsenring, Luca Zsofia Farkas, Ulrike Herzschuh

**Affiliations:** 1 Alfred Wegener Institute Helmholtz Centre for Polar and Marine Research, Polar Terrestrial Environmental Systems, 14473 Potsdam, Germany; 2 Institute of Environmental Science and Geography, University of Potsdam, 14476 Potsdam, Germany; 3 Institute of Biochemistry and Biology, University of Potsdam, 14476 Potsdam, Germany

**Keywords:** Palaeoecology, Biogeography, Community ecology, Biodiversity

## Abstract

Woody taxa encroachment in the Arctic has been widely observed. However, it remains uncertain how plant interactions are affected by such encroachment due to the lack of long-term observational data. Here, we reconstruct plant composition and functional trait turnover during post-glacial woody encroachment using sedimentary ancient DNA from nine lakes in the Siberia-Alaska region. Environmentally constrained null models are applied to partition plant interactions from the pure environment driven plant co-occurrence signal. Our results show that plant interactions shifted from predominantly positive interactions (e.g., nurse-plant facilitation) during the glacial period to more negative interactions (e.g., competition) during the Holocene. This shift coincided with a community transition from herbaceous to woody taxa, leading to an increase in average plant height and root length, as evidenced by leveraging plant trait information. We suggest that climate (an external factor) supported the widespread woody taxa expansion at the end of last glacial, which shifted the plant interaction (an internal process) that further exacerbated the expansion. This mechanism may provide an analogy with contemporary “arctic greening”. Furthermore, woody encroachment is likely to constrain the geographical ranges of native species, increasing the risk of local native taxa loss, while enhancing beta diversity.

## Abstract (web summary)

As Arctic climates warm, shrubs and trees spread into tundra, but long-term effects on plant communities are unclear. Using ancient DNA from nine Siberia–Alaska lakes, the study finds woody encroachment since the last ice age increased negative interactions between plant species.

## Introduction

Woody taxa expansion and increased biomass have been widely observed across the circumpolar region, which is referred to as Arctic greening^1–4^. This process of woody encroachment has been reported to reduce the diversity and cover of other functional groups^5,6^. However, certain shrub species also act as nurse plants, exerting positive interactions that facilitate the establishment and persistence of neighbouring taxa^7^. Yet, our understanding of these ecological dynamics remains constrained by short-term observations spanning only a few decades, leaving critical uncertainties regarding the long-term consequences of woody plant expansion. How will plant community turnover respond to Arctic warming? How will plant interactions evolve under sustained woody encroachment? What factors drive these shifts, and how do they, in turn, shape the plant community? Addressing these questions requires a long-term perspective and a deeper understanding of the mechanisms governing plant interactions in a changing Arctic.

According to the Stress Gradient Hypothesis, positive interactions among plants are more prevalent in stressful environments, whereas negative interactions dominate under more favourable conditions^8^. An empirical example of this hypothesis is cushion plants—low-growing, mat-forming genera such as *Eritrichium*, *Saxifraga*, and *Draba*—which survive in harsh Arctic and alpine environments^9–11^. These plants enhance local microhabitats by trapping heat, stabilising soil, and facilitating the establishment of other species within their crown areas^12,13^. However, empirical investigations of other plant interactions in Arctic ecosystems remain limited, constraining our ability to predict future shifts in community dynamics amid ongoing environmental change.

Plants interact with each other through phenotypic characteristics, which reflect strategies of resource acquisition and allocation, mediate interactions with neighbouring plants, and ultimately shape plant–plant dynamics^14^. Plant height is a key determinant in pre-empting light resources and enhancing competitive ability. Across much of the Arctic, plant height has been increasing in response to warming^15^, potentially intensifying competitive interactions. Similarly, root length, a critical trait governing belowground water and nutrient uptake^16^, may further drive competition as deep-rooted taxa expand. Different plant growth forms vary not only in plant height but also in root length, which can shape how the plants interact. However, how woody encroachment corresponds to community-level traits, and how these resulting shifts in traits shape community-wide interactions, are largely unknown. This highlights the need for further investigation into the connections between plant traits and interaction dynamics.

The shifts in plant communities and interactions from the glacial period (before 14 cal ka BP) to the Holocene (after 10 cal ka BP), driven by rising temperatures, may provide an analogue for contemporary ecological change in the Arctic. Northeast Siberia and Alaska, in particular, have experienced pronounced temperature shifts over millennial timescales due to polar amplification^17^. Furthermore, this region was largely free from ice sheets during the last glacial period^18^, minimising lagged species assembly effects as new habitats formed. High-resolution lake sediment archives, extending back to the Last Glacial Maximum (26-19 cal ka BP), combined with sedimentary ancient DNA (sedaDNA) method, provide robust datasets for reconstructing plant community dynamics^19^. Such datasets have enabled the reconstruction of shifts in the relationship between species richness and mean range size^20^, as well as patterns of taxa loss^21^. The higher taxonomic resolution of sedaDNA^22–24^, compared to pollen analysis, facilitates a more accurate reconstruction of taxon interactions through integrative network analysis^25,26^. Mathematical approaches provide an efficient means of disentangling the mechanisms underlying plant co-occurrence, thereby enhancing our understanding of species assembly and community dynamics. Environmentally constrained null models can partially account for environmental influences, dispersal limitations, and stochastic effects on species co-occurrence, attributing the residual co-occurrence to the effects of biotic interactions^27^. They provide insight into disentangling the plant interaction from plant co-occurrence, but this approach has not yet been applied to investigate long-term species interactions using proxy data.

In this work, we leverage sedaDNA to reconstruct plant community turnover over the past 24,000 years from nine lake sediment records across northeast Siberia and Alaska. To investigate the temporal dynamics of plant interactions throughout this process, we apply environmentally constrained null models to disentangle the effects of plant interactions from other factors shaping plant co-occurrence patterns. Further, we examine key functional traits, including plant height and maximum root length, to assess their roles in mediating the plant interactions.

## Results

### Postglacial woody taxa expansion in response to warming

**Fig. 1:**
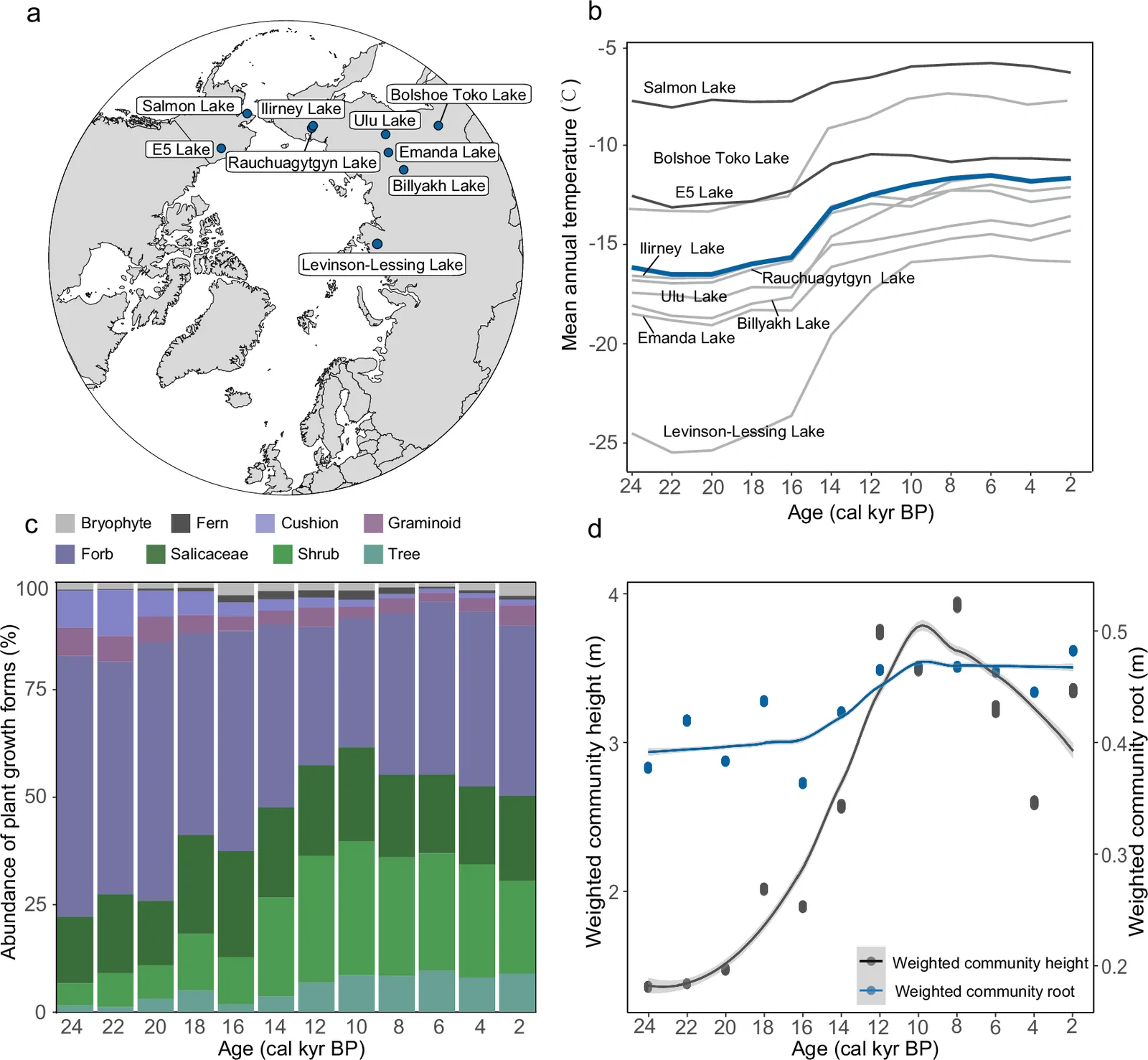
Mean annual temperature, plant growth-form abundance, and community weighted trait over time. **a** Locations of the nine lakes in northeastern Siberia and Alaska. **b** Temporal variations in mean annual temperature (see Methods). The thin dark grey curves show site-specific mean annual temperatures in the Alaska region, the light grey curves show site-specific mean annual temperatures in the Siberia region, whereas the bold blue curve represents the mean across all study sites. **c** Temporal dynamics in the proportion of transformed counts, representing the relative abundance of distinct plant growth-forms, are depicted in different colours. Within the shrub growth form, Salicaceae was shown specifically due to its high read counts. **d** Temporal changes in community-weighted traits. Points represent the community-weighted trait values from the 100 resampling iterations, lines represent LOESS-fitted curves, and shaded areas indicate 95% confidence intervals. Community-weighted traits were calculated as the sum of ASV-level trait values weighted by their relative abundances. Source data are provided as a Source Data file.

The study area covers northeastern Siberia and Alaska (Fig. 1a). We reanalysed a sedaDNA dataset originally generated by Courtin et al.^21^, Jia et al.^28^, and Herzschuh et al.^19^. All data use metabarcoding approaches targeting the P6 loop of the *trn*L (UAA) intron. Following data filtering^21,28^, only samples originating in the past 24,000 years and amplicon sequence variants (ASVs) with 100% identity to the customised regional SibAla_2023 database^21^ were retained (Fig. 1). The final dataset consists of 369 samples, generating a total of 78,088,278 reads, representing 625 ASVs.

We divided the timeline into 12 time slices, each spanning 2000 years. For each time slice, the mean annual temperature at each study site was downscaled using the algorithm developed by Karger^29^, based on the input data from Kleinen et al. (2023)^30^ (see Methods). The temperatures in the study region remained relatively low during the glacial period (before 14 cal ka BP), increased during the transition phase, and reached relatively high levels during the Holocene (after 10 cal ka BP) (Fig. 1b). Spatially, temperature shows a decreasing trend from the Alaskan lake region toward the Siberian lake region (Fig. 1b).

Relative abundance data indicate a woody taxa encroachment into the plant community from the glacial period to the Holocene (Fig. 1c; Supplementary Fig. 1). The community was dominated by herbaceous taxa prior to 14.0 cal ka BP, with forbs comprising approximately 60% of the total plant abundance, while cushion plants and graminoids each accounted for less than 10% (Fig. 1c). In contrast, woody taxa were comparatively sparse, with a total abundance below 30%. After 14.0 cal ka BP, woody taxa increased in abundance, with Salicaceae rising moderately from 15% to ~20%. Concurrently, other shrub taxa expanded rapidly, peaking at 31% at the onset of the Holocene (~10.0 cal ka BP). Tree taxa also show a slight increase, reaching approximately 9%. In contrast, cushion plants and graminoids declined gradually, both falling below 5%. Forbs underwent a marked decline, reaching a minimum of 30% around 10.0 cal ka BP, before gradually recovering.

Two community-level plant functional traits associated with competitive ability—light acquisition (plant height) and resource absorption (maximum root length)—were inferred from sedaDNA-derived data. The results indicate that community-weighted plant height increased between ~16 and 14 cal ka BP, reaching nearly 3 m. It continued rising and peaked at approximately 4 m during the early Holocene (10–8 cal ka BP), followed by a slight decline while remaining above glacial levels (Fig. 1d). In parallel, community-weighted root length began increasing around 14 cal ka BP, reached its peak in the early Holocene (10–8 cal ka BP), at around 0.47 m, and remained relatively high thereafter (Fig. 1d).

### Environmentally constrained plant interaction network

We used species distribution models (SDMs) to predict plant occurrence patterns driven by temperature, incorporating a random effect to account for stochastic variation beyond temperature. The C-score, derived from species occurrences in sedaDNA data, was compared with that obtained from environmentally constrained null models (SDM-constrained). The plant co-occurrence patterns from sedaDNA with a C-score significantly different from the null model were retained (Methods for details). To account for the potentially confounding effect of dispersal limitation, we examined spatial range overlap between species pairs. Those with disjunct or partially overlapping distributions were interpreted as being constrained by dispersal. By systematically ruling out temperature filtering, dispersal limitation, and stochasticity, we attribute the residual co-occurrence patterns to direct plant–plant interactions, providing insights into their temporal dynamics. The overlap between the inferred plant interactions and the Globi plant interaction database is approximately 26.0%, which is significantly greater than that observed in random plant interactions.

**Fig. 2:**
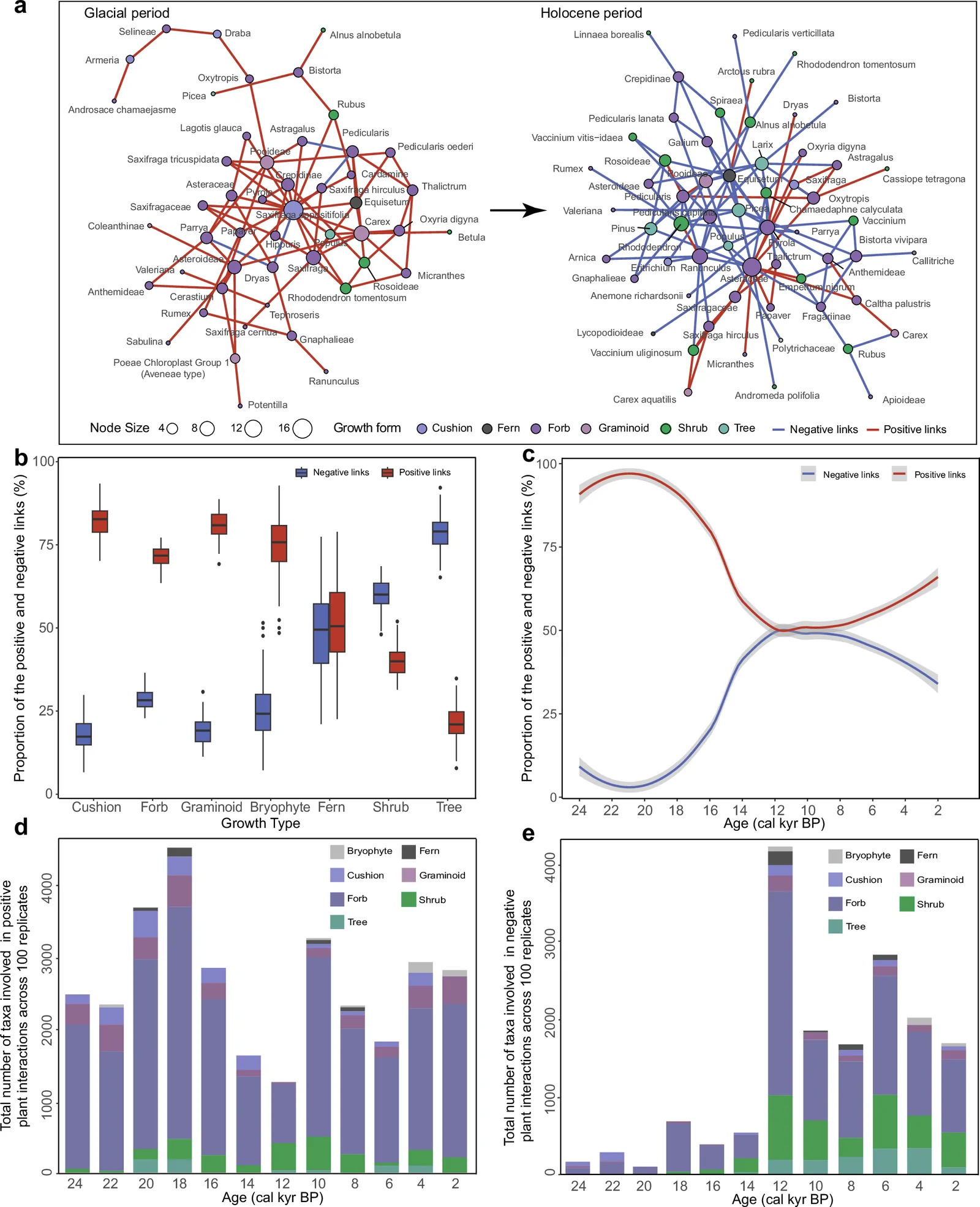
Dynamic plant interaction network during the glacial and Holocene periods. **a** The left panel shows a representative network from 20–18 cal ka BP, illustrating the pattern of the glacial period, while the right panel displays a network from 6–4 cal ka BP, representing the Holocene pattern. Nodes represent taxa, with different growth-forms indicated by colour. Node size corresponds to the number of connections (i.e., node degree). **b** Boxplots of positive and negative interaction percentages across growth-form taxa based on 100 resampling iterations. The center line indicates the median (50th percentile); the lower and upper bounds of the box represent the 25th and 75th percentiles, respectively; whiskers extend to the minimum and maximum values within 1.5 times the interquartile range from the box; and points beyond the whiskers represent outliers. **c** Temporal trends in the relative proportion of positive and negative interactions. Lines represent LOESS-fitted curves based on 100 resampling iterations, and shaded areas indicate 95% confidence intervals. **d** Temporal variation in the number of taxa with different growth-forms engaged in positive interactions. **e** Temporal variation in the number of taxa with different growth-forms involved in negative interactions. Source data are provided as a Source Data file.

Across all time slices, cushion plants exhibit a higher proportion of positive interactions, with a median of ~60% positive to ~40% negative interactions. In contrast, woody taxa, including trees and shrubs, are predominantly associated with negative interactions, comprising 70–95% of their total links. From the perspective of taxa composition within the plant–plant interaction network, forbs contribute the highest number of species, which are involved in both positive and negative interactions (Fig. 2a, d, e). Among other herbaceous groups, cushion plants and graminoids are predominantly engaged in positive interactions, particularly during the glacial period (24.0–14.0 cal ka BP). In contrast, woody taxa participate in both positive and negative interactions, with a marked shift toward predominantly negative interactions during the Holocene period (after 14.0 cal ka BP), for example, *Larix*.

From the perspective of interaction direction, positive plant interactions dominated during the glacial period, peaking around the Last Glacial Maximum (20–18 cal ka BP), and involving a large number of taxa, for example, *Saxifraga oppositifolia*, *Saxifraga tricuspidata*, and *Dryas* (Fig. 2a, d, Supplementary Fig. 2). By ~14.0 cal ka BP, negative interactions began to increase (Fig. 2a, e), reaching equilibrium with positive interactions at the onset of the Holocene (ca. 10 cal ka BP). Thereafter, negative interactions became more common but decreased again during the Late Holocene (Fig. 2c, Supplementary Fig. 2).

### Potential drivers of the negative link proportion

**Fig. 3:**
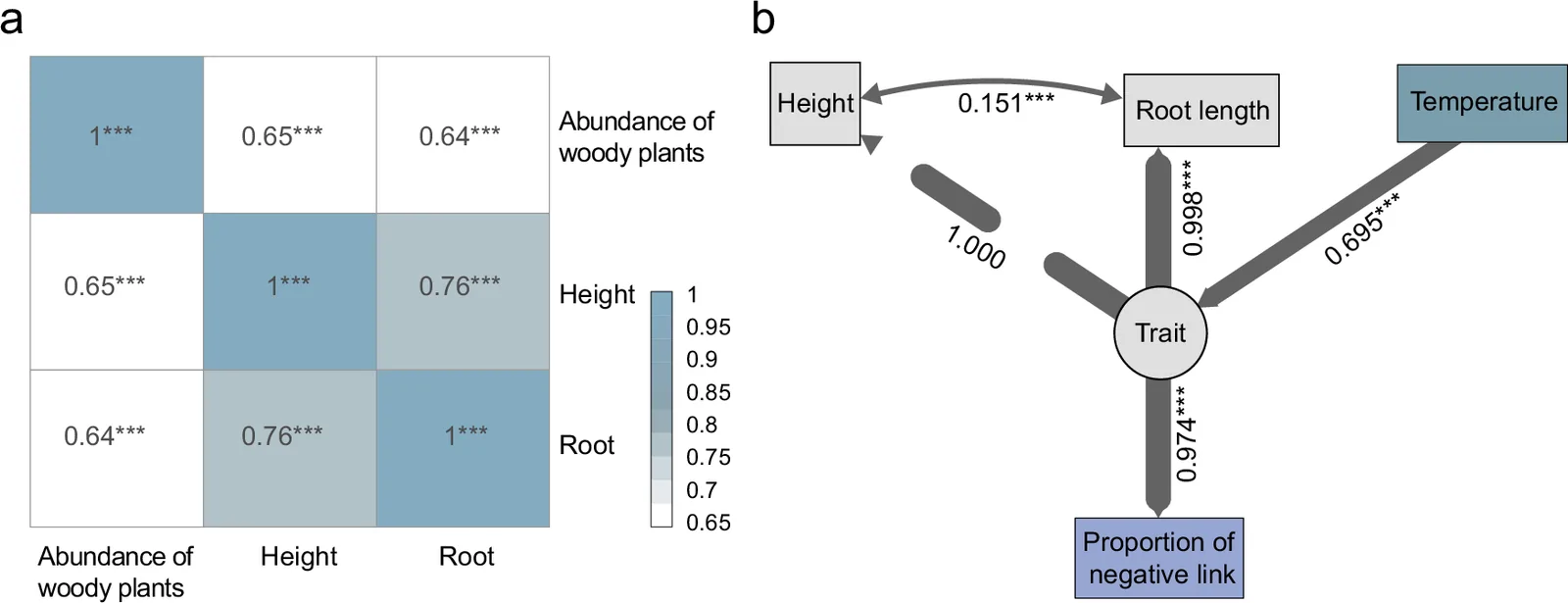
Structural equation model showing the relationships between response variables and predictors. **a** Pairwise correlation matrix among woody taxon abundances and two plant functional traits. Pearson correlation coefficients were calculated using two-sided Pearson correlation tests, with *P* values adjusted for multiple comparisons using the Benjamini–Hochberg procedure. The adjusted *P* values for correlations between woody taxon abundance and height, woody taxon abundance and root, and height and root were 3.37 × 10⁻¹⁴³, 2.16 × 10⁻¹³⁹, and 1.16 × 10⁻²²⁷, respectively. Asterisks indicate adjusted *P* values (*P < 0.05, **P < 0.01, *P < 0.001). **b** The structural equation model illustrates the effects of temperature and plant traits on the proportion of negative links. Variables are colour-coded by origin: dark blue for temperature, light grey for plant functional traits, and light bluish purple for the proportion of negative interactions. ‘Trait’ was modelled as a latent variable, inferred from two observed indicators: height and root length. Arrows originate from the latent construct ‘Trait’ and point toward its indicators, height and root length, representing factor loadings. The factor loading of height on the latent construct was fixed to 1, indicated by a dashed arrow. Double-headed arrows represent covariances between observed variables, single-headed arrows indicate regression paths. Arrow thickness reflects the magnitude of the standardised coefficients. Standardized path coefficients and covariances are displayed alongside the corresponding paths. The statistical significance of model parameters was assessed using two-sided z-tests based on maximum likelihood estimation, with asterisks denoting significance levels (*P < 0.05, **P < 0.01, ***P < 0.001). Model fit was evaluated using the chi-square statistic (χ²), RMSEA, CFI, GFI, and SRMR (χ² = 0.266, df = 1, *P* = 0.606; RMSEA = 0; CFI = 1.00; GFI = 1.00; SRMR = 0.001). Source data are provided as a Source Data file.

A structural equation model was used to assess the direct and indirect effects of temperature and plant community functional traits on the proportion of negative plant interactions. The model was based on time-series data for mean temperature, community functional traits, and the proportion of negative interactions across all sites (*n* = 1200). Because of strong collinearity with community mean height and root length (Fig. 3a), woody plant abundance was excluded from the final model to improve parameter stability and model fit. The model has a good fit to the data, as indicated by the fit metrics: chi squared (χ²) = 0.3, degrees of freedom (df) = 1, *p* = 0.6, Root Mean Square Error of Approximation (RMSEA) = 0.0, Comparative Fit Index (CFI) = 1.0, Goodness of Fit Index (GFI) = 1.0, and Standardised Root Mean Square Residual (SRMR) = 0.001. The results reveal that community plant traits responded strongly to increasing temperatures, with plants growing taller and developing longer roots, which in turn led to more negative plant interactions.

## Discussion

### Plant interactions inferred from the sedaDNA-based co-occurrence networks

We find that herbaceous plants are involved in more positive plant interactions, whereas woody taxa are associated with more negative interactions across all time slices. The observed plant interactions across different growth forms align with modern ecological expectations. Cushion plants, known to act as nurse species in harsh environments, exhibit predominantly positive interactions with neighbouring plants, such as facilitation and cooperation^9,13,31^. Consistent with these observations, our co-occurrence analysis reveals that cushion plant taxa form a higher proportion of positive than negative links. In contrast, woody taxa, particularly trees, are more frequently associated with negative interactions, consistent with reports of their competitive interactions with neighbouring taxa^6,32^. The consistent interaction patterns across different growth-forms further support the robustness of the identified plant interaction network.

We inferred reasonable plant interactions from environmentally constrained co-occurrence networks following protocols applied to modern plant co-occurrence, such as those sampled along a broad elevational gradient in the Swiss Alps^27^, but new to palaeoecological studies. This analysis incorporates the potential influences of climate, dispersal limitations, and stochastic processes^27^. Furthermore, the overlap between the inferred plant interactions and the Globi plant interaction database is significantly greater than that observed in random plant interactions, thereby supporting the reliability of the inferred plant interactions.

Our analysis, however, still has certain limitations. One source of the limitation is the models used in our analysis. The climatic effects were estimated using a species distribution model (SDM), which has inherent uncertainties, such as limited species occurrence records, potentially retaining false links or omitting true ones. Additionally, taxa confined to specific microhabitats within a catchment may be poorly represented in SDMs, leading to inaccurate presence–absence predictions. In addition, pairwise calculations may overestimate associations driven by covariation. Such associations may vanish when additional species interactions are considered^33^, potentially allowing indirect interactions to be misinterpreted as direct ones. Nevertheless, pairwise analyses reduce the risk of overlooking potential interactions that may be missed when using partial correlations. Lastly, negative interactions may be underestimated, as strong competition can prevent co-occurrence or drive species exclusion.

On the other hand, our study infers plant interactions from DNA samples, using a maximum of 18 samples per 2000-year time slice. To enhance the robustness and resolution of network inferences, larger sample sizes are required^34^. Although a 2000-year time slice reduces age–depth uncertainty and increases sample size relative to shorter intervals, and plant composition within such windows is broadly homogeneous, it risks introducing false co-occurrences that may overrepresent positive plant interactions. Additionally, taphonomic processes affecting DNA preservation in lake sediments may also bias the representation of the original plant community^35,36^. Although the use of presence-absence data mitigates some of these biases, it does not fully eliminate them. Moreover, the limitations in amplicon sequence variants (ASVs) resolution prevent the precise taxonomic identification of all taxa at the species level. In the resampled dataset, 55.2% of ASVs were identified at the species level, 31.2% at the genus level, and 13.6% at the family level. A single ASV may correspond to multiple taxonomically related species but is represented by only one interaction pair, potentially leading to an underestimation of interaction links. Lastly, sedaNDA captures genetic signals from the entire catchment, potentially detecting these localised taxa and producing spurious co-occurrence patterns with other species, thereby overestimating the plant interactions.

### Effects of woody encroachment on plant interaction shifts

The proportion of negative plant taxa interactions increases with woody taxa encroachment. During the glacial period (before 14 cal ka BP), the study region remained largely ice-free^18,37^, providing the required habitat for species survival. Simulations and proxy-based temperature reconstructions suggest relatively low temperatures during this period^30,38^. Harsh climate conditions likely acted as an environmental filter, favouring the persistence of cold-adapted herbaceous taxa, including forbs, cushion plants, and graminoids. The survival of woody taxa was restricted to specific refugial sites with favourable microclimatic conditions^28,39–41^. The plant interactions were mainly positive, with most taxa being herbaceous. As the climate warmed from the glacial period to the Bølling–Allerød Interstadial (14–12 cal ka BP), the study area became increasingly favourable for relatively thermophilic species (Fig. 1b), promoting the expansion of woody taxa. Key taxa contributing to this process include willow (*Salix* spp.), alder (*Alnus* spp.), birch (*Betula* spp.), and *Vaccinium* spp. With woody taxa encroachment, negative plant interactions increase, involving both woody and herbaceous species.

Plants interact through their phenotypic characteristics, and changes in plant functional traits can drive shifts in these interactions^42^, with the greater height of encroaching woody taxa potentially contributing to aboveground light competition. Erect shrubs and trees, such as *Alnus*, *Betula*, *Salix*, *Picea*, *Abies*, and *Larix*, are on average taller than herbaceous plants^43^, which, when coupled with relatively favourable conditions, enable them to achieve greater heights. Their encroachment contributed to an overall increase in plant height during the Bølling–Allerød Interstadial. This, in turn, raised the community-weighted plant height, with woody taxa intensifying light competition and reducing light availability for low-lying plants^44,45^. Such changes can lead to etiolation (the process by which plants exhibit elongated growth, pale colouration, and poor health) or exclusion, particularly for shade-intolerant species^46^. The plant height peaked in the early Holocene, which was followed by a slight decline due to the expansion of dwarf shrubs.

The expansion of woody taxa, characterised by their relatively long root systems, likely enhanced belowground competition for nutrients and space. Root system architecture is a key functional trait governing water and nutrient acquisition^16^. Coinciding with the expansion of woody vegetation, relatively long root length likely intensified belowground competition for resources and space, for example, for *Picea* and *Pinus*^43,47^. Additionally, their long-lived root systems facilitate the formation of mycorrhizal associations by providing fungi with suitable hosts and continuous access to photosynthates^48^, which in turn enhance nutrient and water uptake by the plants. For example, ectomycorrhizal fungi are commonly associated with the roots of Betulaceae, Salicaceae, and Rosaceae, while ericoid mycorrhizas form symbiotic relationships with members of Ericaceae, such as *Vaccinium vitis-idaea* and *Cassiope tetragona*^49^. Consequently, community-level root length increased during the Bølling–Allerød interstadial and remained elevated throughout the Holocene, suggesting intensified belowground competition for resources within plant communities.

The structural equation model identifies key drivers of negative plant interactions (Fig. 3). As temperatures rise, the increasing abundance of woody taxa enhances community canopy height and root length, intensifying competitive interactions. While temperature indirectly influences these dynamics, shifts in functional traits associated with woody expansion serve as the direct driver of increased negative interactions.

Some woody taxa shift from acting as nurse plants, which primarily interact positively, to becoming competitors with woody taxa encroachment. Plant interaction networks suggest that positive interactions are largely confined to forbs and other herbaceous taxa. Certain woody species also contributed to facilitation and cooperation within plant communities during glacial periods, in contrast to the predominantly negative interactions of woody taxa during interglacial periods. Among them, some shrubs have been documented as nurse plants, alleviating extreme microenvironmental stresses such as drought, elevated temperatures, and soil surface instability^50^. For instance, the dwarf shrubs *Cassiope tetragona* and *Empetrum nigrum* have been shown to provide aboveground shelter^51^, as indicated by the increased shoot height of taxa growing in their proximity. Both taxa show mainly positive interactions during the glacial period. This effect is likely driven by enhanced snow cover, protection from strong desiccating winds early in the growing season, and a warmer microclimate^51^. However, during the Holocene, plant interactions shifted to include negative interactions, likely driven by competition for light and resources, as well as potential disruptions to germination.

This pattern aligns with the Stress Gradient Hypothesis, which posits that positive interactions dominate under stressful conditions, while competition becomes more prevalent in favourable environments^8,52,53^. Such conditional interaction strategies of shrub species may maximise their fitness across varying environments. Facilitative interactions likely enhanced their survival during glacial periods by buffering harsh environmental stresses, while the shift towards competitive interactions in the Holocene may have allowed them to acquire resources in less stressful environments. This ability to switch between facilitation and competition may have supported their persistence across both glacial and interglacial periods.

### Potential effects of plant interactions on the past and future plant community

**Fig. 4:**
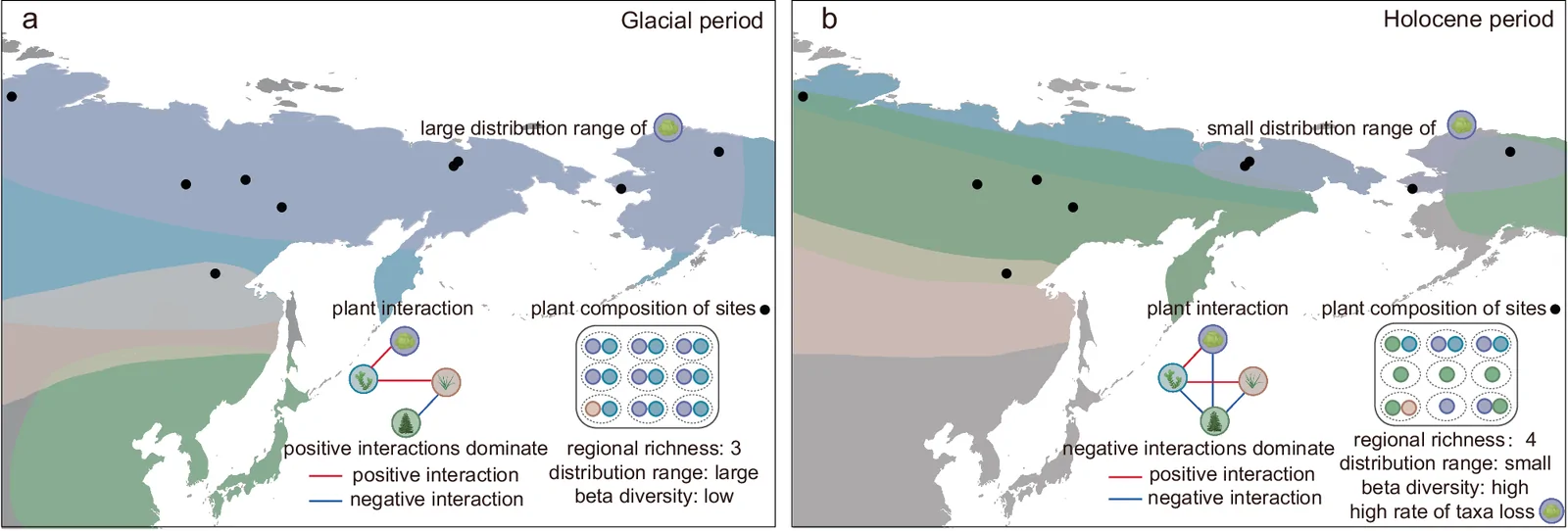
Schematic diagram depicting the impact of plant interactions on communities. **a** The glacial period scenario. **b** The Holocene scenario. The coloured polygons represent the distribution ranges of different species, which are presented in the plant interaction network. Red lines indicate positive plant interactions, while blue lines denote negative interactions. Black dots mark the nine sites from which sedaDNA data were obtained, with each site’s plant composition visualised within an ellipse. The combined results from all nine sites form the regional species composition, which is enclosed within a rounded rectangle that contains all the ellipses.

Intensified negative plant interactions resulting from woody taxa expansion further influence the plant community. During the glacial period (Fig. 4a), the positive interaction likely facilitated the establishment of new taxa and supported the survival of species that might have otherwise been constrained by environmental conditions^54^. However, this also rendered the plant community more vulnerable to invasions by other taxa. Positive interactions may have further contributed to the persistence of resident taxa by promoting their geographical range expansion. Our previous study found that, during the glacial period, the average range size of plant communities increased, with species facilitating each other’s distribution^20^. For instance, *Silene acaulis*, which engages in positive interactions with multiple taxa, played a role in expanding species’ ranges^9^. As a result, regional beta diversity remained low, as the widespread distribution of species promoted a more homogeneous plant composition. Furthermore, the positive plant interactions and relatively wide distribution range likely prevented the plant from going extinct, sustaining diversity even under harsh environmental conditions^55,56^.

In contrast, during the interglacial period (Fig. 4b), intensified negative interactions may constrain the establishment of new taxa by favouring native species with competitive advantages^57^. As a result, this area exhibited increased resistance to species migration, likely mediated through competition for light and resources, for example, for *Larix*, as well as the release of allelopathic chemicals^58^. Shifts in plant–plant interactions may also influence the persistence of resident taxa, as stronger negative interactions could suppress population growth rates, restricting range expansion and potentially leading to range contraction^20,59,60^. As a result, these species face an increased likelihood of local extirpation, consistent with the findings of Courtin et al.^21^, who find a higher than expected loss of taxa, ultimately leading to a decline in diversity. Moreover, herbaceous taxa, due to their traits, are less competitive and particularly vulnerable to local extinction in the study region (Fig. 4b, species marked in purple circles), as noted by Courtin et al.^21^. However, regional beta diversity may increase as competition fosters divergent community assemblages across sites (Fig. 4b), enhancing spatial heterogeneity at broader scales^61^. Furthermore, our findings suggest that species with advantageous traits under negative interactions are more likely to become dominant in plant communities^62^ (Fig. 4b, woody species marked in green circles).

Overall, while most species distribution models emphasise the direct effects of abiotic factors, biotic interactions such as competition can modify or even counteract the direct impacts of climate on species performance^63^. With global warming, woody taxa are likely to dominate Arctic plant communities due to their advantageous competitive traits, which facilitate their establishment, expansion, ultimately contribute to their widespread geographical distribution. In contrast, native taxa with lower competitive ability, such as herbaceous taxa with shorter stature or shallower root systems, warrant greater attention, as they may decline or disappear before environmental conditions exceed their physiological tolerance, driven by negative interactions. Arctic plant community composition is shaped by more than just the northward migration of lower-latitude species, as biotic interactions may prevent certain herbaceous plants from establishing. Plant diversity in the Arctic will be determined by the dynamic balance between species gains and losses. Accordingly, regional beta diversity is driven by variation in species gains and losses across sites.

## Methods

### The sedaDNA dataset

We used previously published sedimentary ancient DNA (sedaDNA) data from nine lake sediment cores collected in northeastern Siberia and Alaska (Fig. 1a), originally reported by Courtin et al.^21^, Jia et al.^28^, and Herzschuh et al.^19^. The laboratory procedures used to generate these data have been described previously^21^. In brief, 3–5 g of sediment was processed using the PowerMax® Soil DNA Isolation Kit (Qiagen, Germany) to extract sedaDNA. The sedaDNA was subsequently concentrated and purified using the GeneJET PCR Purification Kit (Thermo Fisher Scientific), and amplified with g and h primers (g primer, GGGCAATCCTGAGCCAA; h primer, CCATTGAGTCTCTGCACCTATC) targeting the P6 loop of the chloroplast *trn*L (UAA) intron^64^. PCR products were purified using the MinElute PCR Purification Kit (Qiagen). All procedures from extraction to PCR amplification, purifying, and merging were conducted in the palaeogenetic laboratories of the Alfred Wegener Institute, Helmholtz Center for Polar and Marine Research, Potsdam. Finally, the PCR replicates were pooled equimolarly and sequenced in paired-end mode (2 x 150bp) on a HiSeq or NextSeq Illumina platform run by the Fasteris Genesupport SA sequencing service (Switzerland).

After DNA sequencing, the raw sequencing files were quality checked and paired-end reads were merged, further sequences were sorted according to samples and taxonomically assigned to the customized SibAla_2023 database^21^. All steps were performed using OBITools v3^65^. Only Amplicon Sequence Variants (ASVs) with 100% matches to the customized “SibAla_2023” database were included in the analysis. Further quality control was conducted based on PCR replicability: PCR replicates were excluded if they showed substantial divergence from other replicates within the same sample, indicating low quality, or if replicates failed to cluster, suggesting distinct compositions within replicates^21^.

The age of each subsample was determined using Bayesian age-depth modelling from previous studies^28,66–73^, incorporating all samples spanning from 24 cal ka BP to the present. The sedaDNA dataset was divided into 24 time slices, each spanning 1000 years. To address variations in read counts across lakes and time slices, datasets were rarefied to a base count of 10,000 and resampled 100 times. This base count is lower than the minimum read count observed across all lakes within any given time slice, ensuring the retention of the majority of the original read information. Based on the 100 resamplings, temporal changes in regional relative plant abundance were represented by the proportion of transformed read counts, visualised with the ggplot2 (3.5.2) R package^74^, and presented in Fig. 2c.

### Temperature downscaling from the model

Palaeoclimate simulations for mean annual temperature and mean July temperature were obtained from the Max Planck Institute Earth System Model (MPI-ESM1.2), covering the last 23 ka BP^30,75^, with a spatial resolution of approximately 3.75° × 3.75°. The raw temperature data were subsequently downscaled to a resolution of 5 km × 5 km, following the method outlined by Karger et al.^29^. Briefly, the lapse rate (Γ) was calculated using the average temperature at six pressure levels ranging from 1000 hPa to 600 hPa:

1 $$\varGamma=\frac{6(\sum {a}_{z}{{ta}}_{z})-(\sum {a}_{z})(\sum {{ta}}_{z})}{6(\sum {{a}_{z}}^{2})-{(\sum {a}_{z})}^{2}}$$

where $t{a}_{z}$ is the average temperature at the geopotential height ${a}_{z}$ and the pressure level Z (6 levels from 1000 hPa to 600 hPa). The palaeo-elevation data was calculated by subtracting past sea level changes^29^ from modern elevation^76^.

The downscaled temperature at time slice t (${ta}{{s}_{t}}^{{ds}}$) was then calculated by:

2 $${ta}{{s}_{t}}^{{ds}}={\varGamma }_{t}\cdot {e}_{t}+{ta}{{s}_{t}}^{{raw}}$$

where ${\varGamma }_{t}$, ${e}_{t}$ and ${ta}{{s}_{t}}^{{raw}}$ are lapse rate (°C/m), palaeo-elevation (m), and raw temperature (°C) at timeslice t, respectively. The raw temperature data have a monthly temporal resolution, whereas this study requires temperature data at a thousand-year resolution. Therefore, the raw data were divided into 100-year intervals. For each interval, the last year was selected as the representative year, and each month’s temperature data of that year was used to calculate the spatially downscaled temperature. The mean annual temperature for the representative year was calculated as the average of its 12 monthly temperatures. January and July temperatures were taken directly from the corresponding monthly data. Finally, the mean of the ten representative years was used as the final value for each thousand years.

Then the downscaled temperature data were cropped to the ‘SibAla’ region (55–90°N, 50–150°E and 40–90°N, 150°E–140°W). To reduce biases in the palaeoclimate simulations, MPI temperatures at 0 ka were adjusted using the modern CHELSA V2.1 climate dataset^77^. The discrepancies between MPI and CHELSA temperatures at 0 ka were then used to calibrate temperature estimates for other time slices. For areas exposed by sea-level decline during glacial periods (e.g., the Beringia land bridge), temperatures were estimated using K-nearest neighbour interpolation. Ice-sheet cover was excluded from the climate data for all time slices. Finally, downscaled temperature data from the grid cells containing the nine lakes were extracted for subsequent analyses. Mean annual temperature was visualised across 2000 timeslices using the ggplot2 (3.5.2) R package^74^ and presented in Fig. 2b.

### Species distribution model

Species associated with the ASVs detected through sedaDNA data were retrieved from the Global Biodiversity Information Facility (GBIF; https://doi.org/10.15468/dd.y4uq5q)^78,79^ for the ‘SibAla’ region (55–90°N, 50–150°E and 40–90°N, 150°E–140°W). Records were filtered by removing occurrences (i) unidentified to species level, (ii) fossil specimens, (iii) those with a coordinate resolution <5 km, and (iv) records prior to 1981. As each ASV in the “SibAla_2023” database may correspond to one or more species, the modern distribution of each ASV was determined based on the combined distributions of all associated species and spatially thinned to 15 km resolution, where each 15 km × 15 km grid cell was assigned a binary presence/absence value. If multiple occurrence records were present within a single grid cell, one was randomly selected. ASVs with fewer than 100 occurrences across all grid cells were excluded from modelling due to insufficient sample size.

To define background data for presence-only species distribution modelling, we delineated an inner (25 × 25 km) and outer (200 × 200 km) buffer around each surveyed site. The outer buffer was used to approximate the locally accessible/sampled region within which occurrences were plausibly recorded given the strongly spatially biased observation footprint in the Arctic. The inner buffer was excluded to avoid selecting background points from the same (or immediately adjacent) 15 km × 15 km grid cells as occurrence records. Background points were sampled from the annulus between the inner and outer buffers and represent available environmental conditions within the sampled domain; they were not interpreted as confirmed absences.

Within this annulus, we generated a pool of candidate background points and applied an environmental filtering step to increase contrast between occurrences and background conditions. Specifically, we trained a one-class support vector machine (OCSVM; e1071::svm)^78–81^ on the climate conditions at occurrence locations (mean annual temperature and July temperature) and retained candidate background points classified as environmentally dissimilar to the occurrence environments. From this filtered pool, we randomly selected a number of background points equal to the number of occurrence grid cells for each ASV.

As a sensitivity analysis, we repeated the modelling using background points sampled randomly from the same annulus (i.e., without OCSVM filtering), again matching the number of background points to the number of occurrence grid cells; results are reported in the Supplement (Supplementary Figs. 13, 14).

Given their important roles in shaping Arctic plant distributions, community composition, and delineating bioclimatic zones^82^, mean annual temperature, mean July temperature, and mean January temperature, were obtained from the CHELSA v2.1 climate dataset^83,84^. These data were used to build the species distribution model, which demonstrated that mean July temperature and mean annual temperature were the two primary variables contributing to species distributions. Therefore, these two variables were selected for use in the final model. They were resampled to 5 km × 5 km resolution and assigned to both occurrence and background sites. A MaxEnt model^85^ was implemented using the R package dismo (v.1.3.14)^86^ to build the species distribution models, with modern climate data and plant occurrence/pseudo absence information used for model training. Parameter tuning was conducted using the ENMevaluate function in the ENMeval (2.0.5.2) package^87^, which tests regularisation multiplier values ranging from 1.0 to 5.0 in increments of 1 and six different feature combinations (L, LQ, H, LQH, LQHP, LQHPT; where L = linear, Q = quadratic, H = hinge, P = product, and T = threshold) with 5-fold cross-validation, with the best result selected based on the lowest Akaike information criterion (AIC^88^) value.

Model performance was evaluated using the Boyce index, calculated with the Boyce() function from the modEvA (3.45) R package^89,90^. This index compares model predictions with presence/background data. Most ASVs showed high positive Boyce Index values (Supplementary Fig. 4), indicating that species occurrences were more frequent than expected by chance in areas of higher predicted suitability, thus reflecting strong concordance between model predictions and observed occurrences. To predict past species distributions, downscaled reconstructions of mean annual and mean July temperatures were used as inputs to the MaxEnt models using the ‘predict’ function. The prediction was repeated five times for each ASV and time slice, and the mean suitability values were calculated. Suitability thresholds were applied using the “10th percentile training presence” criterion, with suitability values below the threshold converted to absence (0). This process produced occurrence suitability values for each ASV in each 5 km × 5 km grid cell across the study region at 1000-year intervals throughout the past.

### Environmentally constrained null models for inferring plant interactions

SedaDNA data obtained from resampling in each lake and time slice were used to determine species presence/absence information. The procedure follows the protocol of D’Amen et al.^27^, which disentangles species interactions from environmental filters and dispersal limitation as drivers of species co-occurrence (Supplementary Fig. 3). For each species in the 5 km × 5 km grid cell that encompasses the lake, the suitability predicted by the species distribution model (SDM) was used as a proxy for the probability of taxon presence. As the species distribution model was built based on the mean annual temperature and the mean July temperature, the SDM output reflected temperature-driven occurrence patterns; the sedaDNA data were assumed as the actual occurrence of species.

The strength of association between each species a and b was quantified using the C-score index, calculated as:

3 $${Cscoreab}=({Ra}-S)\times ({Rb}-S)/({Ra}\times {Rb})$$

where Ra is the number of sites where species a occurs, Rb is the number of sites where species b occurs, and S is the number of sites in which a and b co-occur. An index value of 0.0 indicates that the species pair is maximally aggregated, with completely nested occurrences. While a value of 1.0 indicates maximal segregation, with the two species never co-occurring. The C-score was calculated using the occurrences of species a and b derived from the sedaDNA data. For each time slice, the sedaDNA data were arranged so that rows represent sampling sites and columns represent species. To generate a null plant community for the same time slice, the species occurrence frequencies (column sums) from the sedaDNA data were preserved, while species richness per plot (row totals) was allowed to vary randomly and equiprobably. Species occurrence sites are determined by the suitability predicted by the SDM, with sites of higher suitability having a greater probability of species occurrence, and vice versa. The generation of the null plant community was repeated 100 times.

The Empirical Bayes method was used to identify statistically significant species pairs. For both the sedaDNA data and the null plant community, each pairwise C-score was assigned to one of 22 evenly spaced bins spanning the interval from 0 to 1. The average number of species pairs with different scores in each bin was then calculated from the null communities, representing the null expectation of the C-score for species pairs in that bin. Only bins in which the number of species pairs observed in the sedaDNA data exceeded the null expectation were retained. For each bin, the mean C-score value of the simulated null distribution was calculated, species pairs from the sedaDNA were ordered by their scores within each bin, and those with scores greater than the mean of the simulated distribution (Bayes M criterion) were retained. The set of significant species pairs was further refined by retaining only those that were statistically significant at the individual-test level (simple confidence-limit criterion). For each species pair, if the C-score derived from the sedaDNA data is significantly higher than that from the environmentally constrained null models, the species are more segregated than expected based on environmental factors. Conversely, if the C-score is lower, the species are more aggregated than expected.

To ensure that species segregation is not driven by the dispersal limitation (e.g., species a and species b being segregated simply due to non-overlapping distribution ranges), the distribution range of each species pair was compared. The Extent of Occurrence (EOO) method^91^ was used to calculate the distribution range, which defines the minimum convex polygon encompassing the occupied plots. A MANOVA analysis was then conducted to test whether the spatial centroids of the two species’ occurrences differed significantly. If the spatial centroids do not differ, the species pair association can be attributed to positive or negative interactions. If the centroids differ, the association may be due to either interactions or dispersal limitations. In this study, only species pairs with non-differing spatial centroids were considered to represent species interactions.

For each timeslice and each resampling of the sedaDNA data, the environmental constrained null model was applied to identify species pairs driven by biotic interactions. An unweighted plant interaction network was then constructed from these species pairs using the igraph (2.3.3) R package^92–94^. In the network, aggregated species pairs were assigned a correlation value of 1 (positive interaction), segregated pairs a correlation value of –1 (negative interaction), and all other pairs a correlation value of 0 (no interaction). The final plant interaction network was visualised with the ggraph (2.2.2) R package^95^, and for one randomly selected resampling, two representative networks are shown in Fig. 2a.

Boxplots of the proportions of positive and negative interactions across different growth-form taxa, temporal trends in the relative proportions of positive and negative interactions, and temporal variation in the number of taxa of different growth forms engaged in positive and negative interactions were calculated across all 100 resamplings and visualised with the ggplot2 (3.5.2) R package^74^ (Fig. 2b–e).

To assess the reliability of the plant interaction result, the plant interaction pairs were compared with the Globi plant interaction database^96^ at both the species and genus levels, to determine the proportion of plant interaction pairs that can be found in the database. Additionally, an equal number of interaction pairs were randomly assigned to taxa that were recorded in the sedaDNA dataset but not listed as plant interaction pairs. This random process was repeated 100 times. The overlap between the randomly assigned interaction pairs was compared with the database as well to get the proportion. Finally, the plant interaction overlap rate from the sedaDNA was compared with the rate from the 100-fold randomisations, and the as.randtest() function in the ade4 (1.7-23) R package^97^ used to test if the overlap rate of the plant interactions is significantly greater than the random rate.

### Plant functional traits

Three functional traits were checked in this study, which are plant growth-form, height, and maximum root length. Growth-form information for each species was primarily obtained from the TRY 5.0 database^43^; for species lacking database entries, growth-forms were verified manually. For each ASV, the growth-form was assigned based on the most frequent growth-form among the associated species. To account for large differences in read count magnitudes among plant growth-forms, read counts for each ASV were square-root transformed. In each time slice, the relative abundance of each ASV was calculated by dividing its transformed count by the total transformed counts across all ASVs. The abundance of each growth-form was then obtained by summing the relative abundances of all ASVs assigned to that growth-form. Plant height and root length data were obtained from multiple sources, including the Tundra Trait Team (TTT) database^47^, the TRY 5.0 database^43^, and the Global Root Trait (GRooT) database^98^, with additional height data for several species manually retrieved. For ASVs lacking functional trait information, they were not included in subsequent analyses. Functional traits were assigned to each ASV using the mean values of all species corresponding to that ASV. To assess temporal changes of plant functional traits, we weighted each ASV’s functional traits by its relative proportion of counts. The weighted values were then summed to derive community-level functional traits.

### Structural equation model

To detect potential autocorrelation, the correlation between woody plant abundance and plant functional traits was assessed using the “Pearson” method and a “two-sided” alternative with the cor.test function from the stats (4.3.1) package in R^99^. Based on the correlation analysis, we constructed a structural equation model (SEM) according to a conceptual framework, incorporating community mean plant height, root length, and mean annual temperature as observed variables. Negative plant interaction was specified as the response variable. All variables were derived from 100 resampling iterations, each containing 12 time slices. This yielded a total of 1200 observations for analysis (*n* = 100 × 12). The ‘Trait’ was modelled as a latent variable—an unobserved factor inferred from two observed indicators: height and root length. As latent variables lack inherent measurement scales, the factor loading of height on the latent construct was fixed at 1 to define the scale of the latent dimension. In the theoretical framework, traits were assumed to be influenced by temperature and, in turn, to mediate negative interactions among plants. To evaluate this conceptual framework, we specified a structural equation model (SEM) using the sem() function from the lavaan (0.7-2) package^100,101^ in R and assessed its overall goodness-of-fit.

All statistical analyses were conducted in R (version 4.3.1)^99^. Additional R packages used included: data.table (1.17.8)^102^, dplyr (1.1.4)^103^, tidyr (1.3.1)^104^, tidyverse (2.0.0)^105^, betapart (1.6.1)^106^, ggpubr (0.6.1)^107^, zoo (1.8-14)^108^, stars (0.6-8)^109^, sf (1.0-21)^110^, terra (1.8-15)^111^, ncdf4 (1.2)^112^, rJava (1.0-11)^113^, raster (3.6-31)^114^, FNN (1.1.4.1)^115^, mgcv (1.9-3)^116^, rnaturalearth (1.1.0)^117^, spatstat (3.4-0)^118^, glue (1.8.0)^119^, stringr (1.5.1)^120^, foreach (1.5.2)^121^, Hmisc (5.2-3)^122^, ggrepel (0.9.6)^123^, textshape (1.7.5)^124^, ggcorrplot (0.1.4.1)^125^, and semPlot (1.1.7)^126^.

### Reporting summary

Further information on research design is available in the Nature Portfolio Reporting Summary linked to this article.

## Supplementary information

Supplementary Information

Reporting Summary

Transparent Peer Review file

## Source data

Source Data

## Data Availability

The raw sedaDNA sequence data have been deposited in the European Nucleotide Archive (ENA) at EMBL-EBI under accession numbers PRJEB76237 and PRJEB70434. The data generated in this study and used in the analyses, including filtered plant distribution data from GBIF, palaeoclimate downscaling results from MPI-ESM1.2, and plant trait data from the Tundra Trait Team (TTT) database, the TRY 5.0 database, and the Global Root Trait (GRooT) database, are available at Zenodo (https://doi.org/10.5281/zenodo.21453331). The source data underlying the figures are provided as Source Data with this paper. Source data are provided with this paper.

## Code availability

The R scripts used for data processing and figure generation are available via Zenodo (https://doi.org/10.5281/zenodo.21453331).
