# Supplementary material for: Woody plant encroachment enhanced negative interactions in Arctic plant communities over the last 24,000 years
Source: Nat Commun. 2026 Sep 22;17:10083. doi: 10.1038/s41467-026-77801-9 (PMC13597523; doi:10.1038/s41467-026-77801-9)
Supplement: Supplementary file 1 [file 41467_2026_77801_MOESM1_ESM.pdf]

Supporting Information for

Woody plant encroachment enhanced negative interactions in Arctic plant communities over the last 24,000 years

Ying Liu[1,2], Weihan Jia[1,2], Sisi Liu[1], Simeon Lisovski[1], Kathleen R. Stoof-Leichsenring[1], Luca Zsofia Farkas1, Ulrike Herzschuh*[1,2,3]

1 Alfred Wegener Institute Helmholtz Centre for Polar and Marine Research, Polar Terrestrial Environmental Systems, 14473 Potsdam, Germany

2 Institute of Environmental Science and Geography, University of Potsdam, 14476 Potsdam, Germany

3 Institute of Biochemistry and Biology, University of Potsdam, 14476 Potsdam, Germany

***Correspondence**

Ulrike Herzschuh: Ulrike.Herzschuh@awi.de

This file includes:

Supplementary Figure 1 to Supplementary Figure 16.

Supplementary Figures

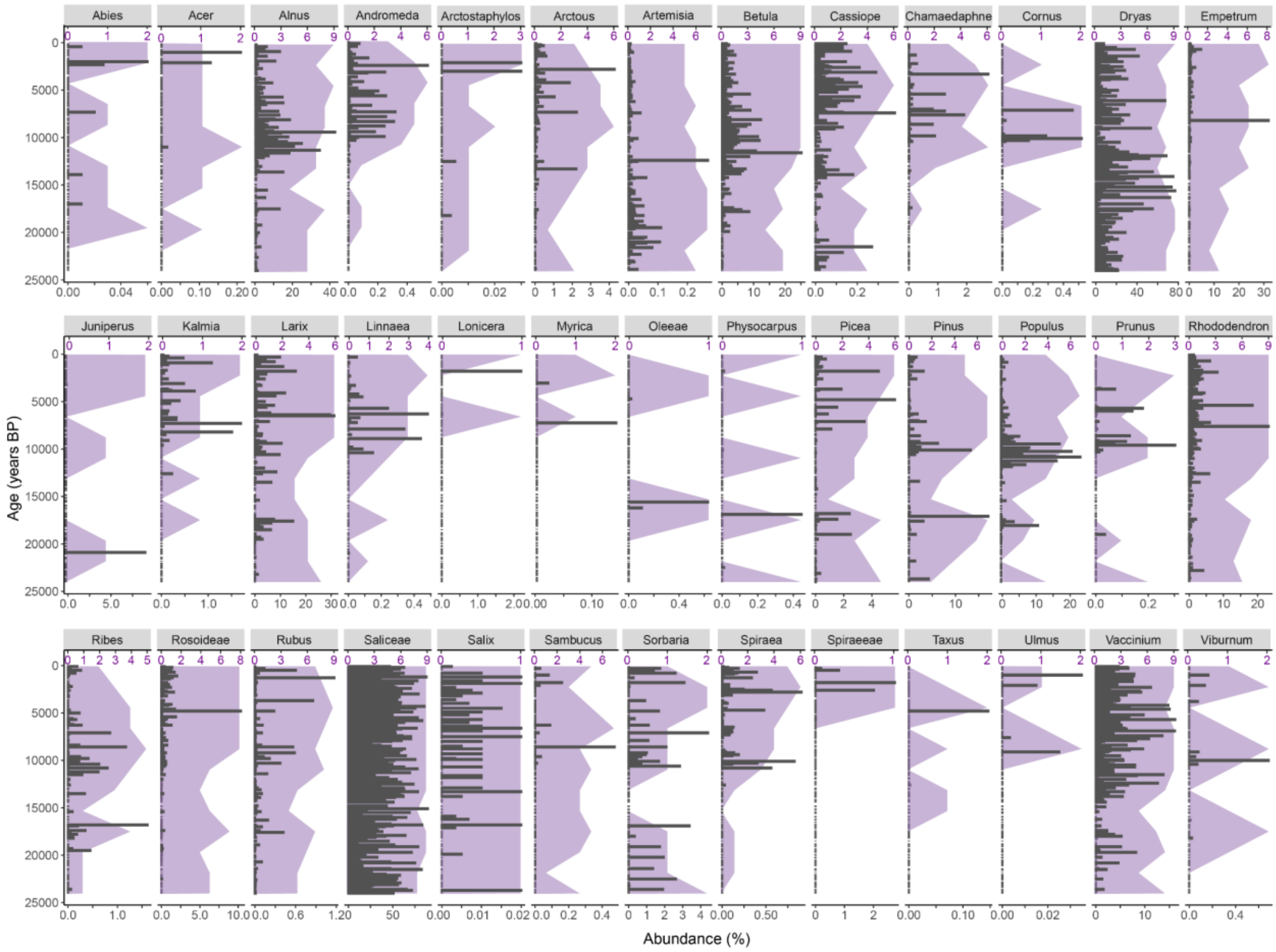

Supplementary Figure 1. Temporal changes in the abundance and occurrence of woody taxa. Abundance is indicated by the proportion of rarefied sedaDNA read counts for each taxon. Black bars show the abundance of each taxon (bottom black x-axis), while the purple polygon indicates the number of lakes where each taxon occurs (top purple x-axis).

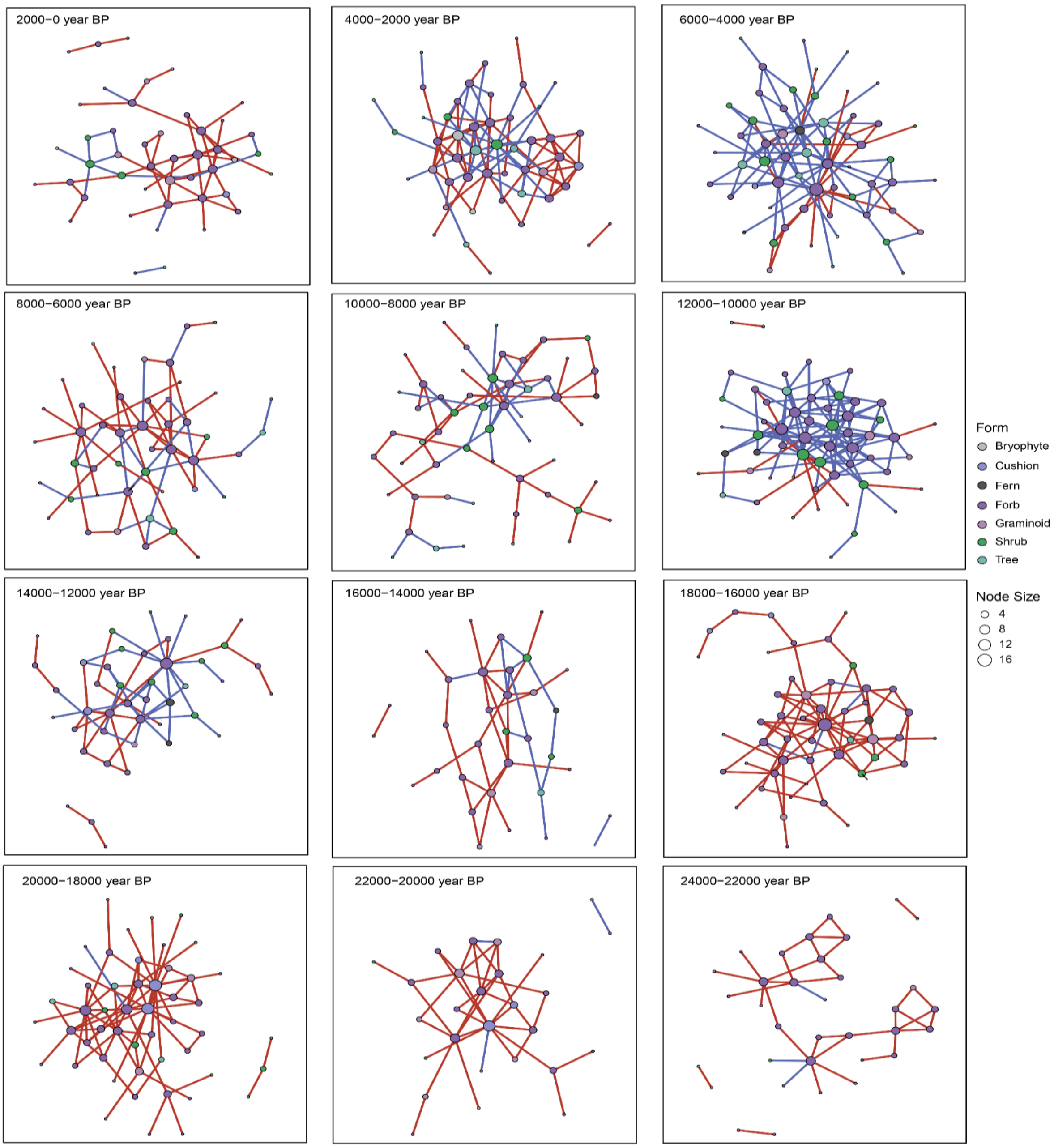

Supplementary Figure 2. Temporal dynamics of the plant interaction network across each 2000-year time slice. Nodes represent taxa, with different growth forms indicated by colour. Node size corresponds to the number of connections (i.e., node degree). The plot shows a representative network randomly selected from 100 randomisation runs.

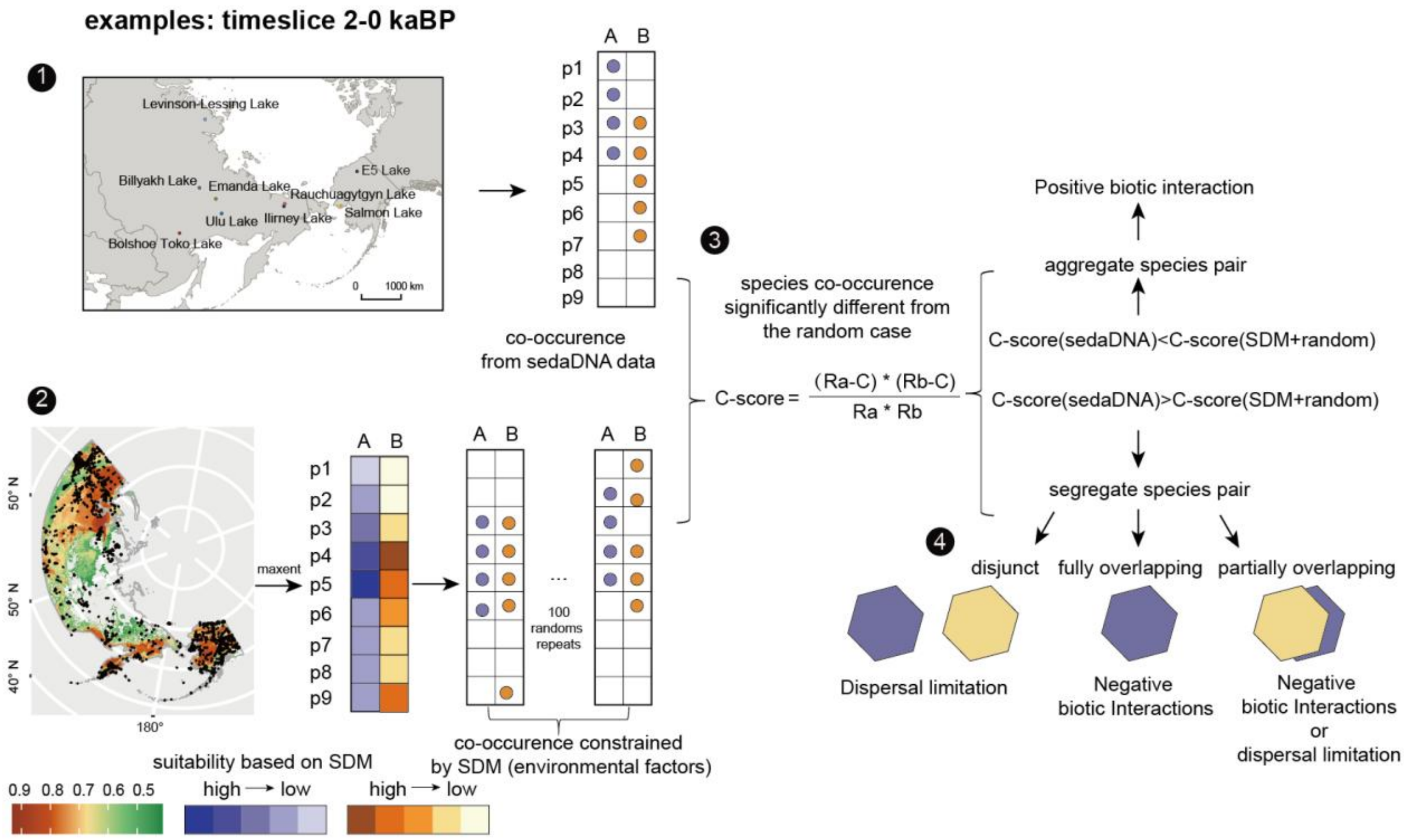

Supplementary Figure 3. Schematic diagram illustrating the disentanglement of plant interaction effects, mean annual temperature/mean July temperature effects, and dispersal limitation using an environmentally constrained null model.

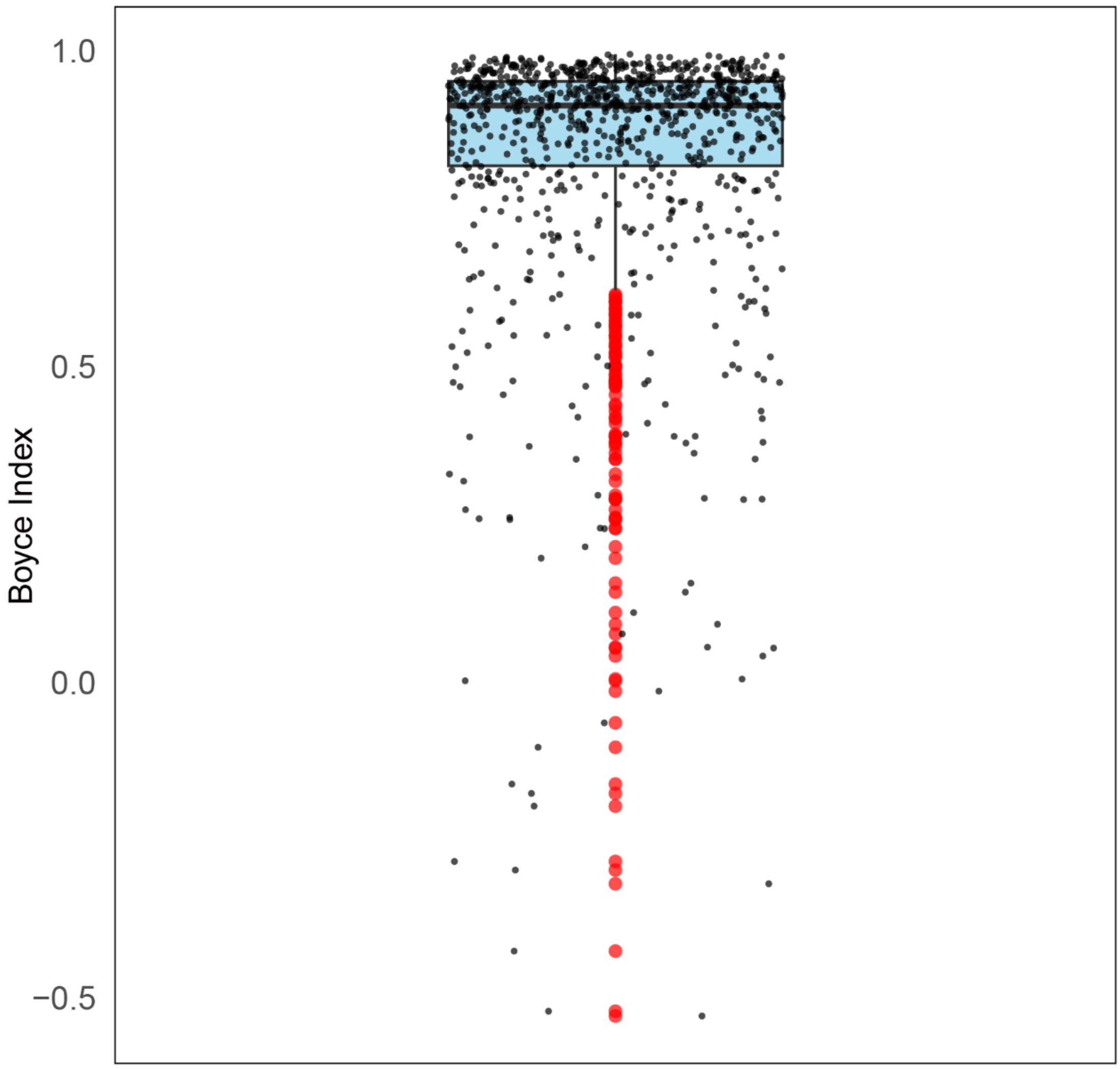

Supplementary Figure 4. Boxplot of the Boyce Index, which was used to evaluate ASV presence results in comparison to predictions from the Species Distribution Model (SDM). Black points represent individual Boyce Index values, and red points indicate outliers. The Boyce Index ranges from -1 to 1: positive values indicate that species occurrences are more frequent than expected by chance in areas with higher predicted suitability, reflecting a strong agreement between model predictions and observed occurrences. Values near 0 suggest predictions are no better than random, while negative values imply species tend to occur in areas with lower predicted suitability, indicating a mismatch between predictions and actual occurrences.

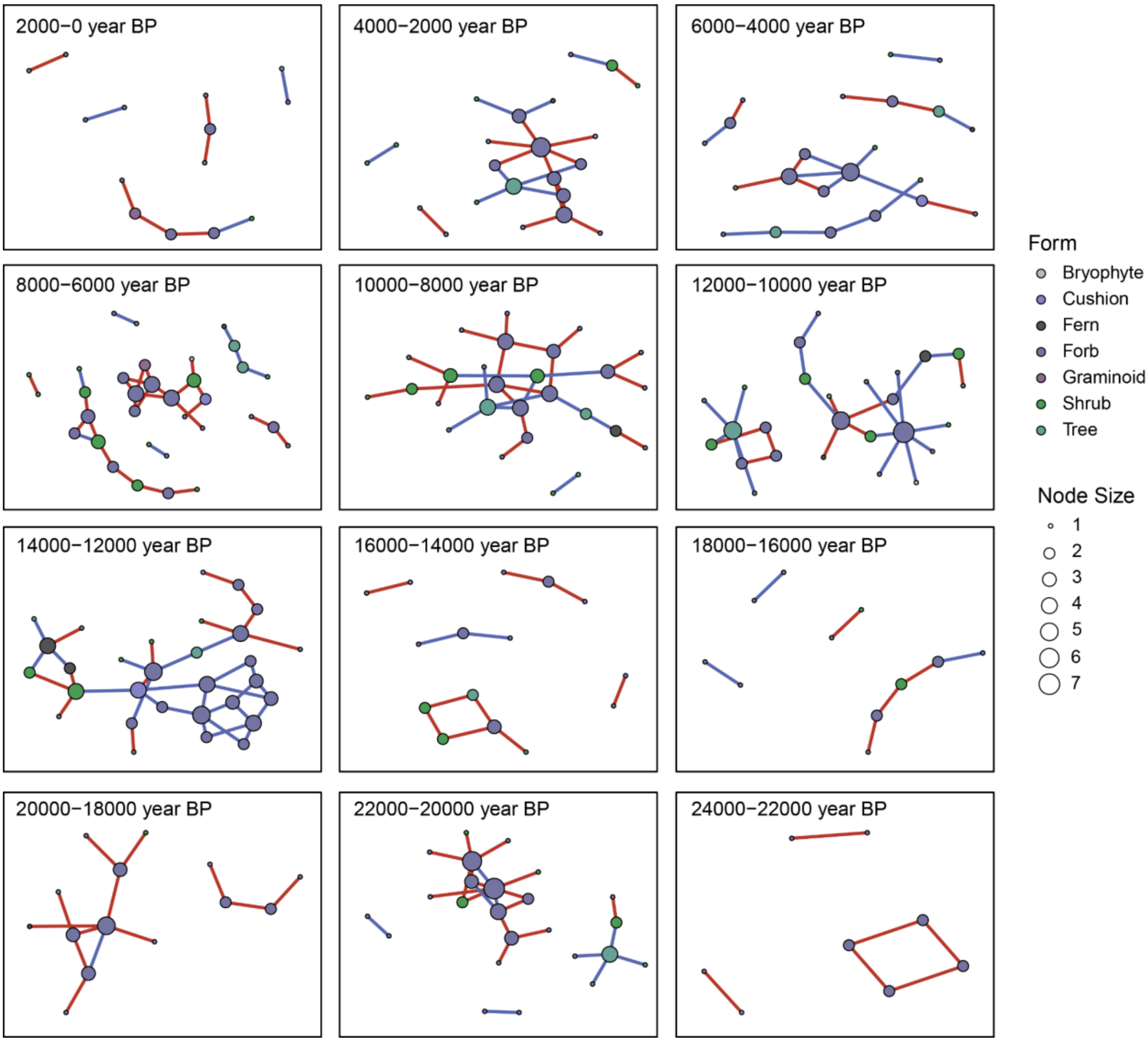

Supplementary Figure 5. Temporal dynamics of the plant interaction network across 2000-year time slices, with one sample each lake each timeslice and rarefied to 2,000 reads to standardise the samples and read numbers within time bins. Nodes represent taxa, with different growth-forms indicated by colour. Edges between nodes represent interactions between taxa, with red edges indicating positive interactions and blue edges indicating negative interactions. Node size corresponds to the number of connections (i.e., node degree). The plot shows a representative network randomly selected from 100 randomisation runs.

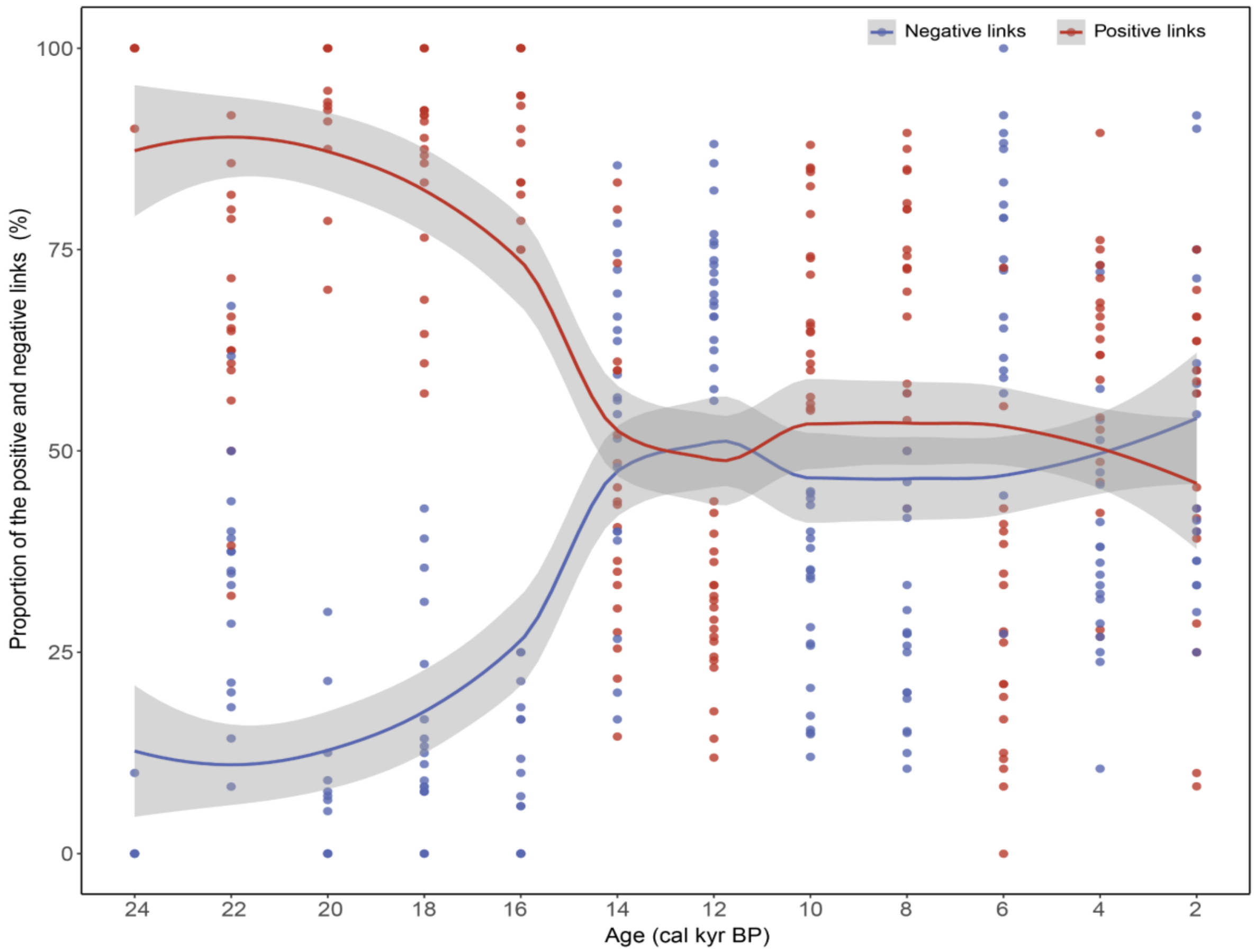

Supplementary Figure 6. Temporal trends in the relative proportion of positive and negative interactions, with one sample each lake each timeslice and rarefied to 2,000 reads to standardise the samples and read numbers within time bins. Points represent the proportion of positive and negative interactions at each time slice across 100 resampling iterations. LOESS-fitted trends are shown by lines, with shaded areas representing 95% confidence interval.

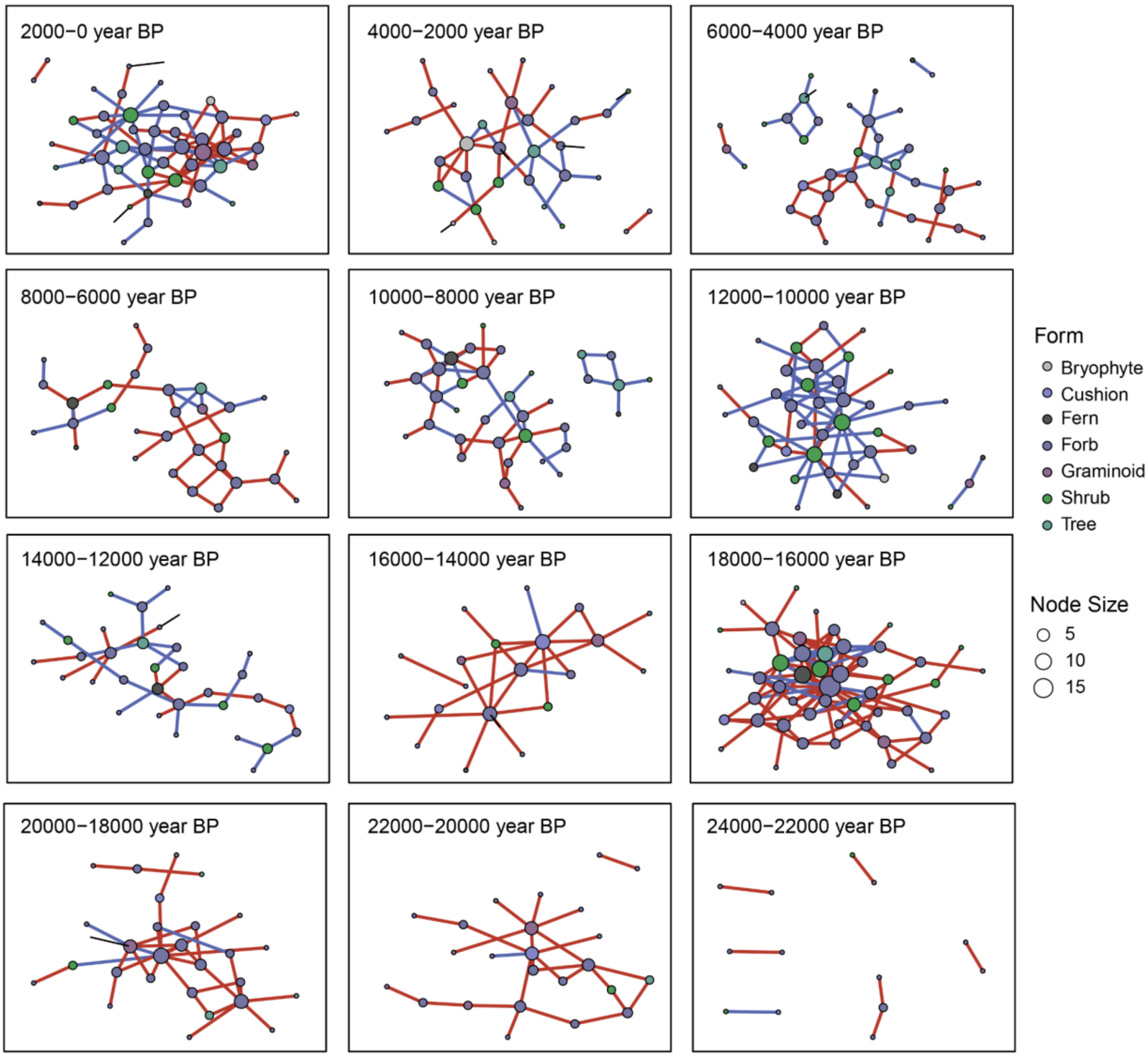

Supplementary Figure 7. Temporal dynamics of the plant interaction network across 2000-year time slices, with each lake rarefied to 10,000 reads each time slice, and one lake site randomly excluded from the analysis. Nodes represent taxa, with different growth-forms indicated by colour. Edges between nodes represent interactions between taxa, with red edges indicating positive interactions and blue edges indicating negative interactions. Node size corresponds to the number of connections (i.e., node degree). The plot shows a representative network randomly selected from 100 randomisation runs.

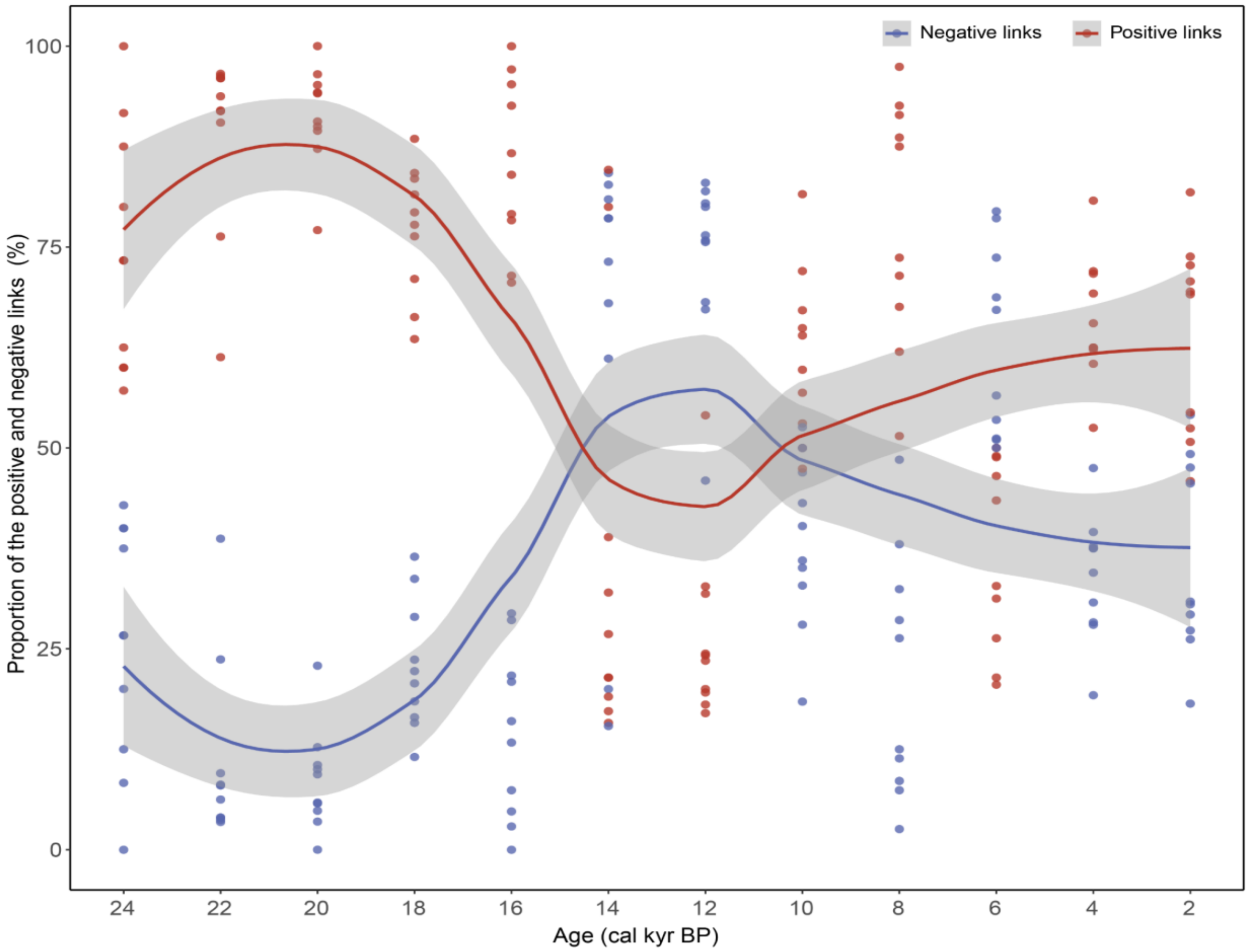

Supplementary Figure 8. Temporal trends in the relative proportion of positive and negative interactions, with each lake rarefied to 10,000 reads each time slice, and one lake site randomly excluded from the analysis. Points represent the proportion of positive and negative interactions at each time slice across 100 resampling iterations. LOESS-fitted trends are shown by lines, with shaded areas representing 95% confidence interval.

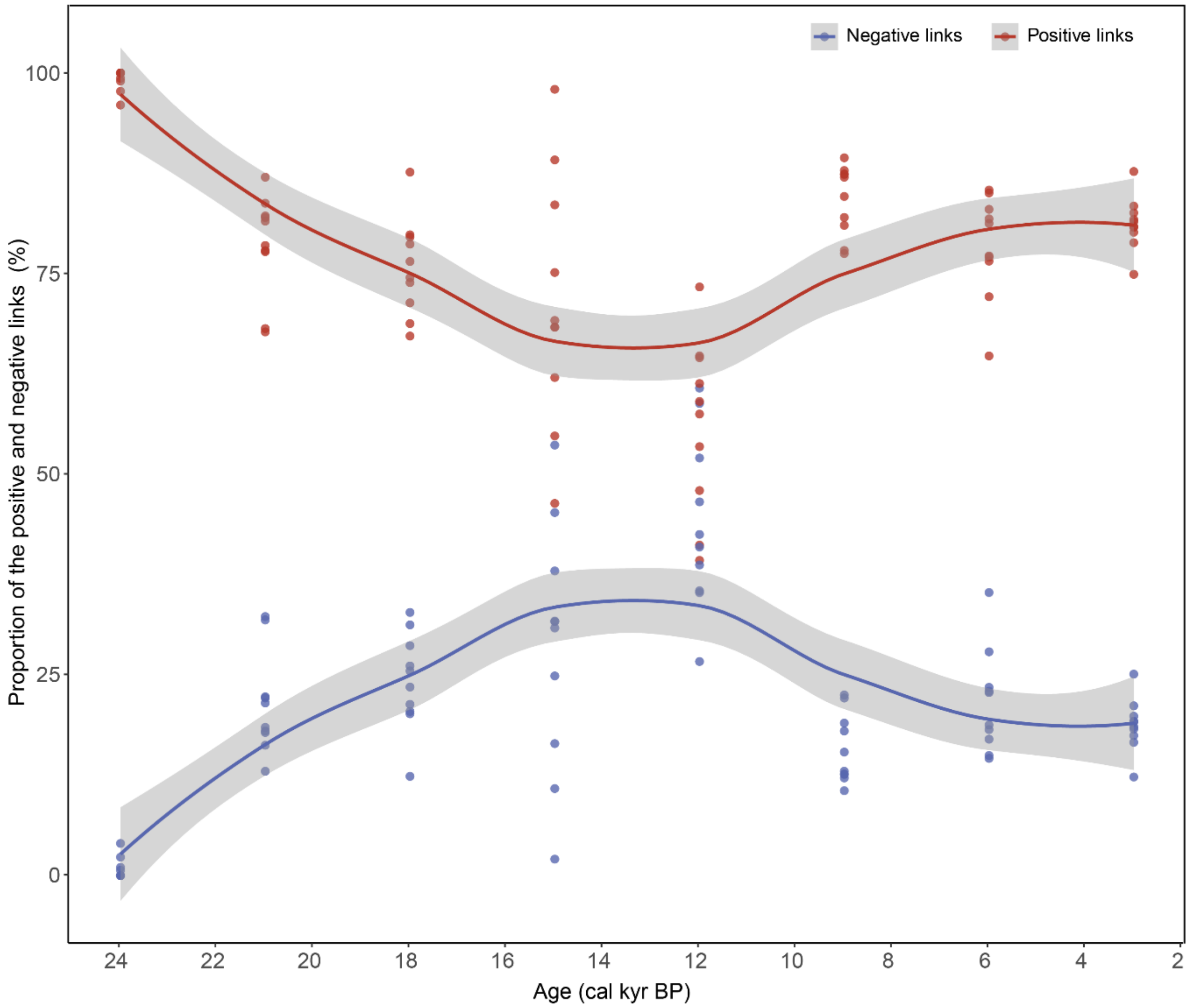

Supplementary Figure 9. Temporal trends in the relative proportion of positive and negative interactions, with a 3,000-year timeslice. Points represent the proportion of positive and negative interactions at each time slice across 100 resampling iterations. LOESS-fitted trends are shown by lines, with shaded areas representing 95% confidence interval.

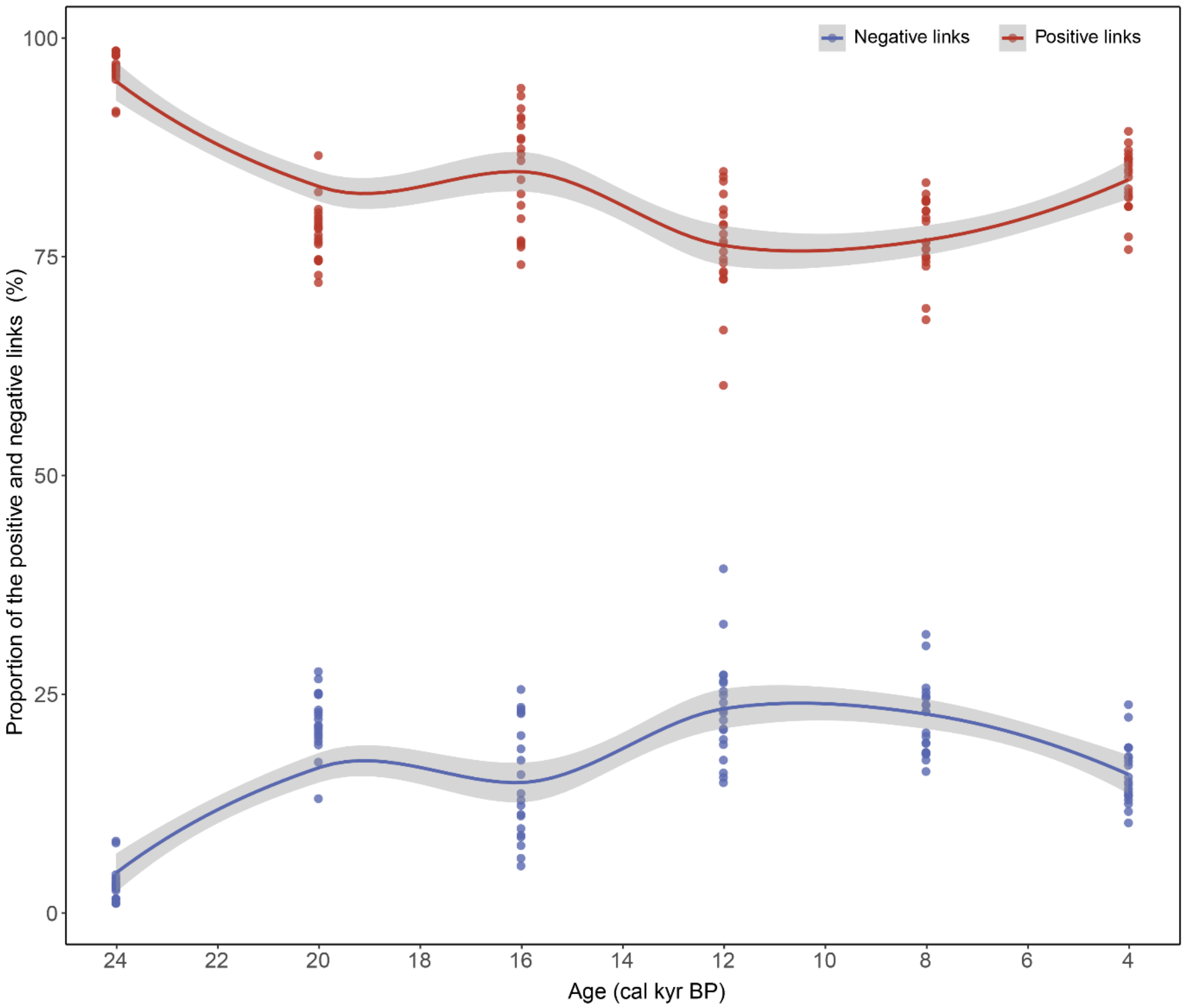

Supplementary Figure 10. Temporal trends in the relative proportion of positive and negative interactions, with a 4,000-year timeslice. Points represent the proportion of positive and negative interactions at each time slice across 100 resampling iterations. LOESS-fitted trends are shown by lines, with shaded areas representing 95% confidence interval.

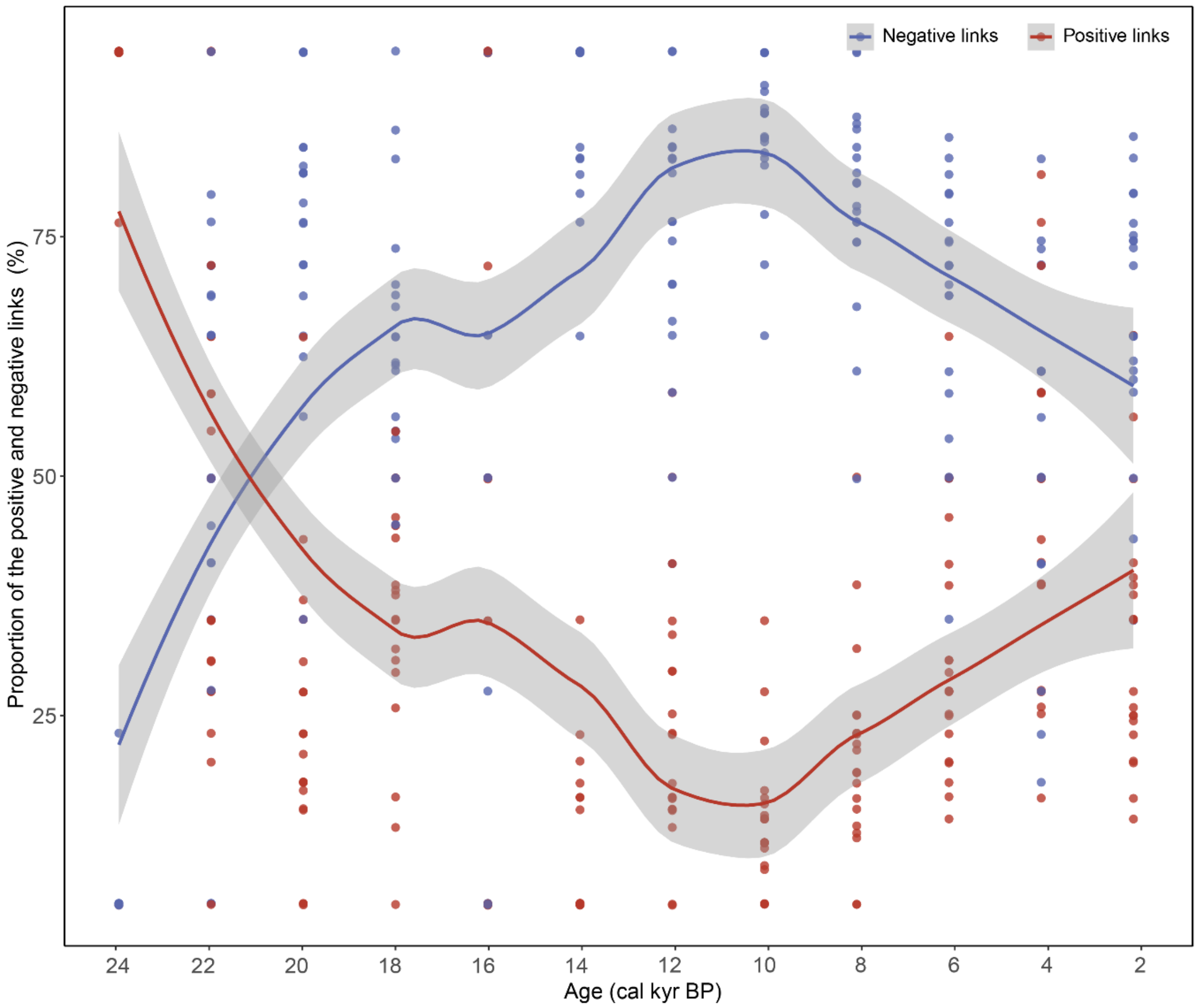

Supplementary Figure 11. Temporal trends in the relative proportion of positive and negative interactions at the family level. Points represent the proportion of positive and negative interactions at each time slice across 100 resampling iterations. LOESS-fitted trends are shown by lines, with shaded areas representing 95% confidence interval.

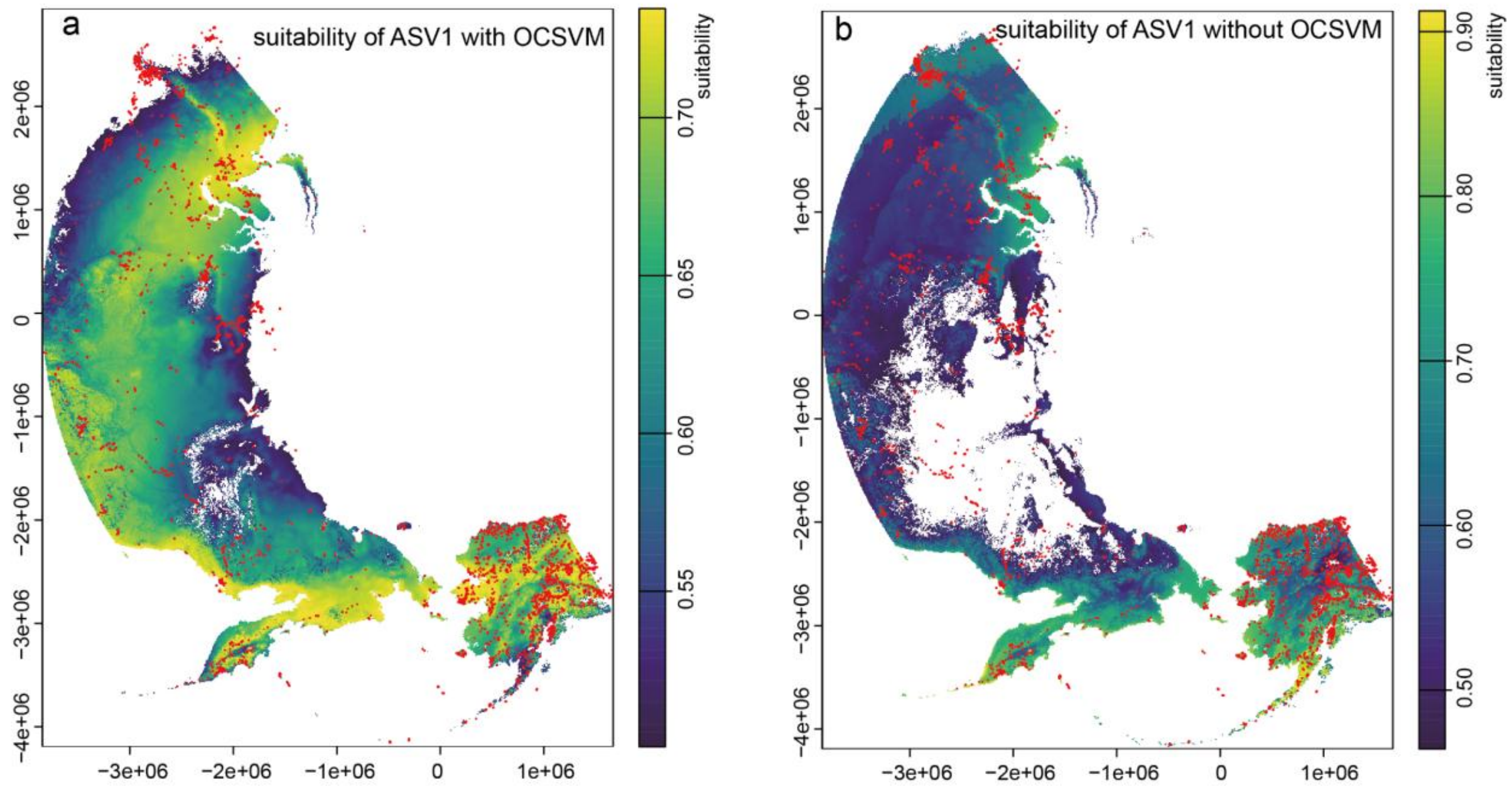

Supplementary Figure 12. Predicted habitat suitability (colour scale) from species distribution models and observed taxon occurrences (red dots). (a) Results with background selection using a one-class support vector machine (OCSVM). (b) Results without OCSVM-based background selection.

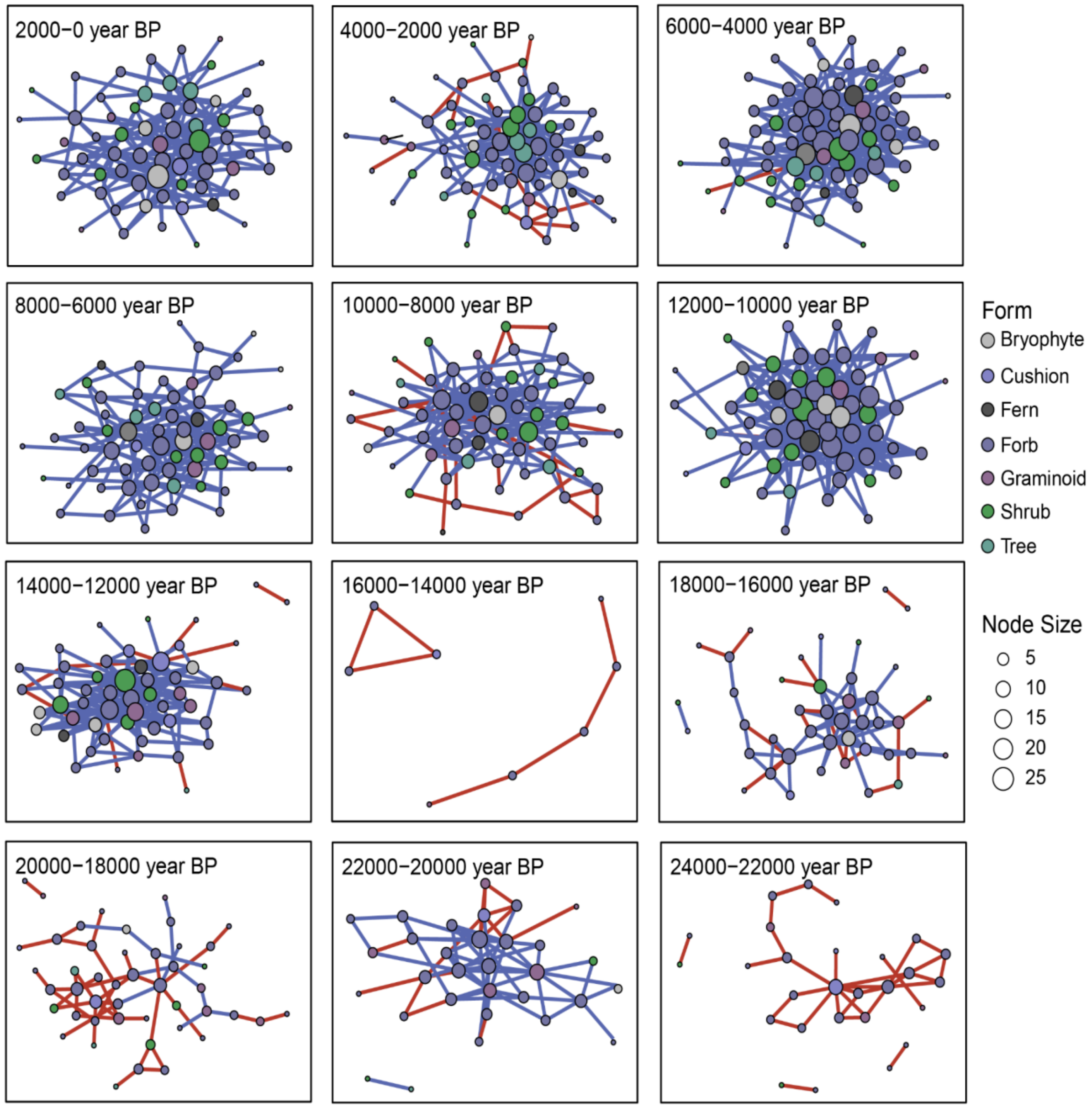

Supplementary Figure 13. Temporal dynamics of the plant interaction network across 2000-year time slices, using background point selection without the one-class support vector machine (OCSVM). Nodes represent taxa, with colours indicating different growth forms. Edges between nodes represent interactions among taxa, with red edges indicating positive interactions and blue edges indicating negative interactions. Node size corresponds to the number of connections (i.e., node degree). The plot shows a representative network randomly selected from 100 randomisation runs.

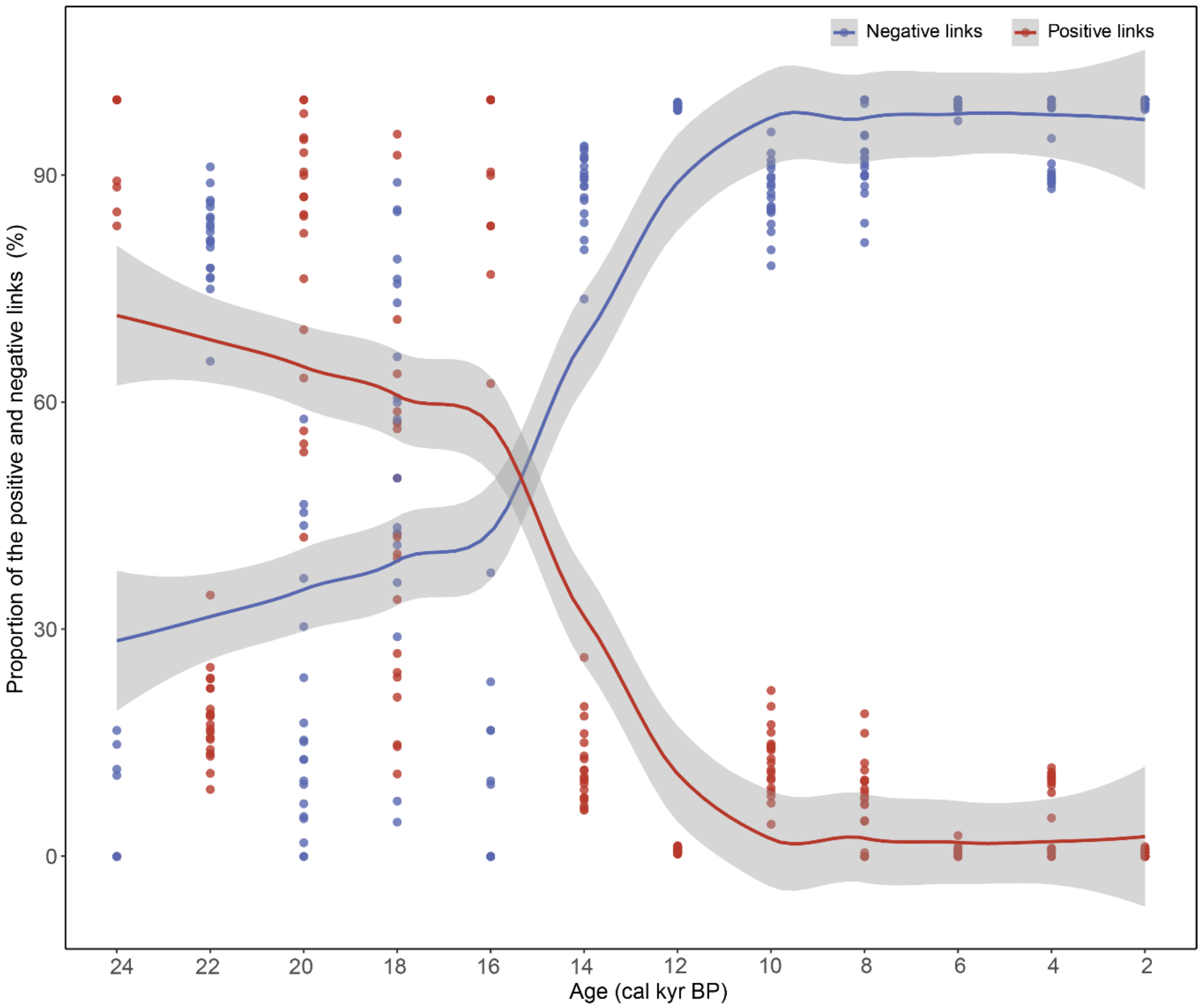

Supplementary Figure 14. Temporal trends in the relative proportion of positive and negative interactions, using background point selection without the one-class support vector machine (OCSVM). Points represent the proportion of positive and negative interactions at each time slice across 100 resampling iterations. LOESS-fitted trends are shown by lines, with shaded areas representing 95% confidence interval.

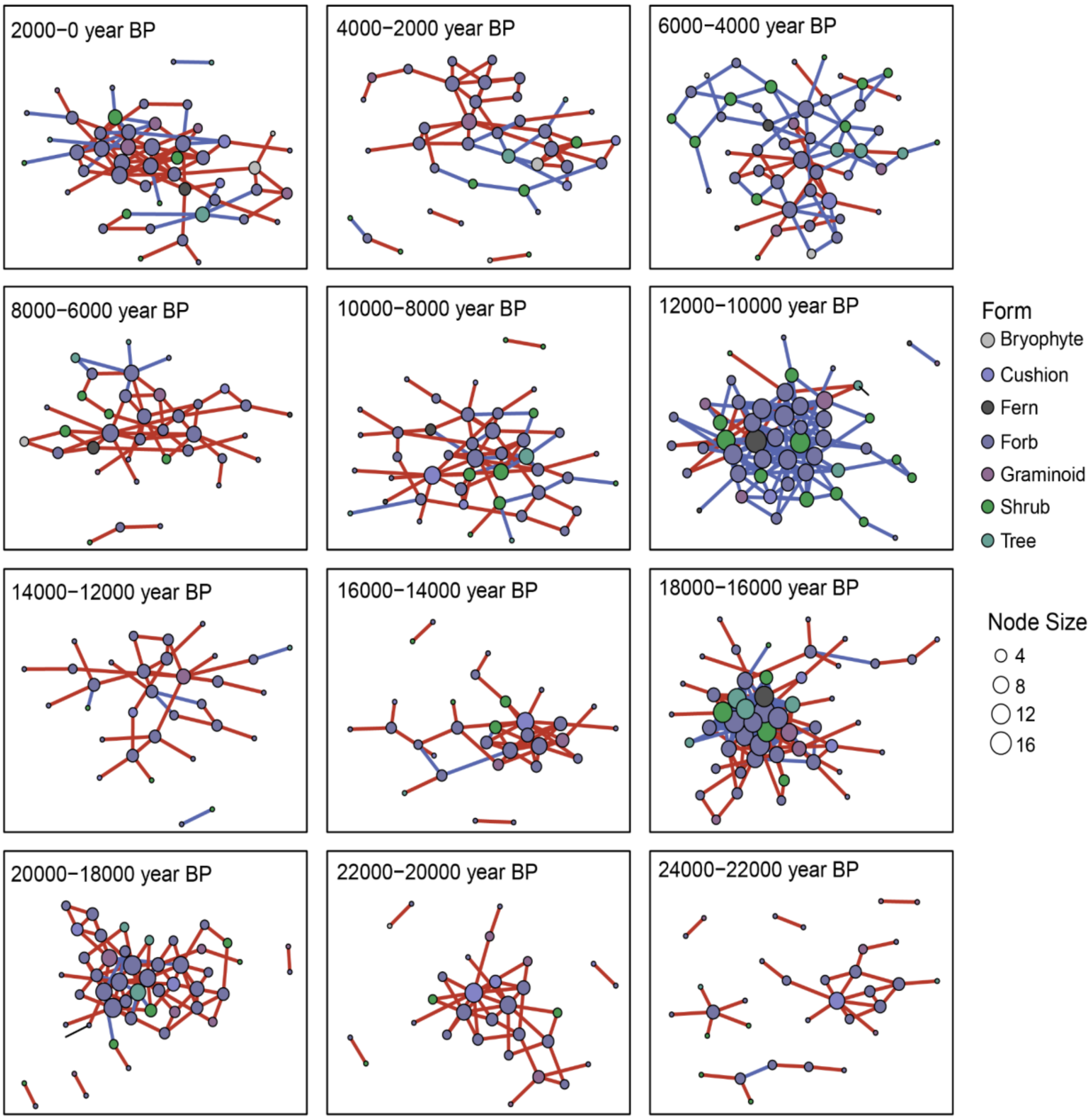

Supplementary Figure 15. Temporal dynamics of the plant interaction network across 2000-year time slices, using background point selection with the one-class support vector machine (OCSVM), with ASV exhibiting outlier Boyce index values excluded. Nodes represent taxa, with colours indicating different growth forms. Edges between nodes represent interactions among taxa, with red edges indicating positive interactions and blue edges indicating negative interactions. Node size corresponds to the number of connections (i.e., node degree). The plot shows a representative network randomly selected from 100 randomisation runs.

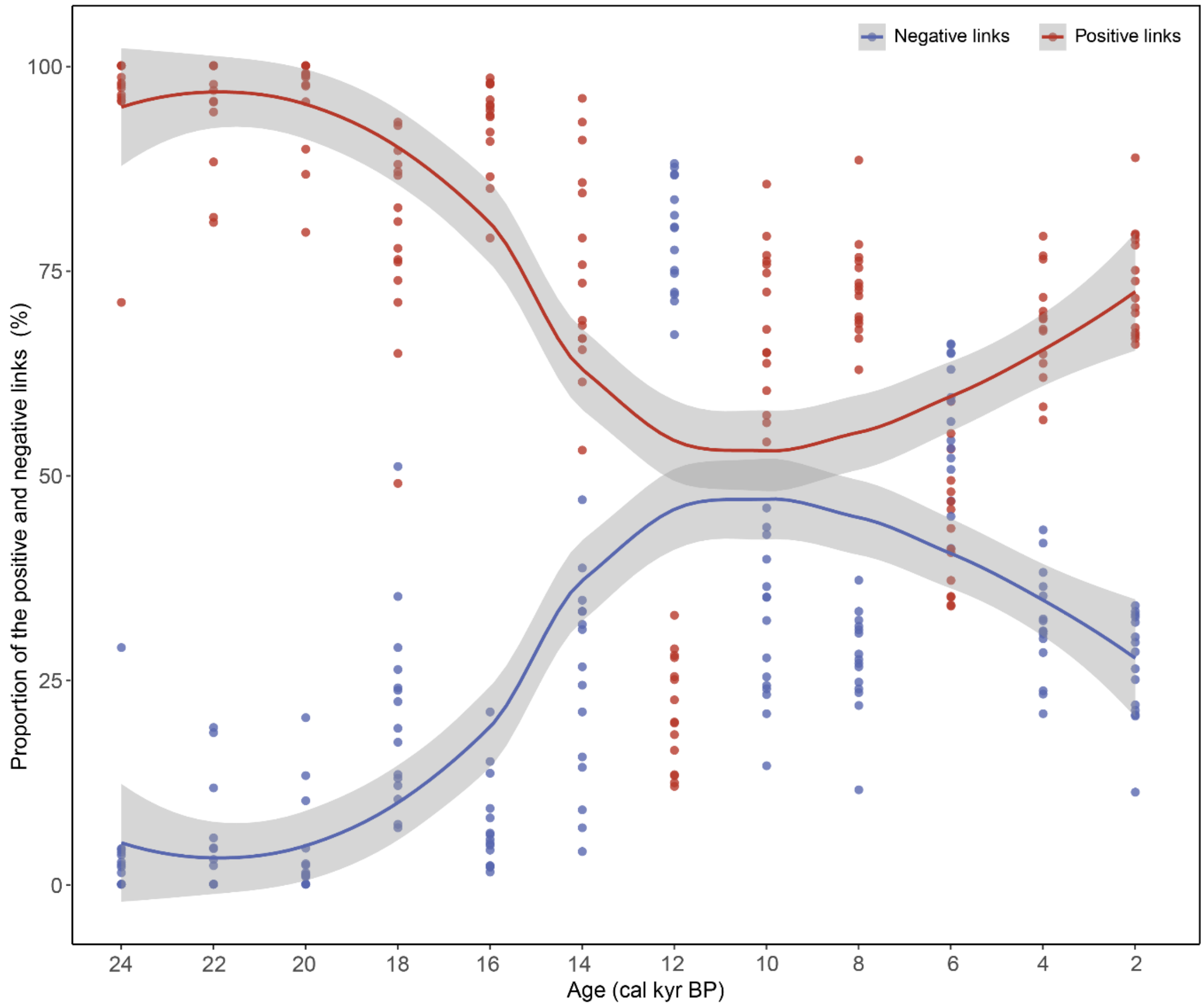

Supplementary Figure 16. Temporal trends in the relative proportion of positive and negative interactions, using background point selection with the one-class support vector machine (OCSVM), with ASV exhibiting outlier Boyce index values excluded. Points represent the proportion of positive and negative interactions at each time slice across 100 resampling iterations. LOESS-fitted trends are shown by lines, with shaded areas representing 95% confidence interval.
