# Supplementary material for: Woody plant encroachment enhanced negative interactions in Arctic plant communities over the last 24,000 years
Source: Nat Commun. 2026 Sep 22;17:10083. doi: 10.1038/s41467-026-77801-9 (PMC13597523; doi:10.1038/s41467-026-77801-9)
Supplement: Supplementary file 2 [file 41467_2026_77801_MOESM2_ESM.pdf]

nature portfolio

Corresponding author(s): Ulrike Herzschuh

Last updated by author(s): Jul 20, 2026

Reporting Summary

Nature Portfolio wishes to improve the reproducibility of the work that we publish. This form provides structure for consistency and transparency in reporting. For further information on Nature Portfolio policies, see our Editorial Policies and the Editorial Policy Checklist.

Statistics

For all statistical analyses, confirm that the following items are present in the figure legend, table legend, main text, or Methods section.

n/a	Confirmed	
☐	☒	The exact sample size (*n*) for each experimental group/condition, given as a discrete number and unit of measurement
☐	☒	A statement on whether measurements were taken from distinct samples or whether the same sample was measured repeatedly
☐	☒	The statistical test(s) used AND whether they are one- or two-sided<br>*Only common tests should be described solely by name; describe more complex techniques in the Methods section.*
☐	☒	A description of all covariates tested
☐	☒	A description of any assumptions or corrections, such as tests of normality and adjustment for multiple comparisons
☐	☒	A full description of the statistical parameters including central tendency (e.g. means) or other basic estimates (e.g. regression coefficient) AND variation (e.g. standard deviation) or associated estimates of uncertainty (e.g. confidence intervals)
☐	☒	For null hypothesis testing, the test statistic (e.g. *F*, *t*, *r*) with confidence intervals, effect sizes, degrees of freedom and *P* value noted<br>*Give P values as exact values whenever suitable.*
☒	☐	For Bayesian analysis, information on the choice of priors and Markov chain Monte Carlo settings
☒	☐	For hierarchical and complex designs, identification of the appropriate level for tests and full reporting of outcomes
☐	☒	Estimates of effect sizes (e.g. Cohen's *d*, Pearson's *r*), indicating how they were calculated

Our web collection on statistics for biologists contains articles on many of the points above.

Software and code

Policy information about availability of computer code

Data collection	NA
Data analysis	OBITools v3; R (version 4.3.1); ggplot2 (3.5.2) R package); dismo (v.1.3.14) R package; ENMeval (2.0.5.2) R package; igraph (2.1.4) R package; ggraph (2.2.2) R package; ade4 (1.7-23) R package;stats (4.3.1) R package; lavaan (0.6-19) package; modEvA (3.45) R package; parallel (4.3.1) R package; data.table (1.17.8) R package; dplyr (1.1.4) R package; tidyr (1.3.1) R package; tidyverse (2.0.0) R package; betapart (1.6.1) R package; ggpubr (0.6.1) R package; zoo (1.8-14) package; stars (0.6-8) R package; sf (1.0-21) R package; terra (1.8-15) R package; ncdf4 (1.24) R package; rJava (1.0-11) R package; raster (3.6-31) R package; FNN (1.1.4.1) R package; e1071 (1.7-16) R package; mgcv (1.9-3) R package; rnaturalearth (1.1.0) package; spatstat (3.4-0) R package; glue (1.8.0) R package;stringr (1.5.1) R package; foreach (1.5.2) R package; Hmisc (5.2-3) R package; ggrepel (0.9.6) R package; textshape (1.7.5) R package; ggcorrplot (0.1.4.1) R package; semPlot (1.1.7) R package. The R scripts used for data processing and figure generation are available via Zenode [https://doi.org/10.5281/zenodo.21453331].

For manuscripts utilizing custom algorithms or software that are central to the research but not yet described in published literature, software must be made available to editors and reviewers. We strongly encourage code deposition in a community repository (e.g. GitHub). See the Nature Portfolio guidelines for submitting code & software for further information.

Data

Policy information about availability of data

All manuscripts must include a data availability statement. This statement should provide the following information, where applicable:

- Accession codes, unique identifiers, or web links for publicly available datasets
- A description of any restrictions on data availability
- For clinical datasets or third party data, please ensure that the statement adheres to our policy

SedaDNA dataset originally generated by Courtin et al. 2025 and Jia et al. 2024, and Herzschuh et al 2025;
The modern plant distribution data was downloaded from GBIF (https://doi.org/10.15468/dd.y4uq5q);
Palaeoclimate simulations for mean annual temperature and mean July temperature were obtained from the Max Planck Institute Earth System Model (MPI-ESM1.2), covering the last 23 ka BP, with a spatial resolution of approximately 3.75° × 3.75°;
Growth-form information for each species was primarily obtained from the TRY 5.0 database; for species lacking database entries, growth-forms were verified manually;
Plant height and root length data were obtained from multiple sources, including the Tundra Trait Team (TTT) database, the TRY 5.0 database, and the Global Root Trait (GRooT) database, with additional height data for several species manually retrieved;

Research involving human participants, their data, or biological material

Policy information about studies with human participants or human data. See also policy information about sex, gender (identity/presentation), and sexual orientation and race, ethnicity and racism.

Reporting on sex and gender	*Use the terms sex (biological attribute) and gender (shaped by social and cultural circumstances) carefully in order to avoid confusing both terms. Indicate if findings apply to only one sex or gender; describe whether sex and gender were considered in study design; whether sex and/or gender was determined based on self-reporting or assigned and methods used. Provide in the source data disaggregated sex and gender data, where this information has been collected, and if consent has been obtained for sharing of individual-level data; provide overall numbers in this Reporting Summary. Please state if this information has not been collected. Report sex- and gender-based analyses where performed, justify reasons for lack of sex- and gender-based analysis.*
Reporting on race, ethnicity, or other socially relevant groupings	*Please specify the socially constructed or socially relevant categorization variable(s) used in your manuscript and explain why they were used. Please note that such variables should not be used as proxies for other socially constructed/relevant variables (for example, race or ethnicity should not be used as a proxy for socioeconomic status). Provide clear definitions of the relevant terms used, how they were provided (by the participants/respondents, the researchers, or third parties), and the method(s) used to classify people into the different categories (e.g. self-report, census or administrative data, social media data, etc.) Please provide details about how you controlled for confounding variables in your analyses.*
Population characteristics	*Describe the covariate-relevant population characteristics of the human research participants (e.g. age, genotypic information, past and current diagnosis and treatment categories). If you filled out the behavioural & social sciences study design questions and have nothing to add here, write "See above."*
Recruitment	*Describe how participants were recruited. Outline any potential self-selection bias or other biases that may be present and how these are likely to impact results.*
Ethics oversight	*Identify the organization(s) that approved the study protocol.*

Note that full information on the approval of the study protocol must also be provided in the manuscript.

Field-specific reporting

Please select the one below that is the best fit for your research. If you are not sure, read the appropriate sections before making your selection.

☐ Life sciences ☐ Behavioural & social sciences ☒ Ecological, evolutionary & environmental sciences

For a reference copy of the document with all sections, see nature.com/documents/nr-reporting-summary-flat.pdf

Ecological, evolutionary & environmental sciences study design

All studies must disclose on these points even when the disclosure is negative.

Study description	In this study, we reconstruct plant composition and functional trait turnover during post-glacial woody encroachment using sedimentary ancient DNA from nine lakes in the Siberia-Alaska region. Environmentally constrained null models are applied to partition plant interactions from the pure environment driven plant co-occurrence signal. We found that plant interactions shifted from predominantly positive interactions (e.g. nurse-plant facilitation) during the glacial period to negative interactions (e.g. competition) during the Holocene. This shift coincided with a community transition from herbaceous to woody taxa, leading to an increase in average plant height and root length, as evidenced by leveraging plant trait information.

Research sample	In total 369 sedaDNA samples from nine cores in the northeast Siberia and Alaska region were used in this study, which was derived the study of Courtin et al. 2025, Jia et al. 2024, and Herzschuh et al., 2025.
Sampling strategy	For each of the lake sediment cores, the process of subsampling for sedaDNA analysis took place within the climate chamber at the Helmholtz Centre Potsdam-German Research Centre for Geosciences (GFZ), maintained at 4°C. This environment, devoid of any molecular genetic studies, was chosen to prevent potential contamination with modern DNA. Prior to working on the core, all surfaces in the climate chamber underwent thorough cleaning with DNA Exitus PlusTM and demineralized water. Additionally, sampling tools such as knives, scalpels, and their holders were meticulously cleaned before obtaining each sample, following the recommended procedures. To ensure sterility, approximately 3 mm of each sample slice that came into contact with the plastic tube or the thin foil covering the half-core was removed using a sterile scalpel.
Data collection	SedaDNA dataset originally generated by Courtin et al. 2025 and Jia et al. 2024, and Herzschuh et al 2025;<br>The modern plant distribution data was downloaded from GBIF (https://doi.org/10.15468/dd.y4uq5q);<br>Palaeoclimate simulations for mean annual temperature and mean July temperature were obtained from the Max Planck Institute Earth System Model (MPI-ESM1.2), covering the last 23 ka BP, with a spatial resolution of approximately 3.75° × 3.75°;<br>Growth-form information for each species was primarily obtained from the TRY 5.0 database; for species lacking database entries, growth-forms were verified manually;<br>Plant height and root length data were obtained from multiple sources, including the Tundra Trait Team (TTT) database, the TRY 5.0 database, and the Global Root Trait (GRooT) database, with additional height data for several species manually retrieved;
Timing and spatial scale	Our dataset cover the past 24,000 years and the northeast Siberia and Alaska region.
Data exclusions	For each sample, we performed quality checks on the composition signal of the three replicates' Amplicon Sequence Variants (ASVs). Replicates were excluded from the dataset if their total read count fell below 100. Furthermore, if a sample had fewer than three replicates or displayed different plant sequence types compared to the remaining replicates, those specific replicates were also excluded from the dataset.
Reproducibility	The archive lake sediment cores, along with DNA extraction samples and the prepared DNA amplicon-sequencing libraries, are stored at the Alfred Wegener Institute, Potsdam. The data analysis is reproducible, both the utilized data and scripts are available.
Randomization	Resample the read count randomly using the sample() function.
Blinding	The blinding test is not relevant to this study.

Did the study involve field work? ☐ Yes ☒ No

Reporting for specific materials, systems and methods

We require information from authors about some types of materials, experimental systems and methods used in many studies. Here, indicate whether each material, system or method listed is relevant to your study. If you are not sure if a list item applies to your research, read the appropriate section before selecting a response.

Materials & experimental systems

n/a	Involved in the study
☒	☐ Antibodies
☒	☐ Eukaryotic cell lines
☒	☐ Palaeontology and archaeology
☒	☐ Animals and other organisms
☒	☐ Clinical data
☒	☐ Dual use research of concern
☒	☐ Plants

Methods

n/a	Involved in the study
☒	☐ ChIP-seq
☒	☐ Flow cytometry
☒	☐ MRI-based neuroimaging

Plants

Seed stocks	*Report on the source of all seed stocks or other plant material used. If applicable, state the seed stock centre and catalogue number. If plant specimens were collected from the field, describe the collection location, date and sampling procedures.*
Novel plant genotypes	*Describe the methods by which all novel plant genotypes were produced. This includes those generated by transgenic approaches, gene editing, chemical/radiation-based mutagenesis and hybridization. For transgenic lines, describe the transformation method, the number of independent lines analyzed and the generation upon which experiments were performed. For gene-edited lines, describe the editor used, the endogenous sequence targeted for editing, the targeting guide RNA sequence (if applicable) and how the editor was applied.*
Authentication	*Describe any authentication procedures for each seed stock used or novel genotype generated. Describe any experiments used to assess the effect of a mutation and, where applicable, how potential secondary effects (e.g. second site T-DNA insertions, mosiacism, off-target gene editing) were examined.*
