# Supplementary material for: Woody plant encroachment enhanced negative interactions in Arctic plant communities over the last 24,000 years
Source: Nat Commun. 2026 Sep 22;17:10083. doi: 10.1038/s41467-026-77801-9 (PMC13597523; doi:10.1038/s41467-026-77801-9)
Supplement: Supplementary file 3 [file 41467_2026_77801_MOESM3_ESM.pdf]

nature portfolio

Peer Review File

Woody plant encroachment enhanced negative interactions in Arctic plant communities over the last 24,000 years

Corresponding Author: Professor Ulrike Herzschuh

This file contains all reviewer reports in order by version, followed by all author rebuttals in order by version.

Version 0:

Reviewer comments:

Reviewer #1

(Remarks to the Author)
This manuscript is an excellently written reconstruction of plant interactions in the Arctic that is highly relevant for current climatic trends. The authors provide detailed and sound ecological interpretations of their model and expand on this previously published dataset. The authors have also graciously provided well-documented code and datasheets to follow their analysis. I expect their approach and results will be of interest to a large part of the scientific community. I have one major concern that I would like the authors to comment on:

Many of the lakes used in the study have large and ecologically varied catchments (e.g. Lake Illirney's 1200 km2 catchment comprising Larix forest and high alpine environments). sedaDNA data collapses all of these habitats into one datapoint whereas in reality, taxa may not have existed in close proximity to one another. Some discussion is warranted on how covariation of these taxa in ecologically distinct areas could be interpreted as interaction by the environmentally constrained null model.

Additionally:

L427-429: Some elaboration on what "substantial divergence from other samples" constitutes is needed. Are samples being compared against each other or is it just the PCR replicates within a sample? The referenced paper (Courtin et al. 2025) details clustering PCR replicates within a sample, but the phrasing of "PCR samples were excluded if their replicates showed substantial divergence from other samples" implies other samples are being compared.

(Remarks on code availability)

Reviewer #2

(Remarks to the Author)
In the manuscript "Woody plant encroachment enhances negative interactions in 2 Arctic plant communities over the last 24,000 years", the authors use sedaDNA from several Siberian/Alaskan lakes to examine patterns of ecological interactions throughout the late Pleistocene and Holocene, and how these interactions link to the functional characteristics of the communities there.
Overall, I found the manuscript engaging and written beautifully, and I am excited about the results presented. In particular, I was impressed by the environmentally constrained null-model of community assembly. The results presented were clear and defended very well. However, there are few points of clarification I would like addressed about the methods and the interpretation of the results.

First, I had a few notes about subsampling: I am happy to see that the sedaDNA dataset was subsampled to the same base count (20k) for each 2k year time period, and in your code you make sure each lake has 8 time slices, which is great! However, some lakes could still have more layers/distinct time samples within than others within a particular bin, which could lead to inflated estimates of coexistence. Therefore, it might be useful to not only rarefy on the number of base reads, but also to the minimum number of distinct time periods observed within each lake/time bin combination. In addition, how

robust are your results to spatial variation? I.e., if one lake were removed from the analyses, how do the results change?

Second, the abstract suggests that temperature and plant interactions jointly supported the expansion of woody taxa, but the discussion and the SEM suggest the other way around; that the rising temperature and expansion of woody taxa drove increased competition in the Arctic. I worry that these interpretations run the risk of being somewhat circular. I think this research could benefit from restating the hypotheses/results slightly. For example, is the idea that, when temperatures rose, woody plants began expanding, and this expansion was exacerbated by the increase in competition that occurred (in which case, maybe you could find a lag between the arrival of woody taxa and their dominance relating to the proportion of negative interactions)? Or maybe that the negative interactions drove the extirpation/taxon loss of cushion plants (in which case, you could incorporate cushion plant proportion or richness into your SEM)? Further, I think it might be worth it to test the direct influence of temperature on the prevalence of negative links (i.e., through your SEM), to disentangle these effects slightly.

Thank you for giving me an opportunity to read this interesting research! I've organized more minor comments section by section below:

Introduction:
The introduction does an excellent job of introducing the reader to all of the separate aspects of the research!
Line 54: Replace "All the" with either "our" or "current"
Line 68: "While cushion plants provide insights" might be streamlined into "However," or something similar.
Line 85: "…due to polar amplification" maybe cite?
Lines 93-98: I think this section could mostly be removed: instead focus on what you did do (something like "Environmentally-constrained models can ______, providing insight into ____, but these models have not been used in _______"
Lines 102-111: Excellent paragraph! Very clear!
Results:
The title headings are very informative, and I really like that they reflect the last sentence in the introduction.
Line 118: Link to Figure 1 here
Line 121: How robust are your results to the width of the time bins? For example, does the pattern observed still hold if the time bins are 1000 years? What about 5000 years?
Line 123: In the Materials and Methods, the downscaling algorithm is provided by Karger et al., no? Also important to go in the Methods: what is the temporal resolution of this climate dataset, and if it's different from the time bins, how was the data aggregated/downscaled temporally?
Line 123: Remove "The results indicate that"
Line 127: This isn't super clear from Figure 1b: maybe color code the lines based on Alaska vs. Siberia, or longitude?
Lines 150-157: What about diversity of functional characteristics? For example, is larger average height related to greater standard deviation in height? It might be interesting to relate trait spread to these competitive interactions (see, e.g., D'Andrea et al. 2020, Ecology, on niche partitioning), although that is likely beyond the scope of the current research.
Line 157: Change "which is" to "at", also Figure 1d needs an axis on the right for root length.
Line 187: "predominant" may be a little strong, as it's barely more than a majority in Figure 2C
Line 220: What is the unit of analysis here? All lakes over one time bin? Or the interactions within each lake? I'd put a n = ___ somewhere in this section.
Discussion:
As with the other two sections, the discussion is very clear, polished, and compelling. Overall, I think it might be slightly too long, so I've provided some options for cutting below.
Line 246: "…Swiss Alps", maybe cite?
Line 268: Do ASVs at higher taxonomic levels show more positive interactions? I would guess so, because any one species within the ASV has the potential to positively interact with another species. Do your results change if you aggregate species-level ASVs into genera/families?
Lines 271-283: The paragraph does a great job of setting the scene and describing the patterns observed!
Lines 284-286: I would rearrange these two sentences.
Lines 335-336: I understand that this heading is essentially the reverse of the previous, which makes sense, but I think that a lot of the material in here re-states what the previous said (that woody plants have more negative interactions and that negative interactions became more common with warming temperature). I think this section may benefit from another read-through focusing on removing information already mentioned in the previous one. Also see my second general point about the potential circularity.
Lines 337-345: Good!
Line 355: maybe "Exhibited"?
Line 364: less competitive given the current, warm, climate, correct? I would assume they would be more competitive in very cold climates.
Line 365: remove comma between "increase" and "as"
Lines 379-387: This section is good, but I think a little redundant, especially with Lines 313-320, so I think it could be shortened.
Lines 388-394: I really like the link to the current/future of the Arctic here, and I think this should be given a little bit more focus relative to the previous information in this section.
Materials and Methods
Line 462: add "occurrences" after "removing"
Line 463: Do you mean that you removed occurrences with coordinate resolution >5 km?
Line 476: I would say "unobserved", because with presence-only data like GBIF we can never be sure that the species is

absent from a location.
Lines 477-479: I'm a little confused by the methods used to select the background points. If the background points were selected specifically because they are in climate where the species is not observed, the habitat suitability of the taxon within this area will be over-represented in MaxEnt, and both the range size and the extent of occurrence will be larger than expected. Further, wouldn't using the OCSVM to determine background points essentially model the environmental niche of the species anyway? This method a priori assumes that the taxa are constrained by both variables somewhat equally (combined MAT and July temp), and makes the use of MaxEnt circular. From my understanding, MaxEnt and other presence-only models generally work better when selecting a random set of background points, constrained by geographic space (see Barbet-Massin et al. 2012, Methods in Ecology and Evolution, and Whitford et al. 2024, Ecological Modelling).
Line 498: I'm glad to see the Boyce Index being used to evaluate model performance! Was it calculated using a sliding window or discrete bins? Also, Looking at the supplementary Figure 4, it looks like some models were no better than random and some were even worse than random. My guess is that these models performed poorly because of the geographic constraints put on the background points, leading to overfitting and non-transferability. Were the species with those models still included in the environmentally constrained null model?
Line 510: Not 2,000 year increments, like the time slices of the empirical data?
Line 524: There is a missing parenthesis somewhere in this equation.
Line 530-531: The environmentally constrained null models have not been explained yet; I would put this sentence after the explanation.
Line 540: why 22 bins? Also, pairwise C-score of the null model, correct?
Line 543: What does "mean limits" mean in this context?
Line 551-570: This is a really interesting method! I would make it a little bit more clear here that for this you're only piecing apart the influence of dispersal limitation on negative interactions (as suggested by the excellent Supplementary Figure 3).
Lines 559-560: Only looking at species pairs with non-differing centroids is a very good idea, but it could skip over some positive interactions, right? For example, if two species have different centroids but associate more than expected based on climate, wouldn't that be evidence for positive interactions?
Figure 1:
This is a beautiful figure!
Figure 2:
I really like this figure, especially the consistent color scheme between Figures 2a-2d! Figure 2a could benefit from a legend like the others, describing which links are positive and which are negative. Also, it's a little hard to figure out which boxplots in Figure 2b correspond to which categories (maybe space them out or add dividers).
Figure 3: Maybe reword "Wood plants abundance" to "Abundance of woody plants"?
Figure 4: I appreciate the explanation of the relationship between interactions and beta diversity. However, I'm not sure what "distribution range of _Taxon 1_) is supposed to be telling the reader (same with High rate of taxon loss of _Taxon 1_, and "distribution range in the bottom corner").

(Remarks on code availability)
The code was clear and easy to read; thank you!

Reviewer #3

(Remarks to the Author)
Review of the manuscript entitled "Woody plant encroachment enhances negative interactions in Arctic plant communities over the last 24,000 years"
This manuscript analyzes past plant interactions using sedaDNA records from nine lakes distributed across Siberia and Alaska. To achieve this, the authors transformed plant composition tables into plant functional traits (specifically root length and plant height), traits known to shape community structure through their role in competition for light, water, and nutrients. They used environmentally constrained null models to differentiate between biotic interactions and co-occurrence patterns driven by environmental filtering. They also integrated species distribution models to account for species dispersal limitations.
To my knowledge, such a question has not yet been addressed from a paleoecological perspective, which makes this study highly original. The conceptual framework and the statistical approaches proposed are, in my view, very relevant (although I am not in a position to assess the mathematical details behind the models).
The main conclusion of the paper is that colder glacial periods favored positive plant interactions (facilitation), whereas warmer conditions led to more negative interactions (competition). This result aligns well with the Stress Gradient Hypothesis in ecology. Furthermore, the authors show that facilitation mainly involved herbaceous taxa, while competition was more frequently associated with woody species, likely due to competition for light and access to water/nutrients. These interaction patterns, well-documented in ecological studies, reinforce the robustness of the inferred interaction networks across different growth forms. The study also highlights the indirect role of temperature in modulating plant-plant interactions. These conclusions are significant in the context of ongoing global warming and are broadly relevant to several scientific communities, including ecology, paleoecology, and sedaDNA research. I particularly appreciated the section addressing the limitations of the approach and potential biases in the inferences.

Specific comments and questions:
• P3, L82–83: I would avoid stating that the transition from the glacial period to the Holocene is an analogue to current global warming. I suggest modifying to: "may provide an analogue", as you do in the abstract. This is especially important since, although the area was largely deglaciated during the glacial period, soil development (e.g., weathering processes, soil thickness, nutrient availability, and water retention) was likely very different from today.
• Figure 1C: This panel shows Salicaceae, which is a taxonomic group, not a growth form. Please adjust the legend

accordingly. Also, I'm not entirely sure why you chose to include Salicaceae specifically—could you justify this choice? If not essential, consider removing it. Alternatively, you could also include Ericaceae, which likely increased during the Holocene with podzol development. These taxa are also mentioned later in the section "Effects of woody encroachment on plant interaction shifts."
• P7, L140–142: When you state "60% of the flora…", it reads as if you're referring to actual landscape composition, whereas this is in fact the proportion within your sedaDNA record. Please clarify.
• P8, L182–185: What exactly do you mean by "broad taxonomic involvement"? Do you mean a large number of taxa were involved? If so, it would help to give more examples, beyond Saxifraga oppositifolia.
• Figure 2: Perhaps I missed it, but how exactly was the network constructed? Which software or R package did you use?
• P12, L259–261: I'm not sure I understand what you mean by "resolution of network". Also, I'm wondering how the use of 2,000-year windows might affect your inference of plant-plant interactions. I understand that this choice helps reduce age-depth uncertainties and increases the number of data points, but doesn't it risk creating interactions between taxa that didn't actually co-occur? Perhaps I misunderstood, or perhaps taxonomic composition is relatively stable within each time window. In any case, I suggest discussing this point further for clarity.
• L300–301: It's not immediately obvious that "the spread of woody taxa facilitated the formation of mycorrhizal associations." Could you elaborate on this mechanism? Perhaps the following sentences aim to clarify this, but currently it's a bit unclear. A reformulation or brief explanation would improve readability here.
• Lastly, I don't know whether modern studies have shown that certain plant species can switch the direction of their interactions (from positive to negative or vice versa) depending on environmental context. But I found this finding particularly interesting—perhaps even fascinating (also, I recently saw my first Cassiope tetragona!). If such interaction plasticity has been observed in modern systems, it would be great to cite relevant studies. If not, this is a remarkable result that deserves to be emphasized more strongly, to my mind.

(Remarks on code availability)
I did not check all the codes, just have a Quick Look. but I think that a readme file is missing. Or I missed it.

Version 1:

Reviewer comments:

Reviewer #1

(Remarks to the Author)
The authors have addressed my comments sufficiently and I applaud them for the revision of an excellent manuscript.

(Remarks on code availability)

Reviewer #2

(Remarks to the Author)
Thank you for giving me the opportunity to read the revised version of this manuscript. I am very impressed by the work done between the submissions, and I believe the manuscript is much improved. I only have a few small notes to highlight, but otherwise, I think it is an important contribution to the field and a very well-written work. It was a pleasure to read!

Line 79: "..shape the plant interactions." Maybe revise to "...shape how plants interact."?

Lines 83, 89: I think in these two places, putting a time on the glacial period, Holocene, and Last Glacial Maximum (for example, and using rough time estimates; "... from the glacial period (>14,000 years ago) to the Holocene (~11,000 years ago)" can be useful to orient the reader.

Line 96: Environmentally constrained null model --> Environmentally constrained null models
Line 99: "It proivdes insight..." --> "They provide insight..."

Line 241: "Consistently..." Maybe "Consistent with these observations..."? You talk about consistency through time as well in the paragraph, but I think here you're discussing how your results are consistent with previous work/observations, so maybe a little clarification is needed.

Lines 275-277: This caveat'limitation is about the SDMs, so maybe move it further up in the paragraph(where you talk more about the SDM methods, etc.)? Also, I think this paragraph could be split into two to make it easier to read and not as much of a list: one paragraph focusing on data limitations (where you talk about, e.g., the sedaDNA, taphonomy, and the temporal resolution, etc.), and model limitations.

Figure 4 caption: "impact of plant interactions on community" --> either "communities", or something slightly more specific like "community structure"

(Remarks on code availability)

Reviewer #3

(Remarks to the Author)
All the comments have been addressed and I consider that the manuscript is now ready for publication.

(Remarks on code availability)

Open Access This Peer Review File is licensed under a Creative Commons Attribution 4.0 International License, which permits use, sharing, adaptation, distribution and reproduction in any medium or format, as long as you give appropriate credit to the original author(s) and the source, provide a link to the Creative Commons license, and indicate if changes were made.
In cases where reviewers are anonymous, credit should be given to 'Anonymous Referee' and the source.
The images or other third party material in this Peer Review File are included in the article's Creative Commons license, unless indicated otherwise in a credit line to the material. If material is not included in the article's Creative Commons license and your intended use is not permitted by statutory regulation or exceeds the permitted use, you will need to obtain permission directly from the copyright holder.
To view a copy of this license, visit https://creativecommons.org/licenses/by/4.0/

REVIEWER COMMENTS

Note: All line references refer to the version of the manuscript with tracked changes

Reviewer #1 (Remarks to the Author):

This manuscript is an excellently written reconstruction of plant interactions in the Arctic that is highly relevant for current climatic trends. The authors provide detailed and sound ecological interpretations of their model and expand on this previously published dataset. The authors have also graciously provided well-documented code and datasheets to follow their analysis. I expect their approach and results will be of interest to a large part of the scientific community. I have one major concern that I would like the authors to comment on:

Response: We are thankful to Reviewer #1 for their constructive comments.

Comment1: Many of the lakes used in the study have large and ecologically varied catchments (e.g. Lake Illirney's 1200 km2 catchment comprising Larix forest and high alpine environments). sedaDNA data collapses all of these habitats into one datapoint whereas in reality, taxa may not have existed in close proximity to one another. Some discussion is warranted on how covariation of these taxa in ecologically distinct areas could be interpreted as interaction by the environmentally constrained null model.

Response1: Agree, revised.

New text: **Line 289-294** Additionally, taxa confined to specific microhabitats within a catchment may be undetected by species distribution models, resulting in inaccurate presence–absence predictions. Lastly, sedaNDA captures genetic signals from the entire catchment, potentially detecting these localised taxa and producing spurious co-occurrence patterns with other species, thereby overestimating the plant interactions.

Comment2: Additionally: **L427-429**: Some elaboration on what "substantial divergence from other samples" constitutes is needed. Are samples being compared against each other or is it just the PCR replicates within a sample? The referenced paper (Courtin et al. 2025) details clustering PCR replicates within a sample, but the phrasing of "PCR samples were excluded if their replicates showed substantial divergence from other samples" implies other samples are being compared.

Response2: Sorry for the misphrasing; the comparison refers solely to PCR replicates within a sample. We have rephrased the sentence.

New text: **Line 466-469** PCR replicates were excluded if they showed substantial divergence from other replicates within the same sample, indicating low quality, or if replicates failed to cluster, suggesting distinct compositions within replicates.

Reviewer #2 (Remarks to the Author):

In the manuscript "Woody plant encroachment enhances negative interactions in Arctic plant communities over the last 24,000 years", the authors use sedaDNA from several Siberian/Alaskan lakes to examine patterns of ecological interactions throughout the late Pleistocene and Holocene, and how these interactions link to the functional characteristics of the communities there.

Overall, I found the manuscript engaging and written beautifully, and I am excited about the results presented. In particular, I was impressed by the environmentally constrained null-model of community assembly. The results presented were clear and defended very well. However, there are few points of clarification I would like addressed about the methods and the interpretation of the results.

Response: We are thankful to Reviewer #2 for their constructive comments.

Comment 1: First, I had a few notes about subsampling: I am happy to see that the sedaDNA dataset was subsampled to the same base count (20k) for each 2k year time period, and in your code you make sure each lake has 8 time slices, which is great! However, some lakes could still have more layers/distinct time samples within than others within a particular bin, which could lead to inflated estimates of coexistence. Therefore, it might be useful to not only rarefy on the number of base reads, but also to the minimum number of distinct time periods observed within each lake/time bin combination. In addition, how robust are your results to spatial variation? I.e., if one lake were removed from the analyses, how do the results change?

Response 1: The purpose of resampling within each bin (potentially with different sample sizes) was to retain more reads for each lake at each time slice, under the assumption that samples were more homogeneous within a lake at a given time bin. To account for the effect of different sample numbers within a time bin, one sample was randomly selected from each lake at each time slice, restricting the analysis to the minimum number of distinct time periods common to all lakes. Each selected sample was then rarefied to 2,000 reads for standardisation, as most samples originally contained more than 2,000 reads. Consequently, only a few samples were discarded; however, a large number of reads were removed from most samples, whose original read counts ranged from tens of thousands to hundreds of thousands. The network results based on the selected and rarefied samples (2,000 reads each) are shown below and in the supplementary files. According to the network plots, the proportion of positive and negative interaction patterns remained stable, although the total number of links decreased due to read removal, and some taxa were no longer represented. The time-bin results with different sample numbers are presented in the main text, the minimum sample-size results are provided in the supplementary information.

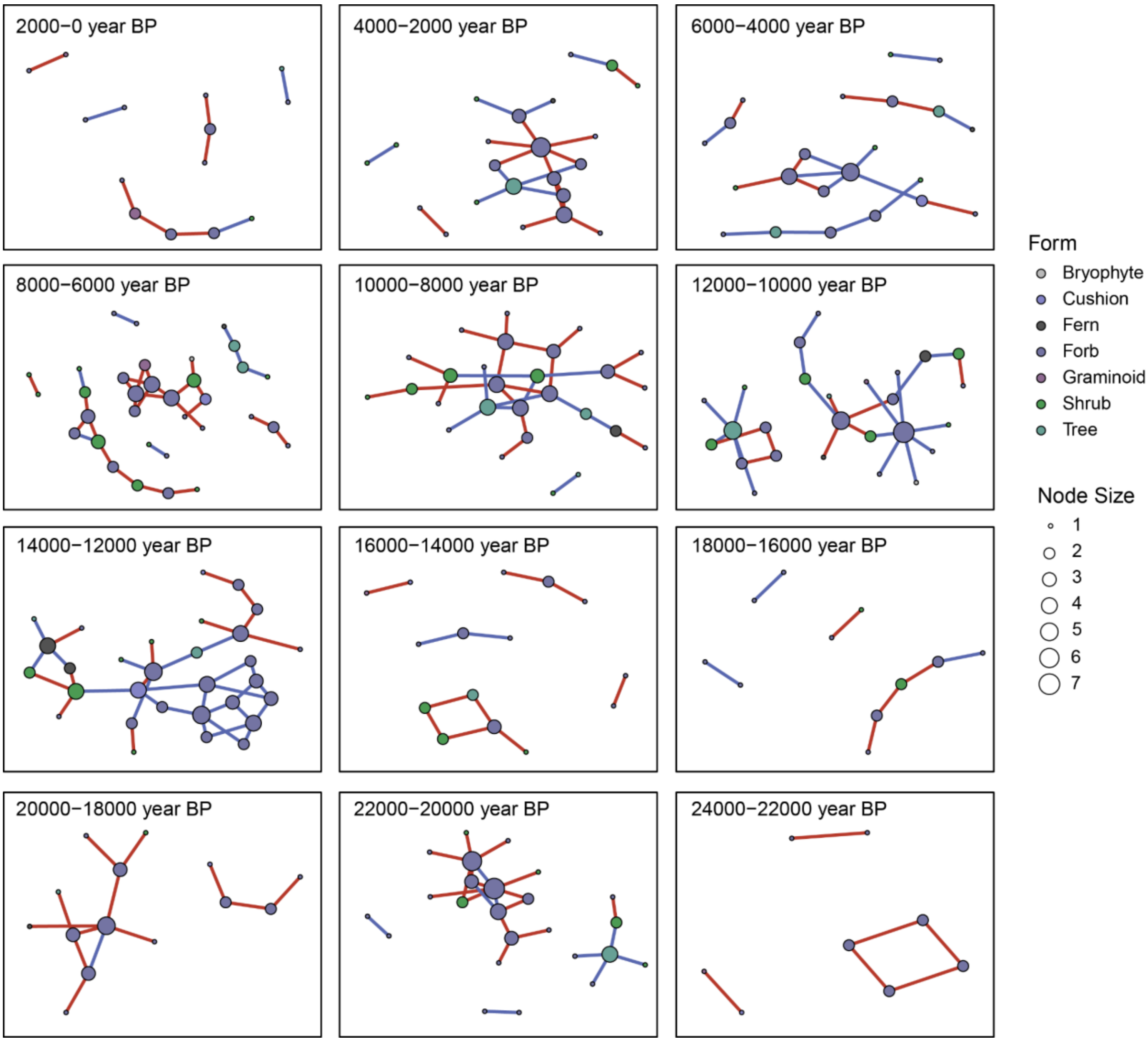

Supplementary Figure 5. Temporal dynamics of the plant interaction network across 2000-year time slices, with one sample each lake each timeslice and rarefied to 2,000 reads to standardise the samples and read numbers within time bins. Nodes represent taxa, with different growth-forms indicated by colour. Edges between nodes represent interactions between taxa, with red edges indicating positive interactions and blue edges indicating negative interactions. Node size corresponds to the number of connections (i.e., node degree).

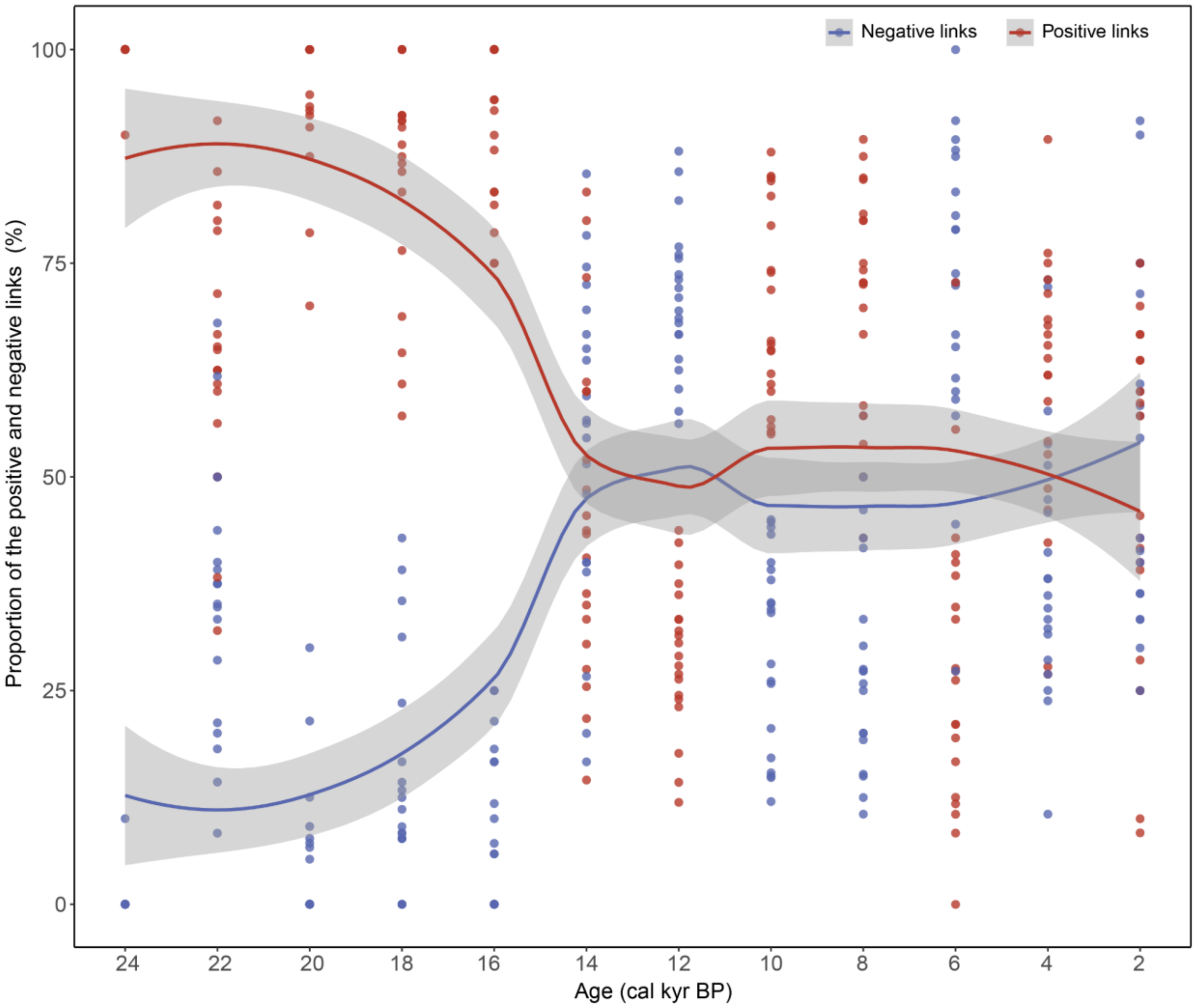

Supplementary Figure 6. Temporal trends in the relative proportion of positive and negative interactions, with one sample each lake each timeslice and rarefied to 2,000 reads to standardise the samples and read numbers within time bins.

To test the robustness of the results to spatial variation, we repeated the analysis after randomly removing one lake. The results indicate that the overall pattern remained unchanged. The results were also shown in the supplementary information.

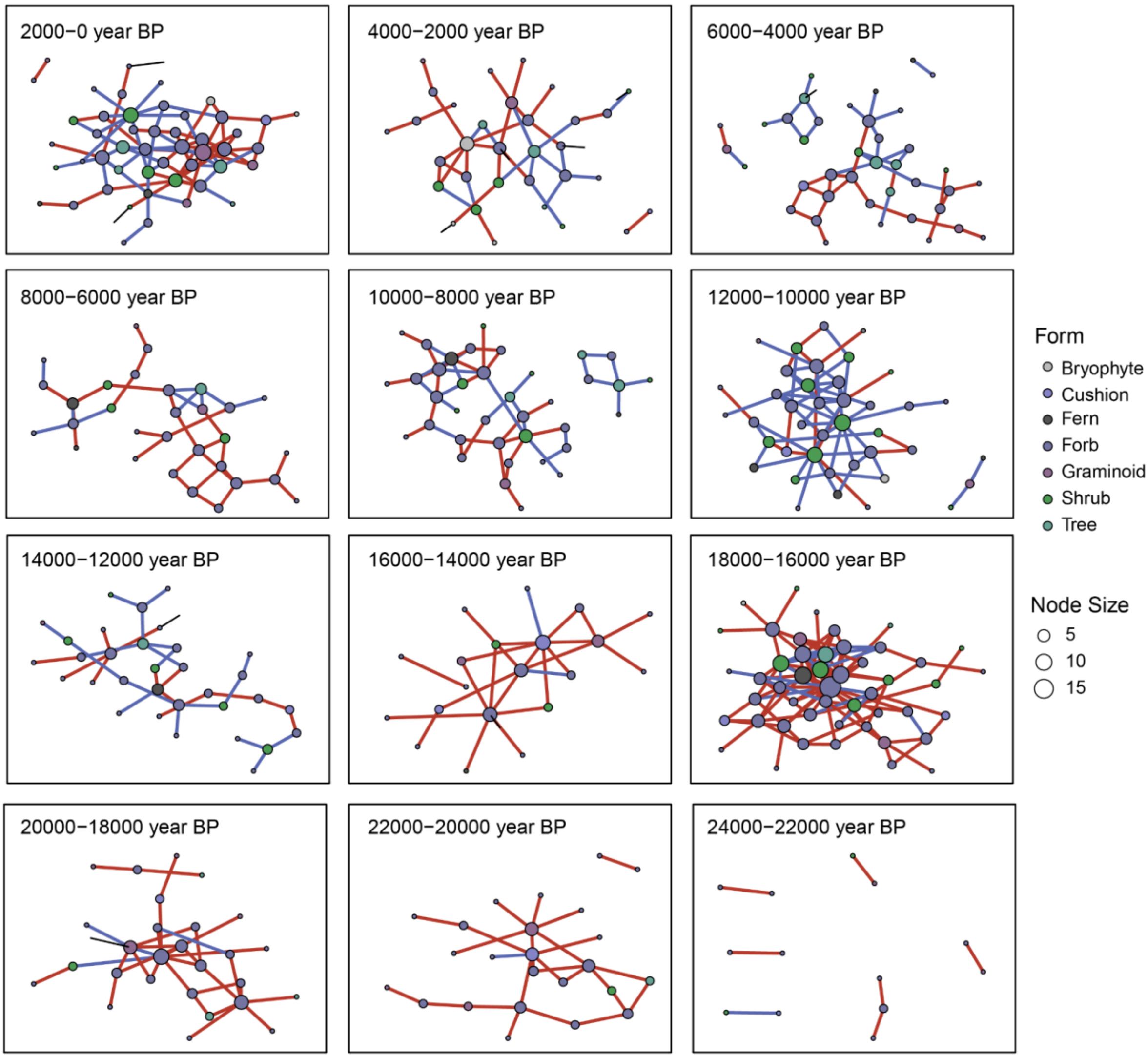

Supplementary Figure 7. Temporal dynamics of the plant interaction network across 2000-year time slices, with each lake rarefied to 10,000 reads each time slice, and one lake site randomly excluded from the analysis. Nodes represent taxa, with different growth-forms indicated by colour. Edges between nodes represent interactions between taxa, with red edges indicating positive interactions and blue edges indicating negative interactions. Node size corresponds to the number of connections (i.e., node degree).

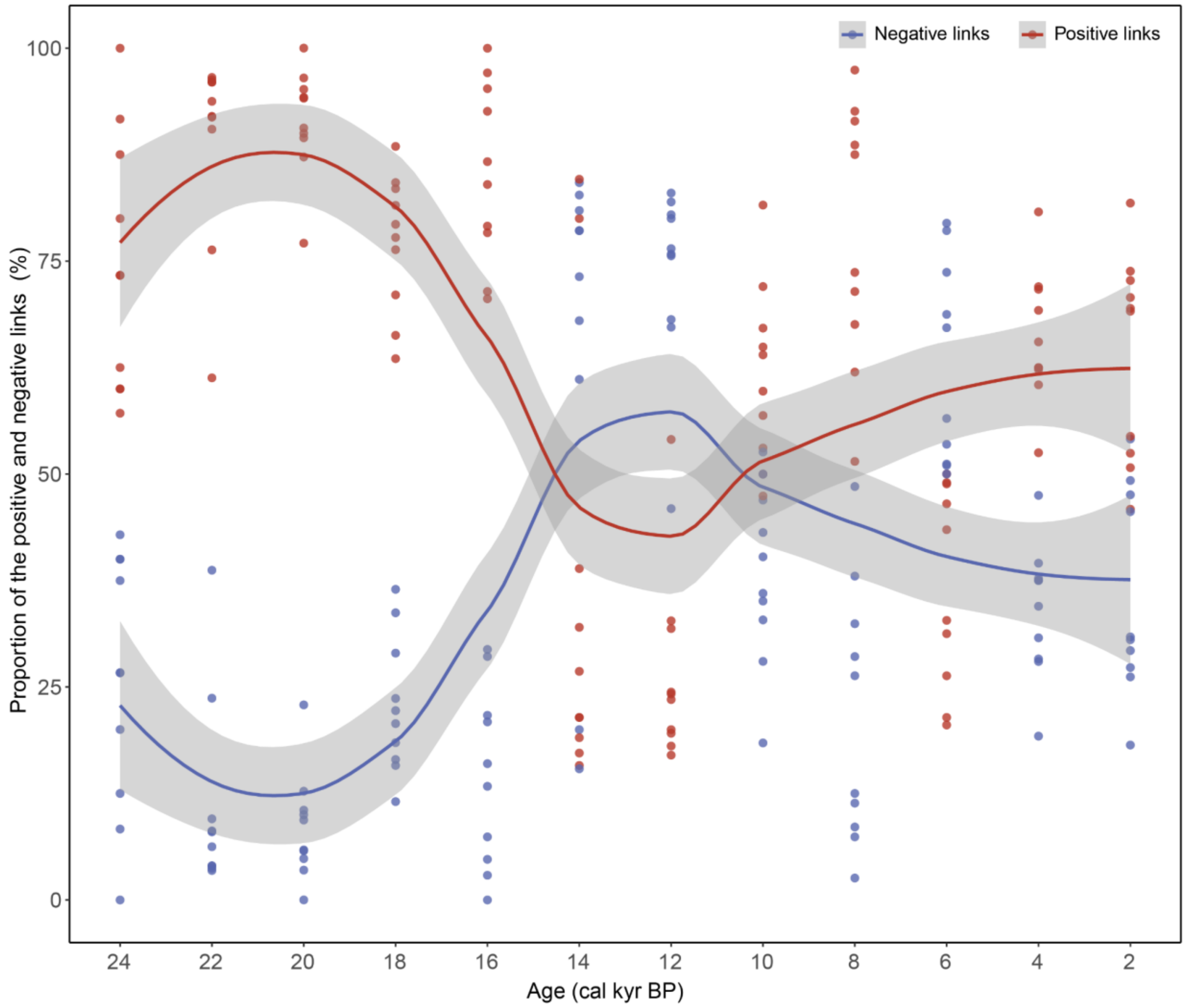

Supplementary Figure 8. Temporal trends in the relative proportion of positive and negative interactions, with each lake rarefied to 10,000 reads each time slice, and one lake site randomly excluded from the analysis.

Comment 2: Second, the abstract suggests that temperature and plant interactions jointly supported the expansion of woody taxa, but the discussion and the SEM suggest the other way around; that the rising temperature and expansion of woody taxa drove increased competition in the Arctic. I worry that these interpretations run the risk of being somewhat circular. I think this research could benefit from restating the hypotheses/results slightly. For example, is the idea that, when temperatures rose, woody plants began expanding, and this expansion was exacerbated by the increase in competition that occurred (in which case, maybe you could find a lag between the arrival of woody taxa and their dominance relating to the proportion of negative interactions)? Or maybe that the negative interactions drove the extirpation/taxon loss of cushion plants (in which case, you could incorporate cushion plant proportion or richness into your SEM)? Further, I think it might be worth it to test the direct influence of temperature on the prevalence of negative links (i.e., through your SEM), to disentangle these effects slightly.
Response 2: Thanks for pointing this out.We revised the abstract accordingly. The idea is that temperature increase likely promoted woody-taxa expansion, which subsequently altered

plant interactions due to the greater height and root length of woody species. These altered interactions further influenced community dynamics by facilitating additional woody-taxa expansion and increasing risks to native taxa. We also tested the direct effect of temperature on negative links; however, the model yielded $\chi^2 = 0.626$, df = 0, p = NA, RMSEA = 0.0, CFI = 1.0, GFI = 1.0, and SRMR = 0.001. Because the degrees of freedom were zero, indicating that the model was saturated and could not be statistically evaluated, we did not adopt this model in the main text.

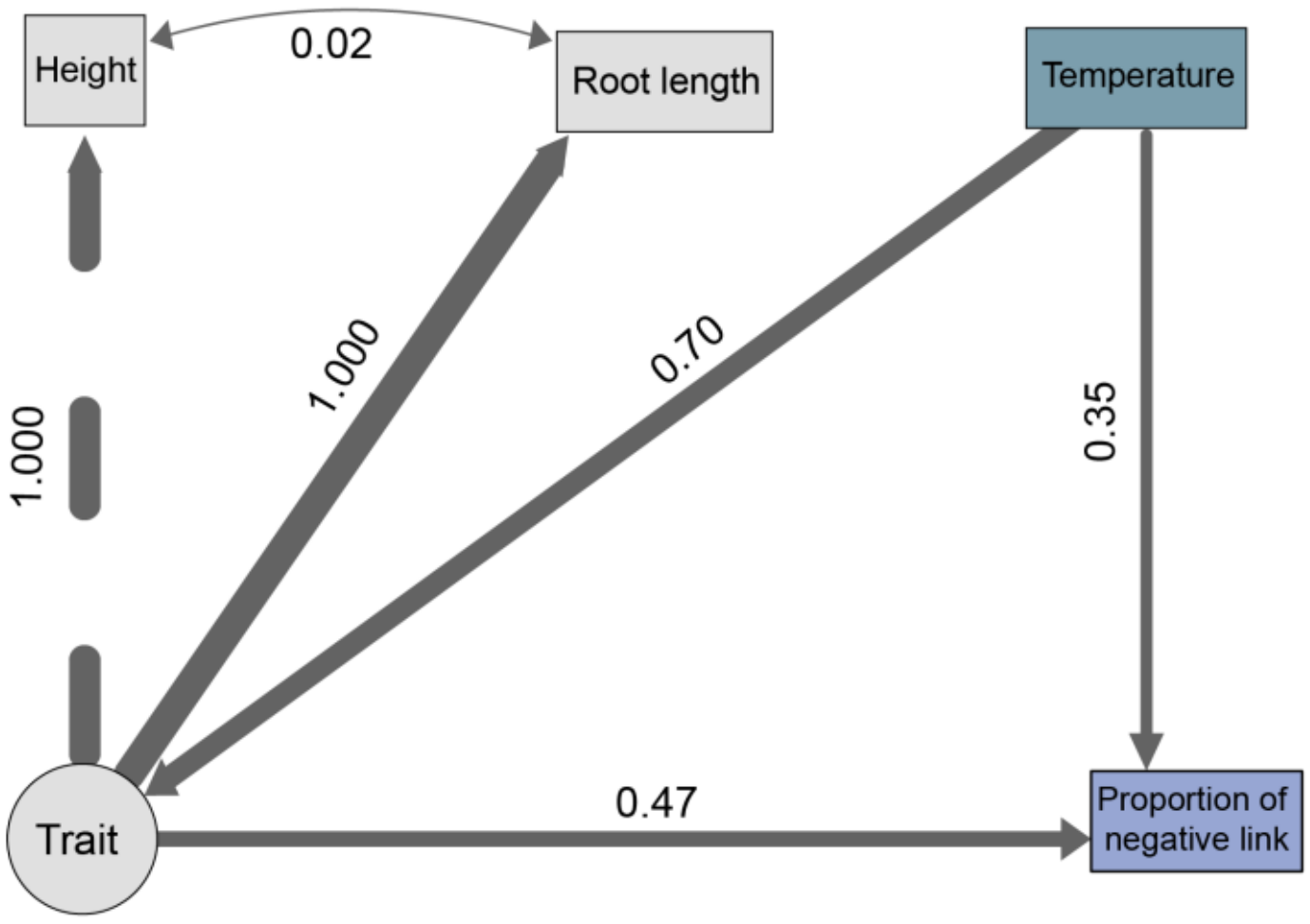

Figure. Structural equation model showing the relationships between response variables and predictors, with direct influence of temperature on negative links.

New text: **Line 44-51** We suggest that climate (an external factor) supported the widespread woody taxa expansion at the end of last glacial, which shifted the plant interaction (an internal process) that further exacerbate the expansion. This mechanism may provide an analogy with contemporary "arctic greening". Furthermore, woody encroachment is likely to constrain the geographical ranges of native species, increasing the risk of local native taxa loss, while enhancing beta diversity.

Comment 3: Thank you for giving me an opportunity to read this interesting research! I've organized more minor comments section by section below:

Introduction:
The introduction does an excellent job of introducing the reader to all of the separate aspects of the research!

Line 54: Replace "All the" with either "our" or "current"
Response 3: Thanks, revised.

Comment 4: Line 68: "While cushion plants provide insights" might be streamlined into "However," or something similar.
Response 4: Thanks, revised.

Comment 5: Line 85: "…due to polar amplification" maybe cite?
Response 5: Thanks, done.

Comment 6: Lines 93-98: I think this section could mostly be removed: instead focus on what you did do (something like "Environmentally-constrained models can ______, providing insight into ____, but these models have not been used in _______"
Response 6: Agree, revised.
New text: Line 99-103 Environmentally constrained null model can partially account for environmental influences, dispersal limitations, and stochastic effects on species co-occurrence, attributing the residual co-occurrence to the effects of biotic interactions. It provides insight into disentangling the plant interaction from plant co-occurrence, but this approach has not yet been applied to investigate long-term species interactions using proxy data.

Comment 7: Lines 102-111: Excellent paragraph! Very clear!
Results:
The title headings are very informative, and I really like that they reflect the last sentence in the introduction.
Line 118: Link to Figure 1 here
Response 7: Thanks, done.

Comment 8: Line 121: How robust are your results to the width of the time bins? For example, does the pattern observed still hold if the time bins are 1000 years? What about 5000 years?
Response 8: For the 1000 timeslice, the sample size is small, which means that we have at most nine samples for each timeslice, the small sample size can not support the C score has significant difference with the C score calculated from the null model, therefore the small sample size limit the calculation rather than there is no plant interaction, therefore we tries to use the 2000 years time bins.

To test the robustness of our results across different timeslices, we also examined the 3000- and 4000-year timeslices (both divisible by 24,000 years). These longer timeslices include fewer networks, but as the time window increases, more taxa (i.e. all taxa present within the 3000- or 4000-year timeslice) are involved in the co-occurrence patterns. Therefore, compared with the 2000-year timeslices, the 3000- and 4000-year timeslices are more likely to detect positive interactions. However, if taxa pairs have negative interactions, they may not co-occur at all, because strong negative interactions can prevent one taxon from appearing. Consequently, both positive and negative interactions may increase in absolute number, but the increase in positive interactions is greater, leading to a higher proportion of positive interactions.

3000 timeslice result

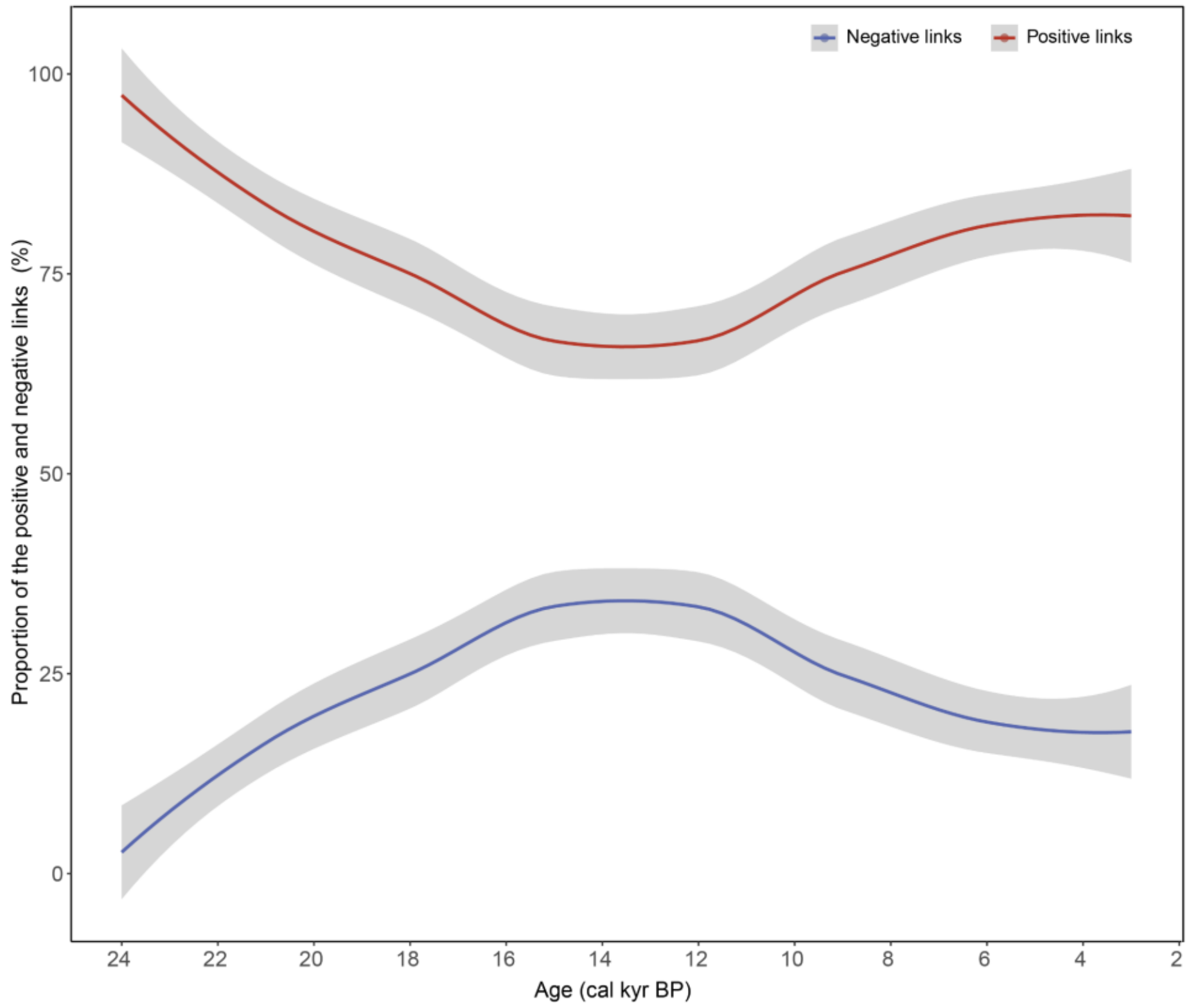

Supplementary Figure 9. Temporal trends in the relative proportion of positive and negative interactions, with a 3,000-year timeslice.

4000 timeslice result

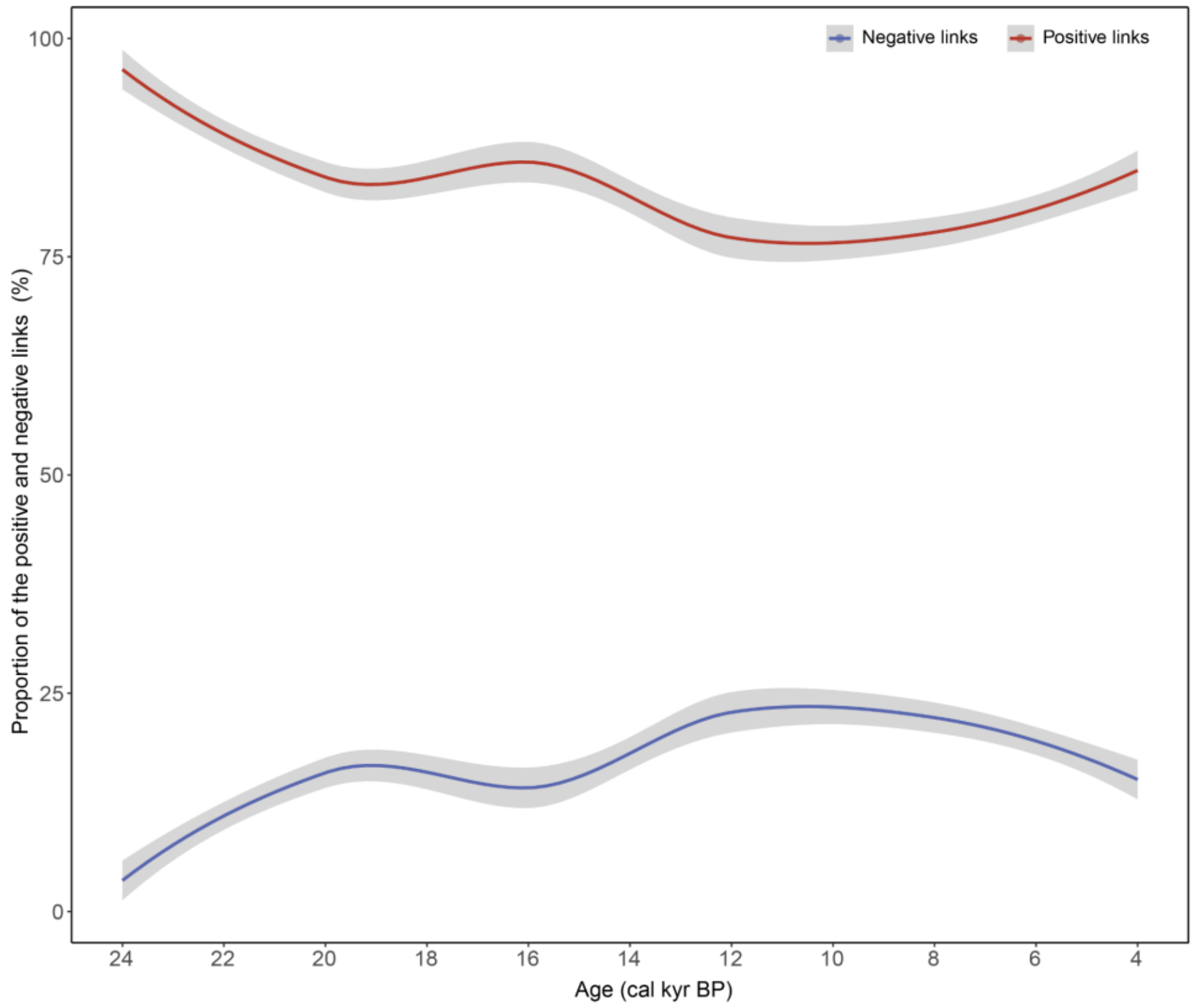

Supplementary Figure 10. Temporal trends in the relative proportion of positive and negative interactions, with a 4,000-year timeslice.

Comment 9: Line 123: In the Materials and Methods, the downscaling algorithm is provided by Karger et al., no? Also important to go in the Methods: what is the temporal resolution of this climate dataset, and if it's different from the time bins, how was the data aggregated/downscaled temporally?
Response 9: Sorry for the confusing expression. The downscaling algorithm follows Karger et al. The temporal resolution of this climate dataset is monthly, but we require temperature data spanning thousands of years. To address this, we divided one thousand years into ten intervals of one hundred years each. For each 100-year interval, the last year was selected as the representative year, since temperature variation within each interval is minimal. The mean annual temperature for the representative year was calculated as the average of its 12 monthly values. January and July temperatures were taken directly from the corresponding monthly data. Finally, for each one-thousand-year time bin, the mean of the ten representative years was used as the value.

New text: **Line 495-502** The raw temperature data have a monthly temporal resolution, whereas this study requires temperature data at a thousand-year resolution. Therefore, the raw data were divided into 100-year intervals. For each interval, the last year was selected as the representative year, and each month's temperature data of that year was used to calculate the spatially downscaled temperature. The mean annual temperature for the representative year was calculated as the average of its 12 monthly temperatures. January and July temperatures were taken directly from the corresponding monthly data. Finally, the mean of the ten representative years was used as the final value for each thousand years.

Comment 10: Line 123: Remove "The results indicate that"
Response 10: Thanks, done.

Comment 11: Line 127: This isn't super clear from Figure 1b: maybe color code the lines based on Alaska vs. Siberia, or longitude?
Response 11: Done, the temperature in the Alaska region was indicated by dark grey, and that of the Siberia region was indicated by light grey.

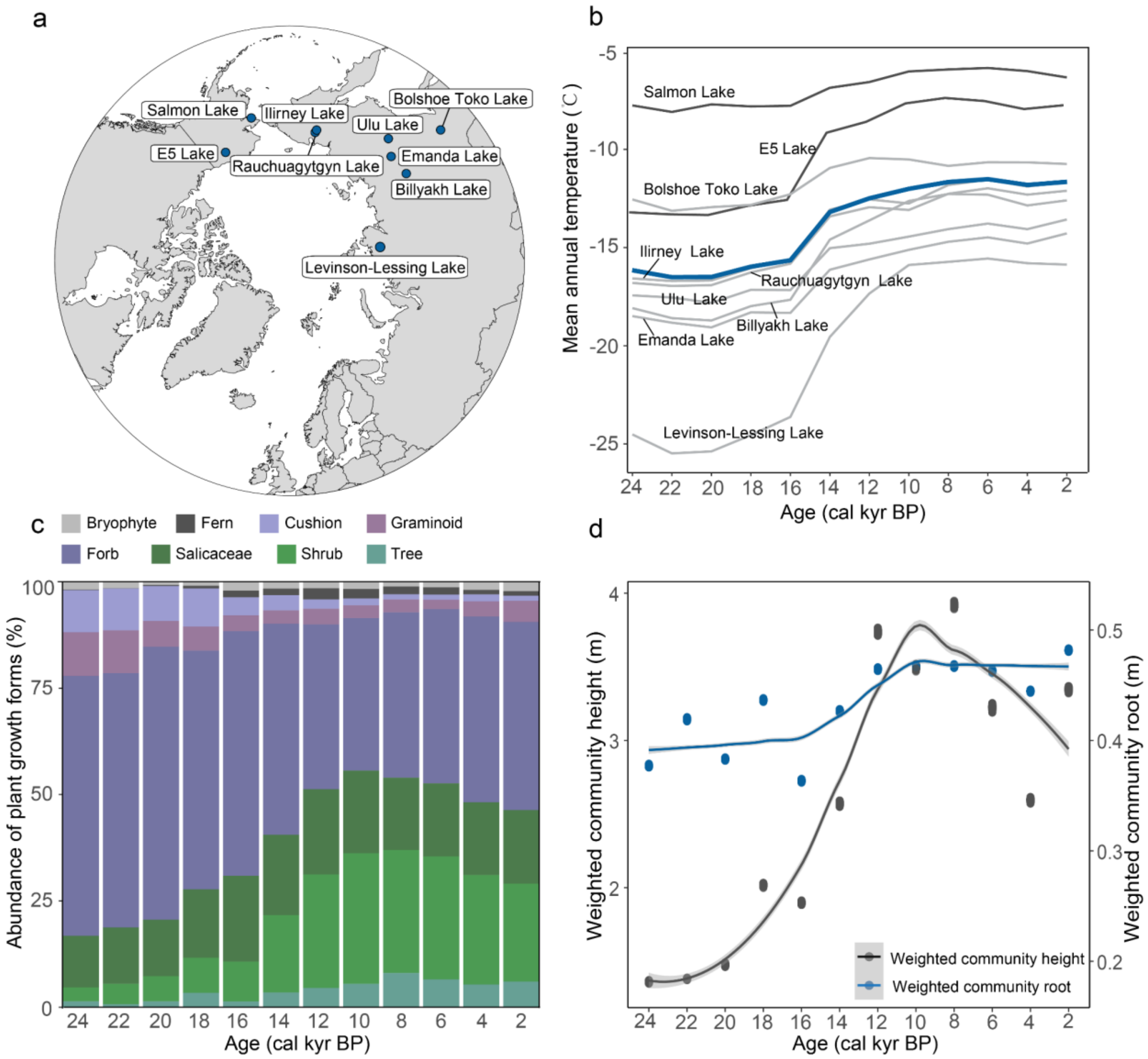

Figure 1. Mean annual temperature, plant growth-form abundance, and community weighted trait over time. (a) Locations of the nine lakes in northeastern Siberia and Alaska. (b) Temporal variations in mean annual temperature (see Materials and Methods). The thin dark grey curves show site-specific mean annual temperatures in Alaska region, the light grey curves show site-specific mean annual temperatures in Siberia region, whereas the bold blue curve represents the mean across all study sites. (c) Temporal dynamics in the proportion of transformed counts, representing the relative abundance of distinct plant growth-forms, are depicted in different colours. Within the shrub growth form, Salicaceae was shown specifically due to its high read counts. (d) Temporal changes in community-weighted traits. Traits were derived by summing the trait values of individual ASVs weighted by their relative abundances.

Comment 12: Lines 150-157: What about diversity of functional characteristics? For example, is larger average height related to greater standard deviation in height? It might be interesting to relate trait spread to these competitive interactions (see, e.g., D'Andrea et al. 2020, Ecology, on niche partitioning), although that is likely beyond the scope of the current research.

Response 12: We examined the relationship between community mean height and its standard deviation across time, and found a strong positive correlation ($r = 0.907$, $p < 0.001$), indicating that communities with taller average stature also exhibit greater functional spread

in height. However, as a deeper investigation of this pattern in relation to competitive interactions and niche partitioning falls beyond the scope of the current study, we did not pursue this further.

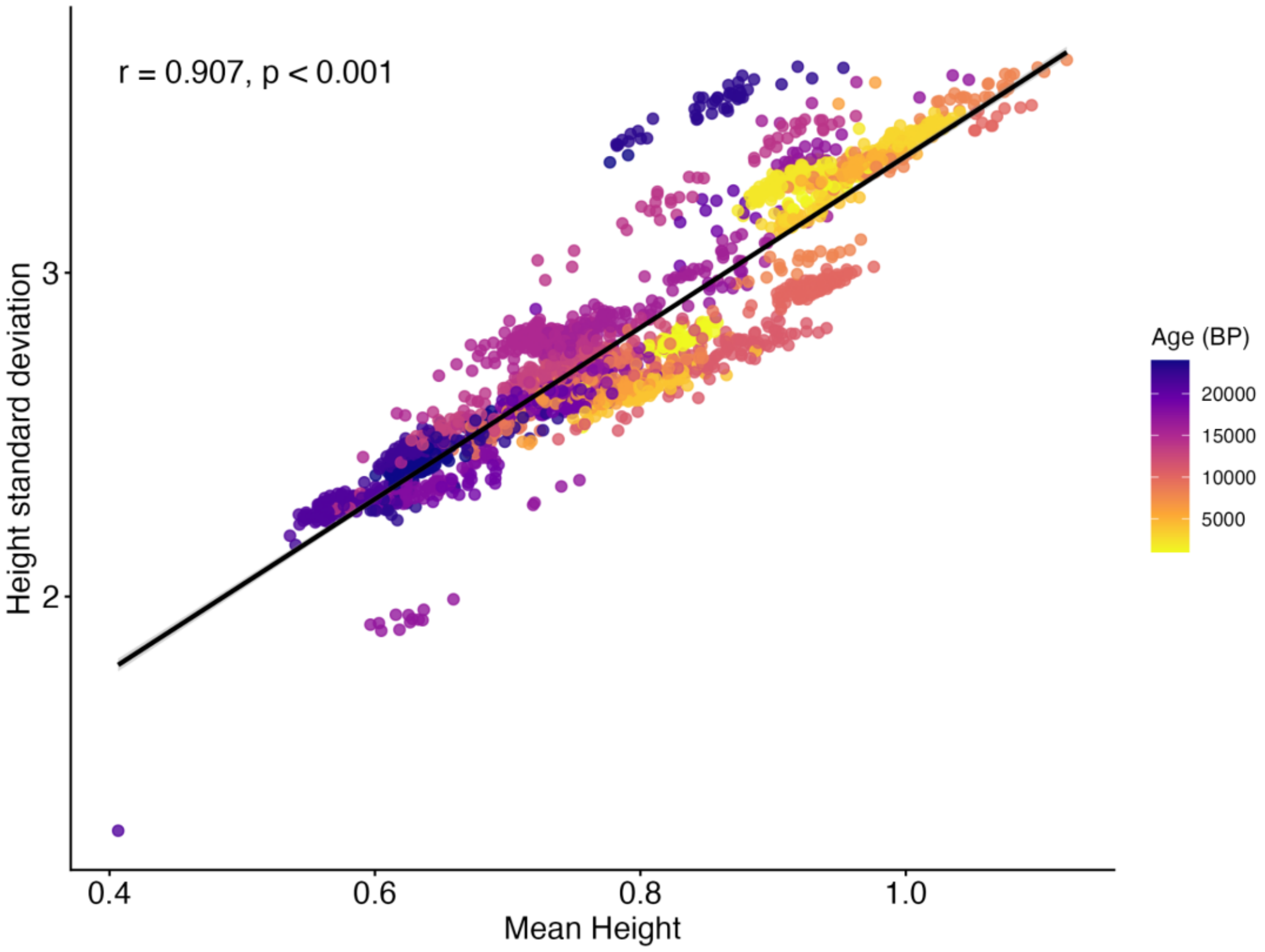

Figure. The relationship between the community mean plant height and the community height standard deviation.

Comment 13: Line 157: Change "which is" to "at", also Figure 1d needs an axis on the right for root length.
Response 13: revised, Figure 1d has been re-plotted because the previous version used the old resampling dataset.

Comment 14: Line 187: "predominant" may be a little strong, as it's barely more than a majority in Figure 2C
Response 14: Thanks, revised.
New text: Line 199-201 Thereafter, negative interactions became more common predominant but decreased again during the Late Holocene.

Comment 15: Line 220: What is the unit of analysis here? All lakes over one time bin? Or the interactions within each lake? I'd put a n = ___ somewhere in this section.
Response 15: The unit of analysis is all lakes combined within each time bin. This includes all 100 resampling iterations, each containing 12 time slices, yielding a total of 1,200 observations for analysis (n = 100 × 12).
New text: **Line 235-237** The model was based on time-series data for mean temperature, community functional traits, and the proportion of negative interactions across all sites (n = 1,200).

New text: Line 688-690 All variables were derived from 100 resampling iterations, each containing 12 time slices. This yielded a total of 1,200 observations for analysis (n = 100 × 12).

Discussion:
As with the other two sections, the discussion is very clear, polished, and compelling. Overall, I think it might be slightly too long, so I've provided some options for cutting below.

Comment 16: Line 246: "…Swiss Alps", maybe cite?
Response 16: Thanks, done.

Comment 17: Line 268: Do ASVs at higher taxonomic levels show more positive interactions? I would guess so, because any one species within the ASV has the potential to positively interact with another species. Do your results change if you aggregate species-level ASVs into genera/families?
Response 17: After aggregating species-level ASVs to the family level, the overall pattern of positive and negative interactions did not change, while there was a reduction in the proportion of positive interactions. This occurred because aggregation at the family or genus level did not show the between-species interaction within families and between species from different families. Instead, it shows the general tendency for species from different families, mainly the competition between the woody and non-woody species.

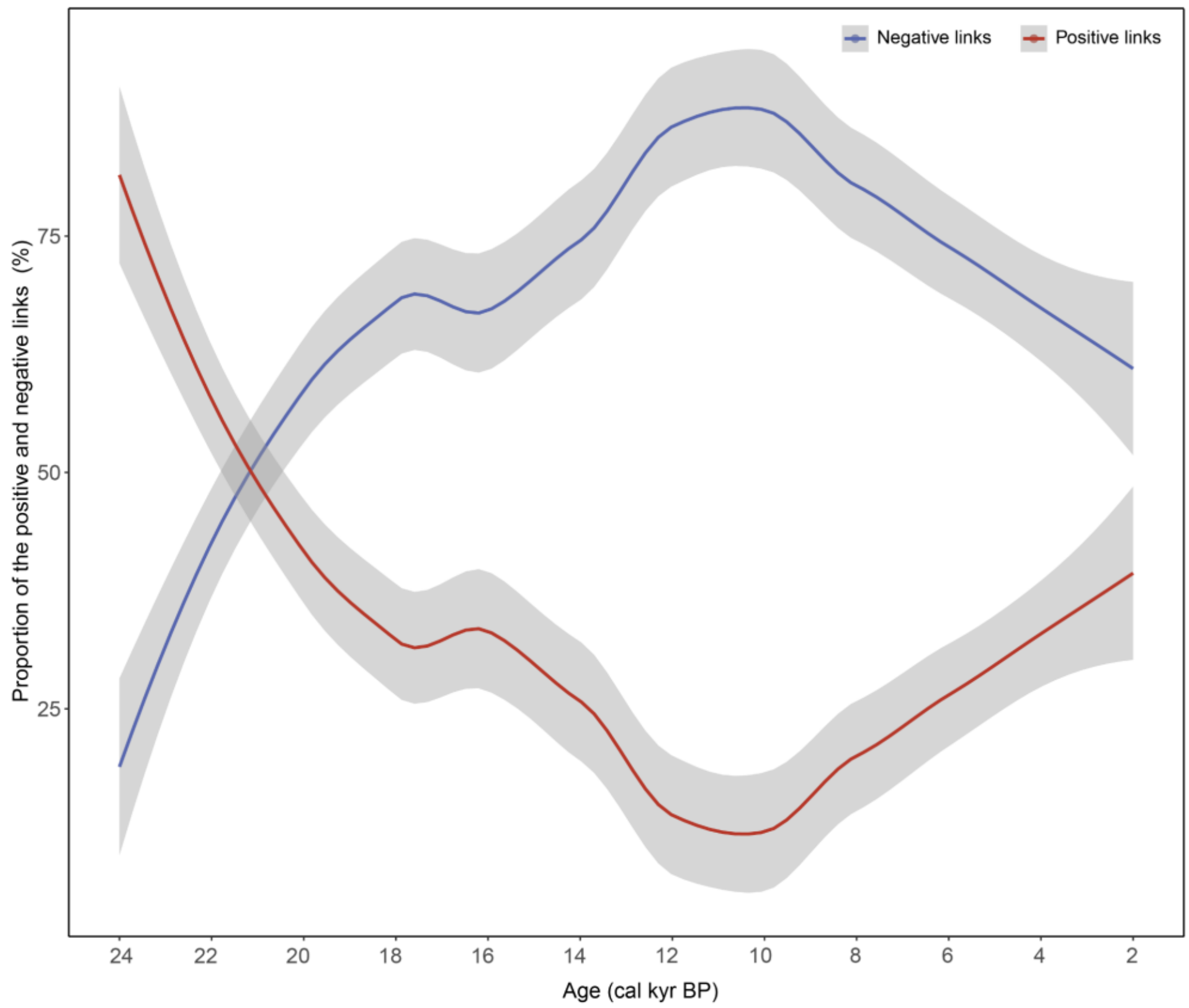

Supplementary Figure 11. Temporal trends in the relative proportion of positive and negative interactions at the family level.

Comment 18: Lines 271-283: The paragraph does a great job of setting the scene and describing the patterns observed!
Response 18: Thanks.

Comment 19: Lines 284-286: I would rearrange these two sentences.
Response 19: Thanks, rearranged.
New text Line 310-312 Plants interact through their phenotypic characteristics, and changes in plant functional traits can drive shifts in these interactions[42], with the greater height of encroaching woody taxa potentially contributing to aboveground light competition.

Comment 20: Lines 335-336: I understand that this heading is essentially the reverse of the previous, which makes sense, but I think that a lot of the material in here re-states what the previous said (that woody plants have more negative interactions and that negative interactions became more common with warming temperature). I think this section may benefit from another read-through focusing on removing information already mentioned in the previous one. Also see my second general point about the potential circularity.
Response 20: Thanks, we removed the information already mentioned before.

New text: Line 370-404 Intensified negative plant interactions resulting from woody taxa expansion further influence the plant community. During the glacial period (Figure 4a), positive interaction likely facilitated the establishment of new taxa and supported the survival of species that might have otherwise been constrained by environmental conditions [53]. However, this also rendered the plant community more vulnerable to invasions by other taxa. Positive interactions may have further contributed to the persistence of resident taxa by promoting their geographical range expansion. Our previous study found that, during the glacial period, the average range size of plant communities increased, with species facilitating each other's distribution [20]. For instance, *Silene acaulis*, which engages in positive interactions with multiple taxa, played a role in expanding species' ranges [9]. As a result, regional beta diversity remained low, as the widespread distribution of species promoted a more homogeneous plant composition. Furthermore, the positive plant interactions and relatively wide distribution range likely prevented the plant from going extinct, sustaining diversity even under harsh environmental conditions [54,55].

In contrast, during the interglacial period (Figure 4b), intensified negative interactions may constrain the establishment of new taxa by favouring native species with competitive advantages [56]. As a result, this area exhibited increased resistance to species migration, likely mediated through competition for light and resources, for example for *Larix*, as well as the release of allelopathic chemicals [57]. Shifts in plant–plant interactions may also influence the persistence of resident taxa, as stronger negative interactions could suppress population growth rates, restricting range expansion and potentially leading to range contraction [20,58,59]. As a result, these species face an increased likelihood of local extirpation, consistent with the findings of Courtin et al. [21], who find a higher than expected loss of taxa, ultimately leading to a decline in diversity. Moreover, herbaceous taxa, due to their traits, are less competitive and particularly vulnerable to local extinction in the study region (Figure 4b, species marked in purple circles), as noted by Courtin et al. [21]. However, regional beta diversity may increase, as competition fosters divergent community assemblages across sites (Figure 4b), enhancing spatial heterogeneity at broader scales [60]. Furthermore, our findings suggest that species with

advantageous traits under negative interactions are more likely to become dominant in plant communities [61] (Figure 4b, woody species marked in green circles).

Comment 21: Lines 337-345: Good!
Line 355: maybe “Exhibited”?
Response 21: Revised.

Comment 22: Line 364: less competitive given the current, warm, climate, correct? I would assume they would be more competitive in very cold climates.
Response 22: Agree.

Comment 23: Line 365: remove comma between “increase” and “as”
Response 23: Thanks, revised.

Comment 24: Lines 379-387: This section is good, but I think a little redundant, especially with Lines 313-320, so I think it could be shortened.
Response 24: Thanks, revised.

Comment 25: Lines 388-394: I really like the link to the current/future of the Arctic here, and I think this should be given a little bit more focus relative to the previous information in this section.
Response 25: Agree, we shorten the previous information and emphasise the future part.

New text Line 424-432 In contrast, native taxa with lower competitive ability, such as herbaceous taxa with shorter stature or shallower root systems, warrant greater attention, as they may decline or disappear before environmental conditions exceed their physiological tolerance, driven by negative interactions. Arctic plant community composition is shaped by more than just the northward migration of lower-latitude species, as biotic interactions may prevent certain herbaceous plants from establishing. Plant diversity in the Arctic will be determined by the dynamic balance between species gains and losses. Accordingly, regional beta diversity is driven by variation in species gains and losses across sites.

Materials and Methods
Comment 26: Line 462: add “occurrences” after “removing”
Response 26: Thanks, revised.

Comment 27: Line 463: Do you mean that you removed occurrences with coordinate resolution >5 km?
Response 27: We removed species whose coordinate resolution was coarser than 5 km. For example, if a coordinate was given as 57.5° N (which corresponds to a resolution greater than 5 km), it is not possible to determine accurately which 5 × 5 km grid cell it belongs to; we excluded it. If the coordinate had a finer resolution, for instance, 57.5555° N, which is below the 5 km threshold, then we kept it.

Comment 28: Line 476: I would say "unobserved", because with presence-only data like GBIF we can never be sure that the species is absent from a location.
Response 28: Agreed and revised.

Comment 29: Lines 477-479: I'm a little confused by the methods used to select the background points. If the background points were selected specifically because they are in climate where the species is not observed, the habitat suitability of the taxon within this area will be over-represented in MaxEnt, and both the range size and the extent of occurrence will be larger than expected. Further, wouldn't using the OCSVM to determine background points essentially model the environmental niche of the species anyway? This method a priori assumes that the taxa are constrained by both variables somewhat equally (combined MAT and July temp), and makes the use of MaxEnt circular. From my understanding, MaxEnt and other presence-only models generally work better when selecting a random set of background points, constrained by geographic space (see Barbet-Massin et al. 2012, Methods in Ecology and Evolution, and Whitford et al. 2024, Ecological Modelling).
Response 29: We agree that background points in presence-only SDMs should not be interpreted as true absences and that selecting pseudo-absences based on environmental dissimilarity can impose a priori niche assumptions. Our intent was not to define absences by climate, but to restrict background sampling to an area where the target ASV had a realistic probability of being detected given the strongly biased survey/detection footprint in the Arctic.

We have revised the Methods to clarify that the outer buffer defines an "accessible/sampled" background domain (geographically constrained background; Barbet-Massin et al. 2012) and that background points are sampled from the annulus between an inner buffer (to avoid sampling from the same grid cell as occurrence records) and the outer buffer. This reduces the risk of drawing background points from largely unsurveyed parts of the Arctic, where non-detections would be uninformative and could confound suitability with sampling bias.

Regarding the OCSVM step: we agree that using a classifier trained on the same climatic predictors can be perceived as circular if it is interpreted as defining "absences." We have therefore removed any interpretation of OCSVM-selected points as absences. Instead, the OCSVM is used only as an environmental filter within the geographically constrained background domain to avoid selecting background points with highly similar climate conditions to presences, thereby increasing contrast between presences and available conditions.

Below, we present an example comparing background selection methods: using OCSVM within an annulus versus without OCSVM. The results demonstrate that: models with OCSVM background selection yield relatively higher suitability values across the study area compared to models without OCSVM; the predicted extent of suitable habitat is larger when using OCSVM; but the spatial patterns of relative suitability remain consistent between approaches, as regions with high suitability under OCSVM also show high suitability without OCSVM.

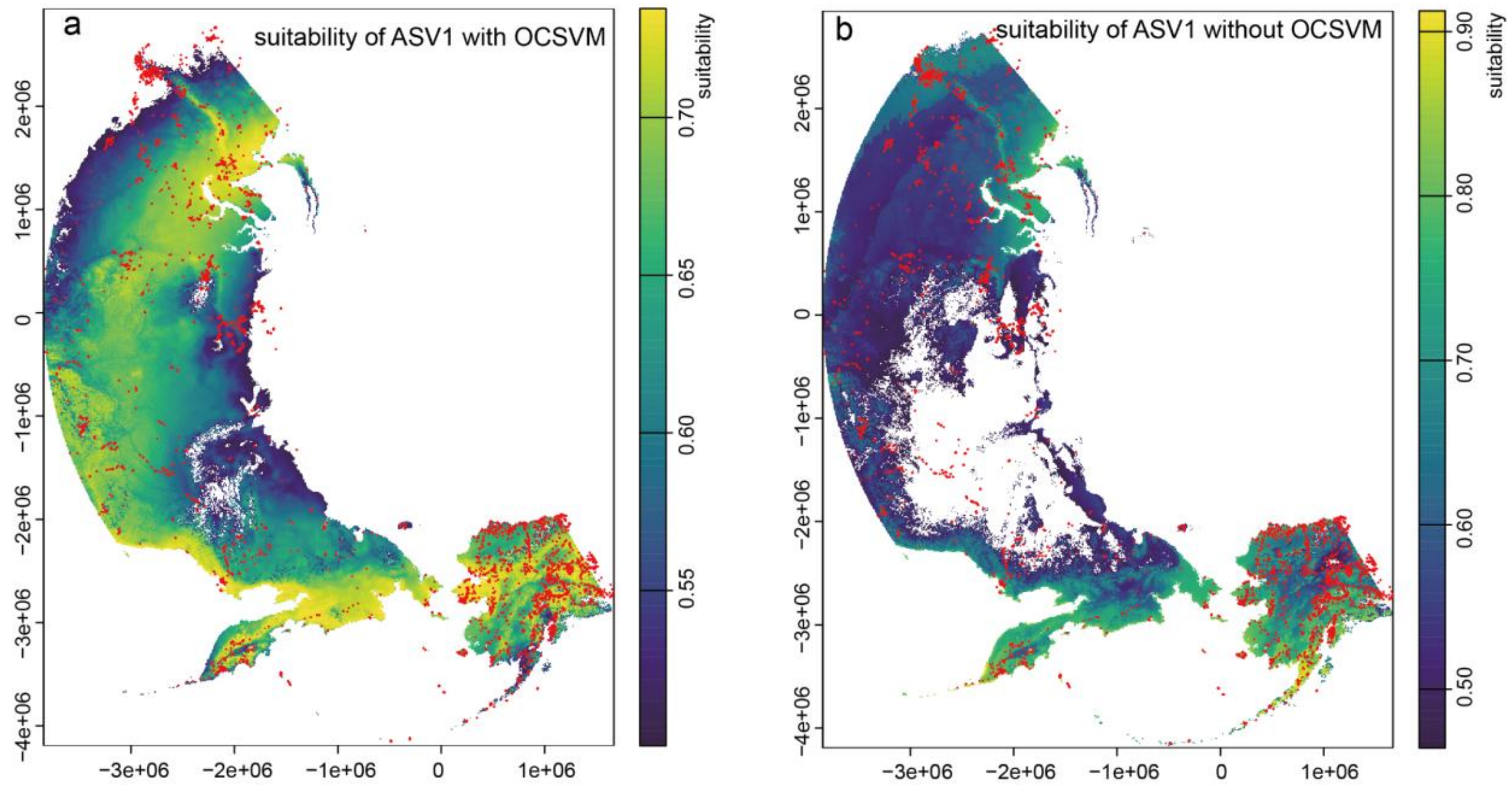

Supplementary Figure 12. Predicted habitat suitability (colour scale) from species distribution models and observed taxon occurrences (red dots). The left panel shows results with background selection using one-class support vector machine (OCSVM), while the right panel shows results without OCSVM background selection.

Habitat suitability represents the probability of taxon occurrence under the null model. For regions where the taxon actually occurs, the relatively higher suitability values generated by OCSVM background selection (though still indicating low absolute probability) better reflect the reality that occurrence is possible but not guaranteed. In contrast, suitability values of zero predicted by models without OCSVM indicate absence, which contradicts empirical observations of presence. Therefore, the relatively higher suitability values produced by OCSVM-based background selection are more appropriate for downstream analyses, as they better capture the probabilistic nature of species occurrence.

Importantly, we provide a sensitivity analysis in the Supplement (Supplementary Figure 13, Supplementary Figure 14) using purely random background points within the same buffered domain (i.e., without OCSVM filtering). As expected, this approach shifted the dominant plant interaction values to more negative during the Holocene. Still, the general pattern remains robust regardless of whether OCSVM filtering is applied.

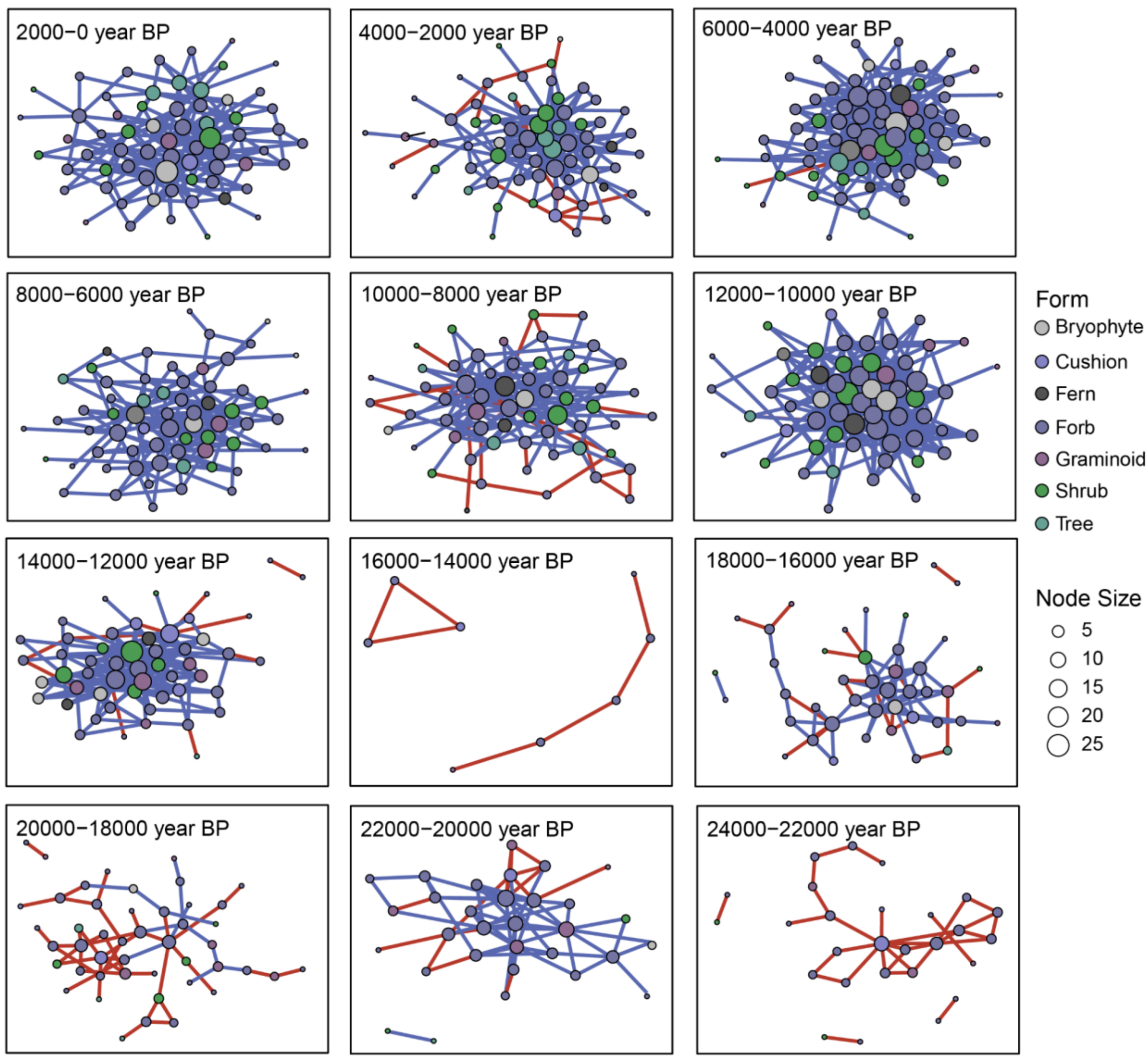

Supplementary Figure 13. Temporal dynamics of the plant interaction network across 2000-year time slices, using background point selection without the one-class support vector machine (OCSVM). Nodes represent taxa, with colours indicating different growth forms. Edges between nodes represent interactions among taxa, with red edges indicating positive interactions and blue edges indicating negative interactions. Node size corresponds to the number of connections (i.e., node degree).

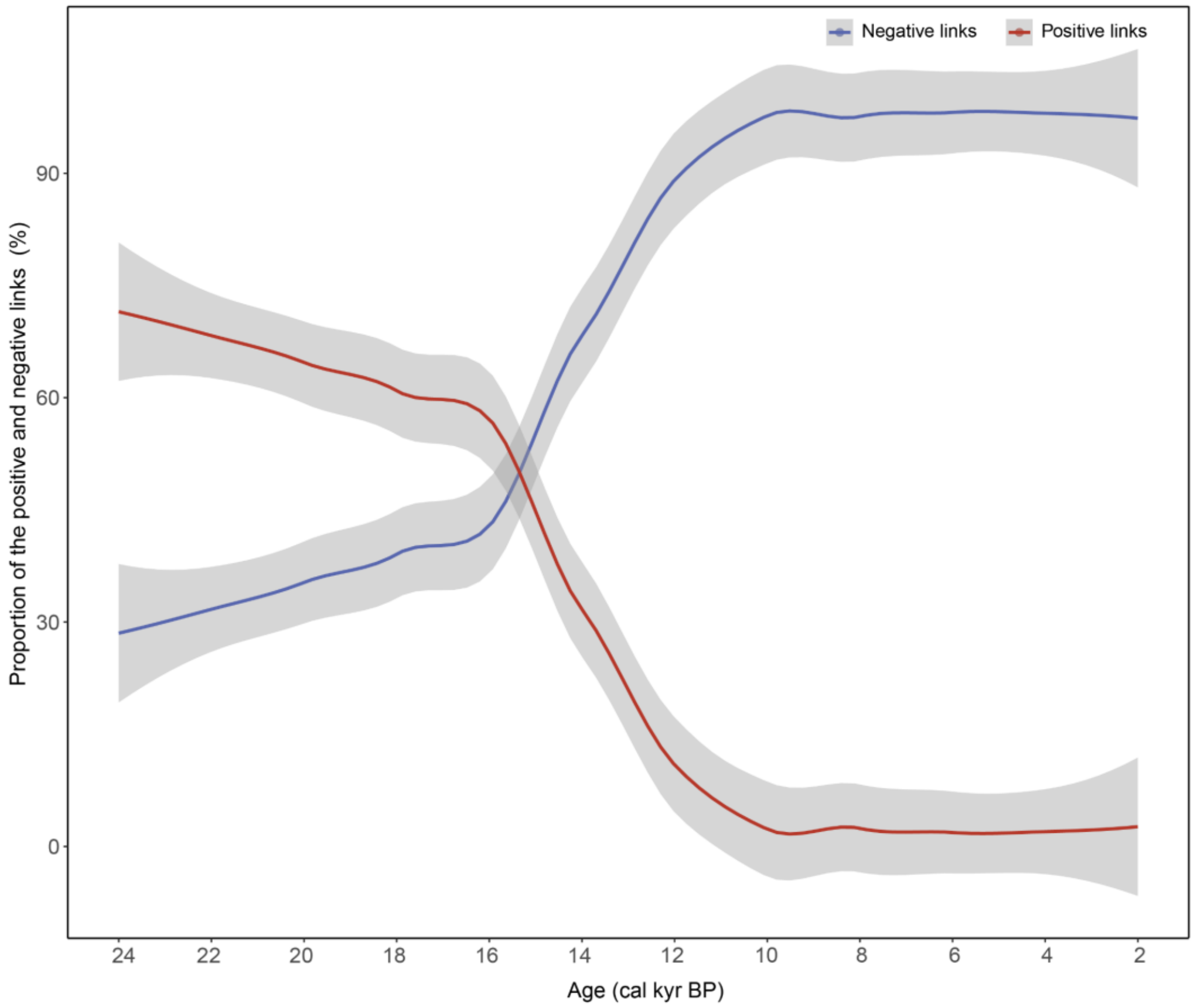

Supplementary Figure 14. Temporal trends in the relative proportion of positive and negative interactions, using background point selection without the one-class support vector machine (OCSVM).

Comment 30: Line 498: I'm glad to see the Boyce Index being used to evaluate model performance! Was it calculated using a sliding window or discrete bins? Also, Looking at the supplementary Figure 4, it looks like some models were no better than random and some were even worse than random. My guess is that these models performed poorly because of the geographic constraints put on the background points, leading to overfitting and non-transferability. Were the species with those models still included in the environmentally constrained null model?

Response 30: The Boyce index presented in the boxplot was calculated from the Boyce value of each ASV, based on ASV occurrences and predictions derived from the SDM. It was therefore not calculated using sliding windows or bins. The median Boyce index value is 0.91, indicating strong model performance. Outliers account for 1.34% of all observations, representing a minor proportion of the dataset. These outliers were retained in the environmentally constrained null model; however, we also reran the null model excluding them. The results are presented below and show a pattern consistent with that of the full dataset, suggesting that the outlying Boyce index values did not substantially influence the overall results.

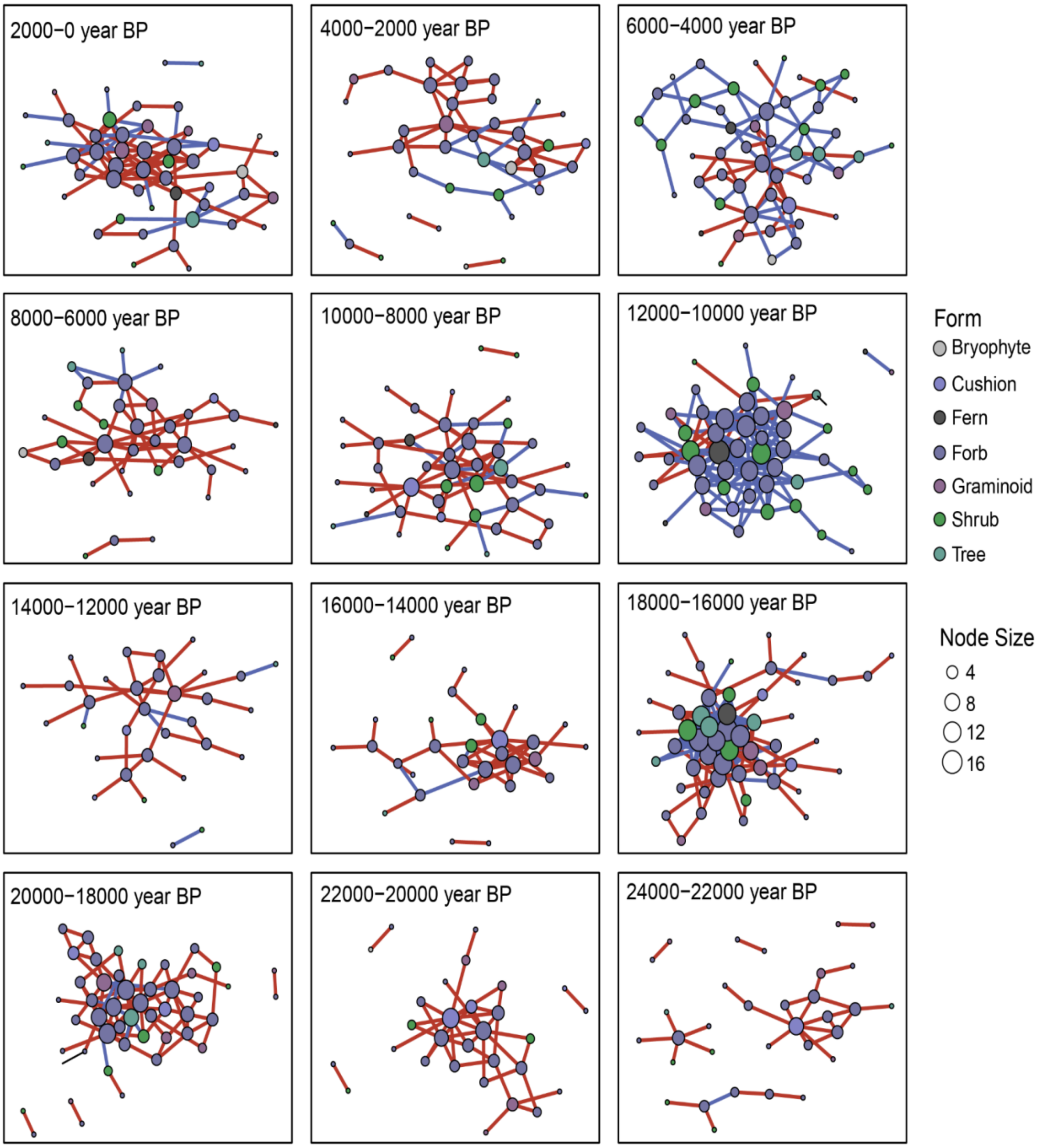

Supplementary Figure 15. Temporal dynamics of the plant interaction network across 2000-year time slices, using background point selection with the one-class support vector machine (OCSVM), with ASV exhibiting outlier Boyce index values excluded. Nodes represent taxa, with colours indicating different growth forms. Edges between nodes represent interactions among taxa, with red edges indicating positive interactions and blue edges indicating negative interactions. Node size corresponds to the number of connections (i.e., node degree).

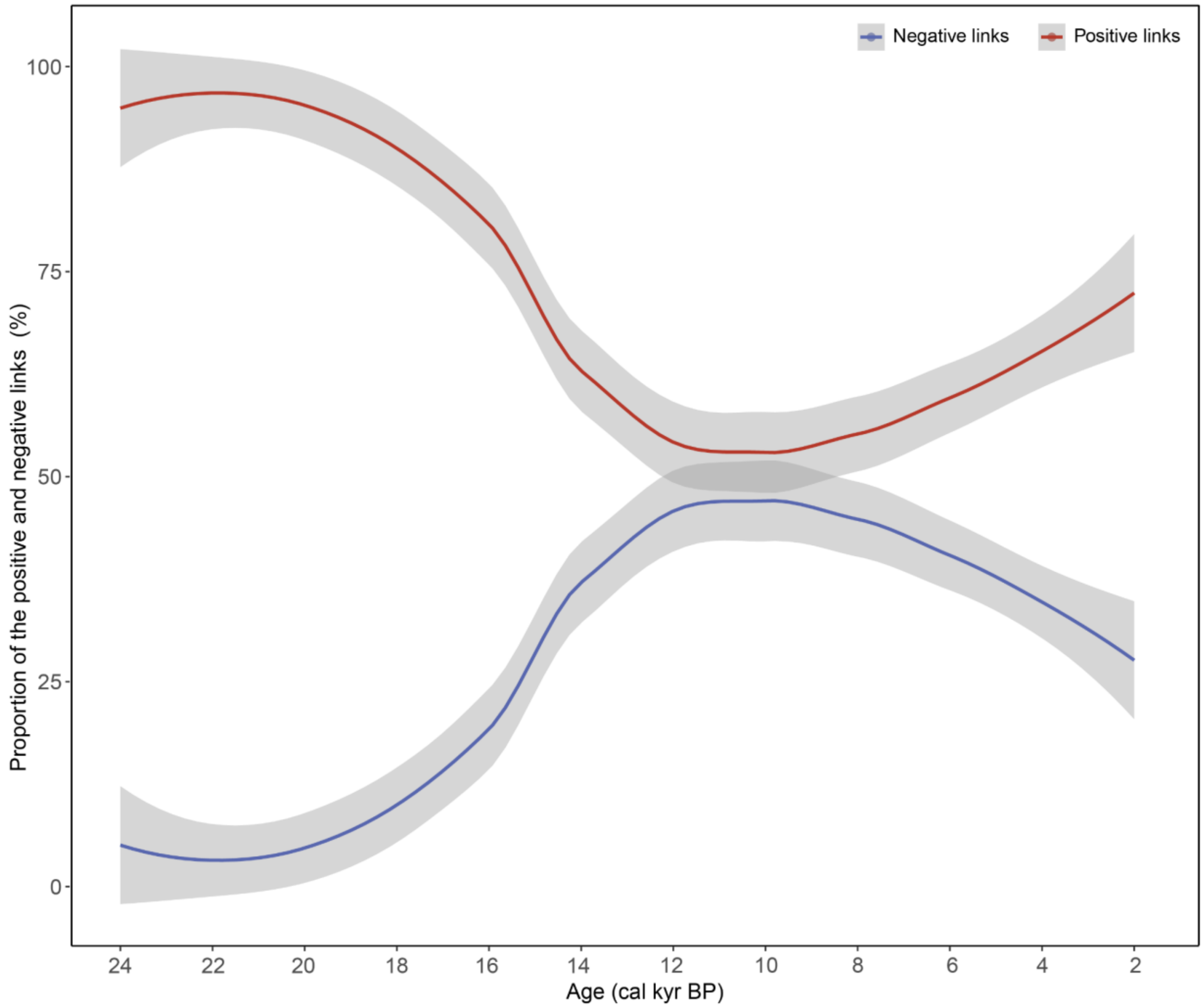

Supplementary Figure 16. Temporal trends in the relative proportion of positive and negative interactions, using background point selection with the one-class support vector machine (OCSVM), with ASV exhibiting outlier Boyce index values excluded.

Comment 31: Line 510: Not 2,000 year increments, like the time slices of the empirical data?
Response 31: Suitability was predicted based on a 10,000-year time slice. To ensure sufficient samples for the environmentally constrained null model, samples within a 2,000-year window were used. Consequently, samples from the same site but belonging to different 1,000-year time slices were treated as distinct samples.

Comment 32: Line 524: There is a missing parenthesis somewhere in this equation.
Response 32: Exactly, we added it.

Comment 33: Line 530-531: The environmentally constrained null models have not been explained yet; I would put this sentence after the explanation.
Response 33: agree, done.

Comment 34: Line 540: why 22 bins? Also, pairwise C-score of the null model, correct?

Response 34: Too few bins would reduce resolution, while too many bins would result in few species pairs per bin. Previous studies used 22 bins, and given that our dataset includes at most 18 samples per time slice, resulting in potentially 616 unique C-score values, 22 bins allow for a relatively even distribution of C-scores. Therefore, we consider 22 bins reasonable for our study. Nevertheless, the choice is not fixed: we also tested 21 and 20 bins, and found that the overall patterns remained unchanged. And the pairwise C-score for both the sedaDNA data and the null plant community, we revised the text.

New text: Line 614-616 For both the sedaDNA data and the null plant community, each pairwise C-score was assigned to one of 22 evenly spaced bins spanning the interval from 0 to 1.

Comment 35: Line 543: What does "mean limits" mean in this context?
Response 35: It refers to "the mean C-score"; we revised it.

New text: **Line 620-623** For each bin, the mean C-score value of the simulated null distribution was calculated, species pairs from the sedaDNA were ordered by their scores within each bin, and those with scores greater than the mean of the simulated distribution (Bayes M criterion) were retained.

Comment 36: Line 551-570: This is a really interesting method! I would make it a little bit more clear here that for this you're only piecing apart the influence of dispersal limitation on negative interactions (as suggested by the excellent Supplementary Figure 3).
Response 36: Thanks, revised.
New text: Line 630-632 To ensure that species segregation is not driven by the dispersal limitation (e.g., species a and species b being segregated simply due to non-overlapping distribution ranges)

Comment 37: Lines 559-560: Only looking at species pairs with non-differing centroids is a very good idea, but it could skip over some positive interactions, right? For example, if two species have different centroids but associate more than expected based on climate, wouldn't that be evidence for positive interactions?
Response 37: Thanks for the question. The association was checked first. If the observed association between two species exceeds expectations from both climate-based models and randomisations, the pair is classified as exhibiting a positive interaction directly (see the upper-right part of Supplementary Figure 3). In this case, it is not necessary to consider the centroids.

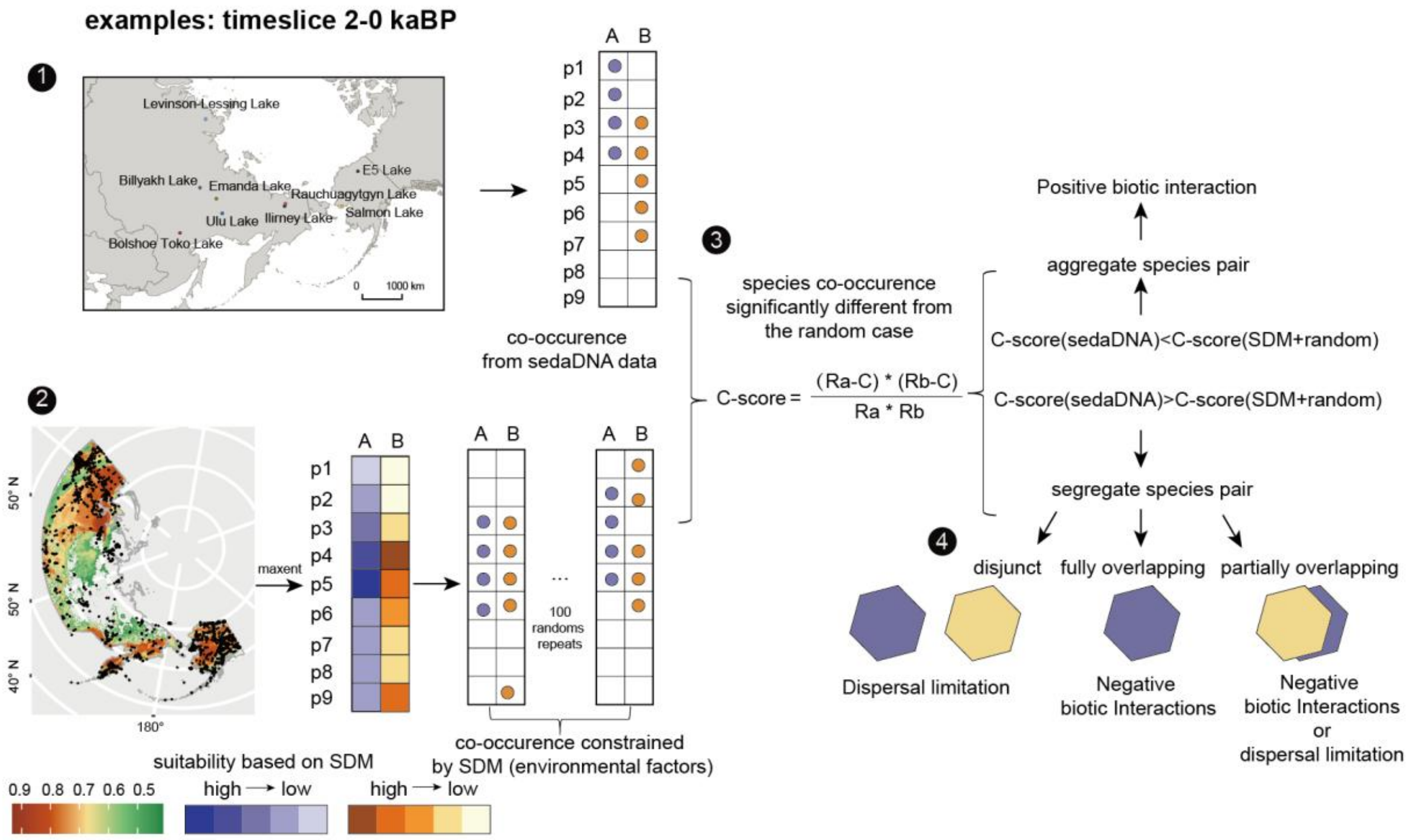

Supplementary Figure 3. Schematic diagram illustrating the disentanglement of plant interaction effects, mean annual temperature/mean July temperature effects, and dispersal limitation using an environmentally constrained null model.

Comment 38:
Figure 1:
This is a beautiful figure!

Figure 2:
I really like this figure, especially the consistent color scheme between Figures 2a-2d! Figure 2a could benefit from a legend like the others, describing which links are positive and which are negative. Also, it's a little hard to figure out which boxplots in Figure 2b correspond to which categories (maybe space them out or add dividers).

Figure 3: Maybe reword “Wood plants abundance” to “Abundance of woody plants”?
Response 38: Thanks, revised.

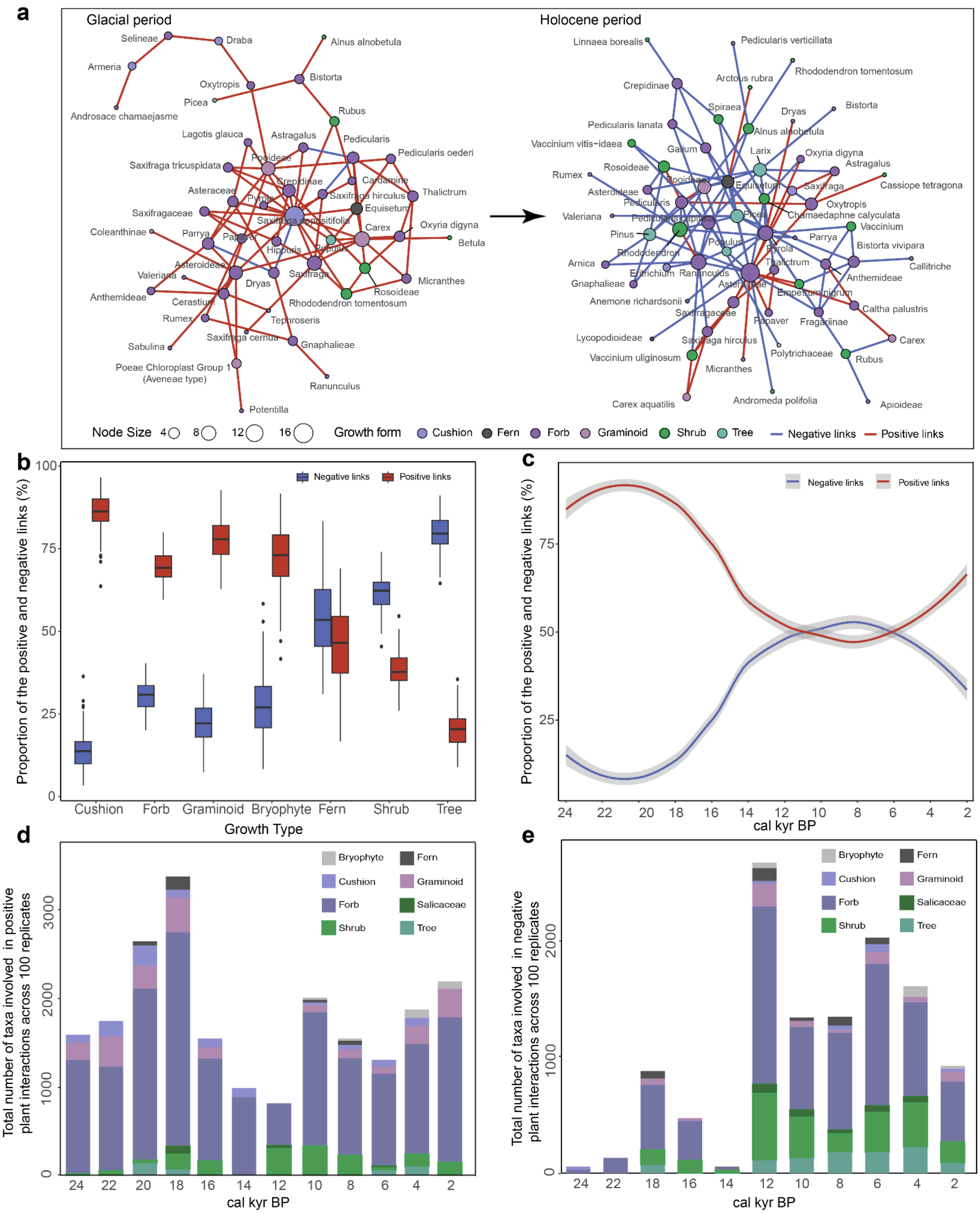

Figure 2. Dynamic plant interaction network during the glacial and Holocene periods.

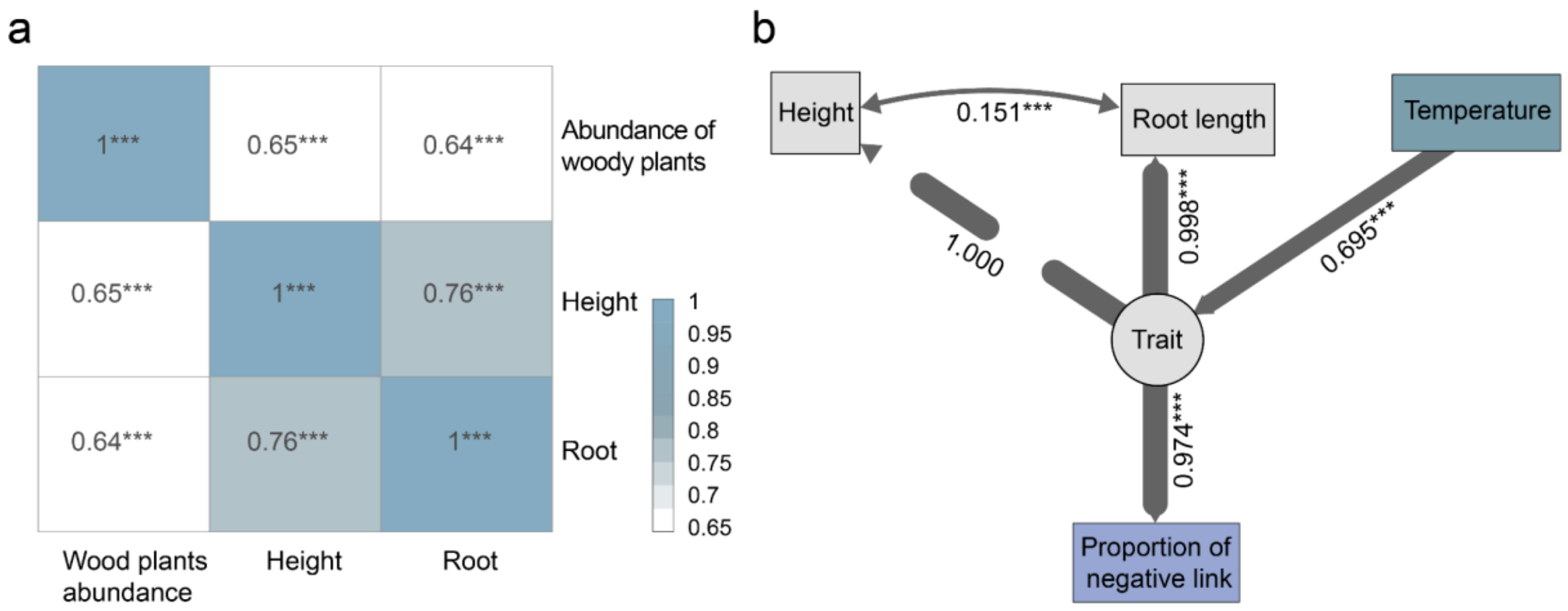

Figure 3. Structural equation model showing the relationships between response variables and predictors.

Comment 39: Figure 4: I appreciate the explanation of the relationship between interactions and beta diversity. However, I'm not sure what "distribution range of _Taxon 1_) is supposed to be telling the reader (same with High rate of taxon loss of _Taxon 1_, and "distribution range in the bottom corner").

Response 39: Thanks. We would like to show an example of a native taxon (purple circle) that exhibited a large distribution range during the glacial period, but a much smaller range, and even a high risk of local loss during the Holocene. We have revised the plot to make it clearer and easier for readers to understand.

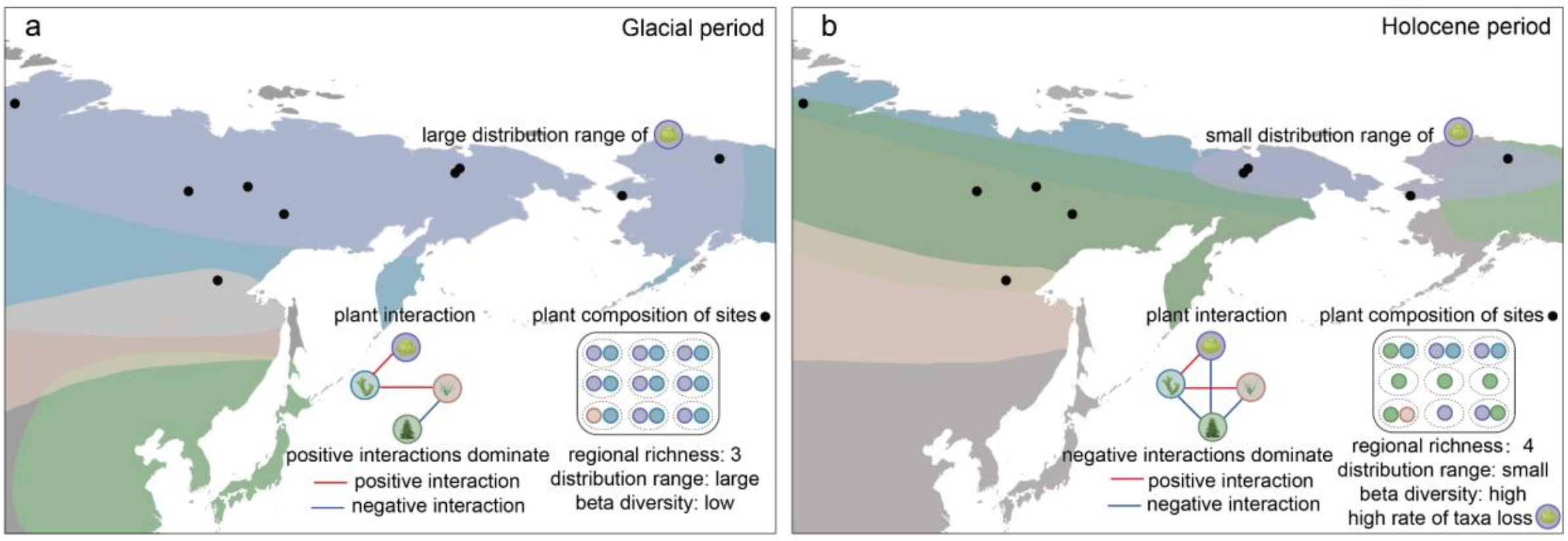

Figure 4. Schematic diagram depicting the impact of plant interactions on community.

Comment 40: Reviewer #2 (Remarks on code availability):
The code was clear and easy to read; thank you!

Response 40: Thanks.

Reviewer #3 (Remarks to the Author):

Review of the manuscript entitled "Woody plant encroachment enhances negative interactions in Arctic plant communities over the last 24,000 years"
This manuscript analyzes past plant interactions using sedaDNA records from nine lakes distributed across Siberia and Alaska. To achieve this, the authors transformed plant composition tables into plant functional traits (specifically root length and plant height), traits known to shape community structure through their role in competition for light, water, and nutrients. They used environmentally constrained null models to differentiate between biotic interactions and co-occurrence patterns driven by environmental filtering. They also integrated species distribution models to account for species dispersal limitations.
To my knowledge, such a question has not yet been addressed from a paleoecological perspective, which makes this study highly original. The conceptual framework and the statistical approaches proposed are, in my view, very relevant (although I am not in a position to assess the mathematical details behind the models).
The main conclusion of the paper is that colder glacial periods favored positive plant interactions (facilitation), whereas warmer conditions led to more negative interactions (competition). This result aligns well with the Stress Gradient Hypothesis in ecology. Furthermore, the authors show that facilitation mainly involved herbaceous taxa, while competition was more frequently associated with woody species, likely due to competition for light and access to water/nutrients. These interaction patterns, well-documented in ecological studies, reinforce the robustness of the inferred interaction networks across different growth forms. The study also highlights the indirect role of temperature in modulating plant-plant interactions.
These conclusions are significant in the context of ongoing global warming and are broadly relevant to several scientific communities, including ecology, paleoecology, and sedaDNA research. I particularly appreciated the section addressing the limitations of the approach and potential biases in the inferences.
Response: We are thankful to Reviewer #3 for the constructive comments.

Specific comments and questions:

Comment 1: • P3, L82–83: I would avoid stating that the transition from the glacial period to the Holocene is an analogue to current global warming. I suggest modifying to: "may provide an analogue", as you do in the abstract. This is especially important since, although the area was largely deglaciated during the glacial period, soil development (e.g., weathering processes, soil thickness, nutrient availability, and water retention) was likely very different from today.
Response 1: Agree, revised.
New text: Line 85-87 The shifts in plant communities and interactions from the glacial period to the Holocene, driven by rising temperatures, may provide an analogue for contemporary ecological change in the Arctic.

Comment 2: • Figure 1C: This panel shows Salicaceae, which is a taxonomic group, not a growth form. Please adjust the legend accordingly. Also, I'm not entirely sure why you chose to include Salicaceae specifically—could you justify this choice? If not essential, consider

removing it. Alternatively, you could also include Ericaceae, which likely increased during the Holocene with podzol development. These taxa are also mentioned later in the section "Effects of woody encroachment on plant interaction shifts."

Response 2: Thanks for this suggestion. Salicaceae are mostly shrubs in our study region, and they tend to be overrepresented in the sedaDNA data, with high read numbers. To clarify for the general audience: during the Glacial period, shrubs were generally of low abundance; while other shrubs had relatively few reads, Salicaceae showed comparatively high reads due to this overrepresentation and its occurrence along the lake shore, which is why it was shown specifically here. But you are right, Salicaceae is not a growth form, therefore we clarified it in the caption.

New text: Line 144-147 Temporal dynamics in the proportion of transformed counts, representing the relative abundance of distinct plant growth-forms, are depicted in different colours. Within the shrub growth form, Salicaceae was shown specifically due to its high read counts.

Comment 3: • P7, L140–142: When you state "60% of the flora…", it reads as if you're referring to actual landscape composition, whereas this is in fact the proportion within your sedaDNA record. Please clarify.

Response 3: Thanks, revised.

New text: Line 152-155 The community was dominated by herbaceous taxa prior to 14.0 cal ka BP, with forbs comprising approximately 60% of the total plant abundance, while cushion plants and graminoids each accounted for less than 10%.

Comment 4: • P8, L182–185: What exactly do you mean by "broad taxonomic involvement"? Do you mean a large number of taxa were involved? If so, it would help to give more examples, beyond Saxifraga oppositifolia.

Response 4: Yes, a large number of taxa were involved, we revised the sentence.

New text: **Line 194-197** From the perspective of interaction direction, positive plant interactions dominated during the glacial period, peaking around the Last Glacial Maximum (20–18 cal ka BP), and involving a large number of taxa, for example, *Saxifraga oppositifolia*, *Saxifraga tricuspidata*, and *Dryas* (Figure 2a, 2d, Supplementary Figure 2).

Comment 5:• Figure 2: Perhaps I missed it, but how exactly was the network constructed? Which software or R package did you use?

Response 5: We are sorry for the missing information and have now provided it.

New text: Line 641-653 For each timeslice and each resampling of the sedaDNA data, the environmental-constraint null model was applied to identify species pairs driven by biotic interactions. An unweighted plant interaction network was then constructed from these species pairs using the igraph R package [91-93]. In the network, aggregated species pairs were assigned a correlation value of 1 (positive interaction), segregated pairs a correlation value of –1 (negative interaction), and all other pairs a correlation value 0 (no interaction). The final plant interaction network was visualized with the ggraph R package [94], and for one randomly selected resampling, two representative networks are shown in Figure 2a.

Boxplots of the proportions of positive and negative interactions across different growth-form taxa, temporal trends in the relative proportions of positive and negative interactions, and temporal variation in the number of taxa of different growth forms engaged in positive and negative interactions were calculated across all 100 resamplings and visualized with the ggplot2 R package [73] (Figure 2b–e).

Comment 6: • P12, L259–261: I'm not sure I understand what you mean by "resolution of network". Also, I'm wondering how the use of 2,000-year windows might affect your inference of plant-plant interactions. I understand that this choice helps reduce age-depth uncertainties and increases the number of data points, but doesn't it risk creating interactions between taxa that didn't actually co-occur? Perhaps I misunderstood, or perhaps taxonomic composition is relatively stable within each time window. In any case, I suggest discussing this point further for clarity.
Response 6: Agree, have added a discussion of these points.
New text: Line 278-281 Although a 2,000-year time slice reduces age–depth uncertainty and increases sample size relative to shorter intervals, and plant composition within such windows is broadly homogeneous, it risks introducing false co-occurrences that may overrepresent positive plant interactions.

Comment 7: • L300–301: It's not immediately obvious that "the spread of woody taxa facilitated the formation of mycorrhizal associations." Could you elaborate on this mechanism? Perhaps the following sentences aim to clarify this, but currently it's a bit unclear. A reformulation or brief explanation would improve readability here.
Response 7: Agree, revised
New text: **Line 328-331** Additionally, their long-lived root systems facilitate the formation of mycorrhizal associations by providing fungi with suitable hosts and continuous access to photosynthates [48], which in turn enhance nutrient and water uptake by the plants.

Comment 8:• Lastly, I don't know whether modern studies have shown that certain plant species can switch the direction of their interactions (from positive to negative or vice versa) depending on environmental context. But I found this finding particularly interesting—perhaps even fascinating (also, I recently saw my first Cassiope tetragona!). If such interaction plasticity has been observed in modern systems, it would be great to cite relevant studies. If not, this is a remarkable result that deserves to be emphasized more strongly, to my mind.
Response 8: Modern studies have observed that the same plant species can shift the direction of their interactions depending on environmental conditions. For example, B. rupestre and A. elatius were facilitated under stressful conditions (without additional water), whereas in less stressful conditions (with increased water availability), facilitation of B. rupestre and A. elatius was suppressed, and B. erectus experienced competitive exclusion (Liancourt et al., 2005). We have already cited the new related reference.

Liancourt et al., P. Liancourt, R.M. Callaway, R. Michalet. Stress tolerance and competitive-response ability determine the outcome of biotic interactions. Ecology, 86 (6) (2005), pp. 1611-1618, 10.1890/04-1398

Comment 9: Reviewer #3 (Remarks on code availability):

I did not check all the codes, just have a Quick Look. but I think that a readme file is missing. Or I missed it.
Response 9: We added the README file now.

REVIEWER COMMENTS

Reviewer #1 (Remarks to the Author):

The authors have addressed my comments sufficiently and I applaud them for the revision of an excellent manuscript. (Remarks on code availability)

Response: We are thankful to Reviewer #1 for their evaluation and are pleased that our revisions have addressed the reviewer's concerns.

Reviewer #2 (Remarks to the Author):

Thank you for giving me the opportunity to read the revised version of this manuscript. I am very impressed by the work done between the submissions, and I believe the manuscript is much improved. I only have a few small notes to highlight, but otherwise, I think it is an important contribution to the field and a very well-written work. It was a pleasure to read!

Response: We are thankful to Reviewer #2 for their constructive comments.

Comment 1: Line 79: "..shape the plant interactions." Maybe revise to "...shape how plants interact."?

Response 1: revised. New text line:79.

Comment 2: Lines 83, 89: I think in these two places, putting a time on the glacial period, Holocene, and Last Glacial Maximum (for example, and using rough time estimates; "... from the glacial period (>14,000 years ago) to the Holocene (~11,000 years ago)" can be useful to orient the reader.

Response 2: revised. New text line: 83,84,89.

Comment 3: Line 96: Environmentally constrained null model --> Environmentally constrained null models

Response 3: revised. New text line: 97.

Comment 4: Line 99: "It proivdes insight..." --> "They provide insight..."

Response 4: revised. New text line: 100.

Comment 5: Line 241: "Consistently..." Maybe "Consistent with these observations..."? You talk about consistency through time as well in the paragraph, but I think here you're discussing how your results are consistent with previous work/observations, so maybe a little clarification is needed.

Response 5: revised. New text line: 200.

Comment 6: Lines 275-277: This caveat'limitation is about the SDMs, so maybe move it further up in the paragraph (where you talk more about the SDM methods, etc.)? Also, I think this paragraph could be split into two to make it easier to read and not as much of a list: one paragraph focusing on data limitations (where you talk about, e.g., the sedaDNA, taphonomy, and the temporal resolution, etc.), and model limitations.

Response 6: revised. New text line: 213-241.

Comment 7: Figure 4 caption: "impact of plant interactions on community" --> either "communities", or something slightly more specific like "community structure"(Remarks on code availability)
Response 7: revised. New text line: 9972.

Reviewer #3 (Remarks to the Author):

All the comments have been addressed and I consider that the manuscript is now ready for publication.(Remarks on code availability).
Response: We are thankful to Reviewer #3 for their evaluation and are pleased that our revisions have addressed the reviewer's concerns.
